# Molecular factors controlling charge pair generation in organic photovoltaic materials

**DOI:** 10.1038/s41563-026-02509-6

**Published:** 2026-02-27

**Authors:** Lucy J. F. Hart, Daniel G. Medranda, Shi Wei Yuan, Linnea Lindh, Jolanda S. Müller, Hanbo Yang, Hugo Gerard, Tianyu Zhao, Arianna Quesada-Ramirez, Mariano Campoy-Quiles, Mohammed Azzouzi, Flurin D. Eisner, Jenny Nelson

**Affiliations:** 1 Department of Physics, Imperial College London, London, UK; 2 Ecole Polytechnique, Palaiseau, France; 3 Department of Nanostructured Materials, Institut de Ciència de Materials de Barcelona, ICMAB-CSIC, Bellaterra, Spain; 4 Laboratory for Computational Molecular Design (LCMD), Institute of Chemical Sciences and Engineering, Ecole Polytechnique Federal de Lausanne (EPFL), Lausanne, Switzerland; 5 Department of Chemistry, Imperial College London, London, UK; 6 School of Engineering and Material Science, Queen Mary University of London, London, UK

**Keywords:** Solar cells, Semiconductors, Atomistic models

## Abstract

Through remarkable advances in materials design, the efficiency of photovoltaic energy conversion in molecular materials has risen from 1% to over 20% within 2 decades. Some recent reports argue that charge photogeneration can occur directly in neat films of the best-performing molecular materials, and that this process may assist current generation in heterojunction devices. Here we address this assertion by combining experimental measurements of charge generation in single-component and heterojunction devices with a computational model of the generation and evolution of delocalized excited states in such systems. We identify key molecular parameters that are likely to assist charge generation in high-performance materials, including the exciton binding energy, reorganization energy, energetic disorder, electronic coupling and the molecular packing motif. We show that including state delocalization is critical to the results. While we find that charge generation in single domains is unlikely to drive photocurrent generation in low-offset heterojunctions, the same molecular parameters favour charge generation in both device architectures.

## Abstract (web summary)

A model is devised to investigate free charge generation in neat acceptor domains and at donor–acceptor heterojunctions. Efficient photocurrent generation in organic heterojunctions at low energetic offsets can be related to key molecular parameters and to electronic state delocalization.

## Main

The performance of organic photovoltaic (OPV) devices based on heterojunctions between electron-donating and electron-accepting organic semiconductors has traditionally been limited by the need for an offset (hundreds of millielectronvolts) in the redox potentials of the donor and acceptor^1,2^ to split the tightly bound photogenerated exciton into free charges. Recently, the emergence of fused-ring electron acceptors such as Y6 (ref. ^3^) has increased the power conversion efficiency (PCE) of OPVs from 14% to over 20% using heterojunctions with surprisingly small offsets^4–8^.

The success of Y-family acceptors and other non-fullerene acceptors (NFAs) has been attributed to properties including strong and sharp optical absorption^9^, high and ambipolar charge-carrier mobility^10^, ordered molecular packing^5,7,8,10^, low exciton reorganization energies^11^, and long exciton lifetimes and diffusion lengths^12–14^. Interesting recent reports suggest that free, or loosely bound, charge-transfer (CT)-like charges may be photogenerated in Y6 domains in the absence of a heterojunction^10,15–22^ and that this intrinsic photogeneration helps to explain the relatively efficient photocurrent generation at low interfacial offsets in Y-based heterojunctions. This phenomenon has been attributed to the close and well-ordered packing of Y6 in thin films, leading to strong intermolecular interactions and excited states with CT character^10,15,16,19,23–25^, as well as to the molecules’ intramolecular donor–acceptor structure^26–28^. However, photogenerated excited states with CT character have been observed in neat films of both IT-4F and IEICO-4F (refs. ^29,30^), suggesting that Y-family acceptors are not unique in this regard. Furthermore, some degree of free charge generation has been observed in thin films of fullerenes^31–34^, conjugated polymers^35–41^, molecular donors^42^ and other small molecular acceptors^29,43–45^. However, neat Y6-based photodiodes generate photocurrent at relatively low electric fields compared with other fused-ring acceptors^30^.

A key probe of charge separation in organic semiconductors is the response of the device photocurrent to an applied electric field. Experimental and computational research into the electric field dependence of photocurrents in single organic semiconductors^40^ and at heterojunctions^2,39,46,47^ has shown that an applied reverse bias can assist exciton dissociation in a heterojunction device, as well as assisting charge collection^2,33,39,40,46–48^. The applied bias affects charge generation more strongly in single-component devices than in heterojunction devices^40,49^, leading to a characteristic sigmoidal dependence of the internal quantum efficiency (IQE) on the applied electric field, as previously rationalized within Braun–Onsager theoretical frameworks^50–52^.

Traditional frameworks represent charge generation in OPV devices in terms of transitions between excitonic, CT and charge-separated (CS) states, with energies modulated by a heterojunction or an electric field. However, such frameworks are insufficient to model the process of charge generation in materials where strong electronic coupling delocalizes and mixes states. Therefore, to understand the behaviour of the highest-performing OPV devices and the impact of intradomain charge separation on photocurrent generation at a heterojunction, a more detailed treatment is needed.

In this Article we address the relationship between free charge generation in single-component molecular acceptor films and that at donor–acceptor heterojunctions, and consider whether the conditions that favour intrinsic charge generation also favour the charge generation efficiency (CGE) in low-offset heterojunctions. To interpret the electric field and offset dependence of charge generation in single-component and planar heterojunctions, respectively, we introduce a model that combines a quantum description of the system’s kinetics with thermodynamic considerations. This framework allows us to investigate single-component and heterojunction device performance as a function of material parameters. The work provides a rationalization for the behaviour of some of the highest-performing OPV systems in terms of favourable molecular parameters, such as the high electronic coupling and low exciton reorganization energy of Y6, and suggests design rules to achieve further improvements.

## Framework for modelling charge generation with delocalized states

**Fig. 1:**
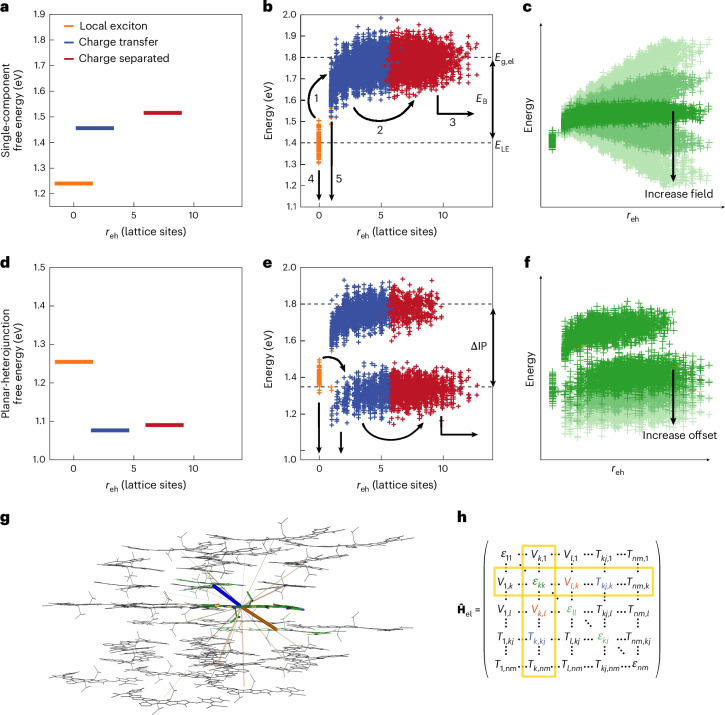
Excited-state model and its application to single-component devices and heterojunctions. **a**, Schematic state diagram showing the free energies of the LE, CT and CS states in a single-component organic semiconductor. The legend in **a** also applies to **b**, **d** and **e**. **b**, Detailed state diagram for a molecular crystal showing the energies of each singly excited state plotted against its average *r*_eh_ (see Methods for categorizations of states). *E*_g,el_ is the electronic bandgap and *E*_LE_ is the local exciton energy, which give the exciton binding energy *E*_B_ = *E*_g,el_ – *E*_LE_. The numbered arrows indicate the key steps in charge generation: (1) formation of a tightly bound CT state from the photoexcited exciton; (2) separation of the CT state; (3) extraction of separated charges; (4) geminate recombination of LE states; and (5) CT recombination. Free energies in **a** correspond to the three ensembles of microstates in **b** calculated using a canonical ensemble as described in Methods. **c**, Illustration of the effect of increasing the applied electric field on the eigenstate distribution for a single-component simulation. Lighter shades of green indicate a stronger electric field. **d**, Schematic three-state diagram showing the free energies of LE, CT and CS states for a heterojunction. **e**, Calculated eigenstates for a planar heterojunction plotted as energy against *r*_eh_, categorized as in **b**. The corresponding free energies in **d** are derived from the microstate ensembles in **e**. The HOMO offset (ΔIP) between the donor and acceptor is indicated. **f**, Illustration of the effect of increasing ΔIP on the energetic distribution of states for a planar heterojunction. Lighter shades of green indicate a larger ΔIP. **g**, Unit cell of a hypothetical crystal structure in which examples of nearest-neighbour intermolecular interactions between the green-coloured molecules are highlighted in orange for excitonic interactions and blue for electronic interactions. Other possible intermolecular interactions are indicated by yellow lines. **h**, Information on the molecular sites and interactions in the crystal are encoded in the elements of the tight-binding Hamiltonian. In this Hamiltonian, *ε*_*ij*_ are the site energies, *V*_*ij*_ are the couplings between excitonic states, and *T*_*i,nm*_ and *T*_*ij,nm*_ are the electronic couplings describing exciton-to-CT and CT-to-CT transitions, respectively. The elements of the Hamiltonian corresponding to the interactions and site energies shown in **g** are highlighted using the same colour scheme.

Charge generation in organic semiconductors is often described as a multistep process, involving three discrete states (local exciton (LE), CT and CS), as illustrated in Fig. 1a for a single-component semiconductor and in Fig. 1d for a heterojunction. Although the three-state picture provides a useful conceptual framework, it does not capture the impact of delocalization and disorder on the state distribution and on potential barriers to charge separation. In practice, an ensemble of states is involved, each of which may extend over multiple sites in the molecular assembly. These states can be classified in terms of their energies and average electron–hole separation, *r*_eh_ (defined in Supplementary Note 1, Section 1.2). This full ensemble of states is illustrated in Fig. 1b, highlighting the key processes: (1) dissociation of a photogenerated LE state (orange) to an intermolecular CT state (blue); (2) CT separation to form a CS state (red); (3) charge transport and collection; and (4–5) competing recombination pathways.

To simulate the dynamics in strongly coupled materials, we extend previous work^53–58^ to develop a model that can describe delocalized states in arbitrary molecular systems (full details are provided in Methods and Supplementary Note 1). Molecular domains or heterojunctions are represented by a lattice of molecular sites, each defined by the energies of the highest occupied molecular orbital (HOMO) and lowest unoccupied molecular orbital (LUMO) drawn from a Gaussian distribution and intersite couplings determined by the particular molecular structure (Fig. 1g,h). The singly excited eigenstates are found by solving a tight-binding Hamiltonian in the electron–hole basis. Supplementary Fig. 8 shows an example of the ensemble of states calculated for a crystallite of the NFA Y6.

Steady-state populations under illumination or an applied electric field are computed by solving a master equation that accounts for population transfer between eigenstates, the decay of the eigenstates and extraction; entropic factors are inherently included. From the resulting eigenstate occupancies, the CGE is defined as the probability that a photoexcited state will evolve into a CS state with a binding energy below the thermal energy. This calculated CGE is comparable to the experimental photocurrent IQE, and includes both the process of exciton splitting to form states with CT character and their subsequent separation into free charges. As the simulated systems are relatively small, non-geminate recombination is neglected; light-intensity-dependent measurements confirm that this approximation is valid for the material systems studied (Supplementary Figs. 13–15).

Figure 1b shows eigenstate energies for a typical single-component system as a function of *r*_eh_. Eigenstates with largely excitonic character are found at *r*_eh_ = 0, with their energies spread by disorder and excitonic coupling. A wide band of eigenstates develops from tightly bound, nearest-neighbour CT states at small *r*_eh_ to loosely bound CS eigenstates at large *r*_eh_. The variation in eigenstate energies with *r*_eh_ roughly follows the electrostatic potential, but with large variations in energy due to electronic coupling and disorder. Figure 1c shows how an applied electric field affects the eigenstate energies and thus modulates the energy barrier between excitonic or tightly bound CT states and CS states.

Planar heterojunctions (Fig. 1e) are modelled by defining half of the lattice sites as acceptors and half as donors. Donor sites are given a large optical bandgap (3.0 eV) and weaker electronic coupling than the acceptor sites so that, in our simulations, they primarily accept and transport holes, allowing us to focus on how acceptor properties influence the CGE. Under these assumptions, excitonic, CT and CS eigenstates form within the acceptor domain, mirroring the distribution in Fig. 1b, while a lower energy band of interfacial CT eigenstates (*r*_eh_ > 0) appears across the donor–acceptor interface. Higher energy CT eigenstates, in which the electron density is located in the donor, are not considered due to their negligible occupation probability. Varying the offset in the ionization potential (ΔIP) mainly shifts the energy barrier between excitonic and interfacial CT states without strongly affecting the barrier between CT and CS states (Fig. 1f); this contrasts with the single-component device’s response to an applied electric field (Fig. 1c).

The model allows systematic exploration of how molecular and structural parameters (including the optical gap, exciton binding energy, electronic and excitonic couplings, electron–phonon coupling (via reorganization energies), HOMO or LUMO energies, molecular packing and energetic disorder) influence the eigenstate distribution and CGE as a function of operational or device parameters such as the applied electric field, heterojunction offset or excitation energy. It provides a platform for identifying how microscopic acceptor properties control photocurrent generation in devices. We proceed to use it, in combination with experimental measurements, to establish which parameters are responsible for enabling charge pair generation at low apparent driving forces.

## Electric field dependence of charge generation in pristine acceptor films

First we measure charge generation as a function of the applied electric field in neat acceptor devices with electron- and hole-selective interlayers that prevent exciton dissociation at the interfaces (Methods). To probe both the exciton dissociation efficiency and photocurrent generation efficiency, we measure light emission (via the photoluminescence (PL) and time-resolved PL (tr-PL)) and photocurrent generation (via the photocurrent IQE) as a function of the applied electric field. If the effects of extraction losses are negligible and the exciton population probed by our optical measurements is the same as that from which free charges are formed, we expect the electric field dependence of exciton dissociation to match that of charge separation^59–61^.

**Fig. 2:**
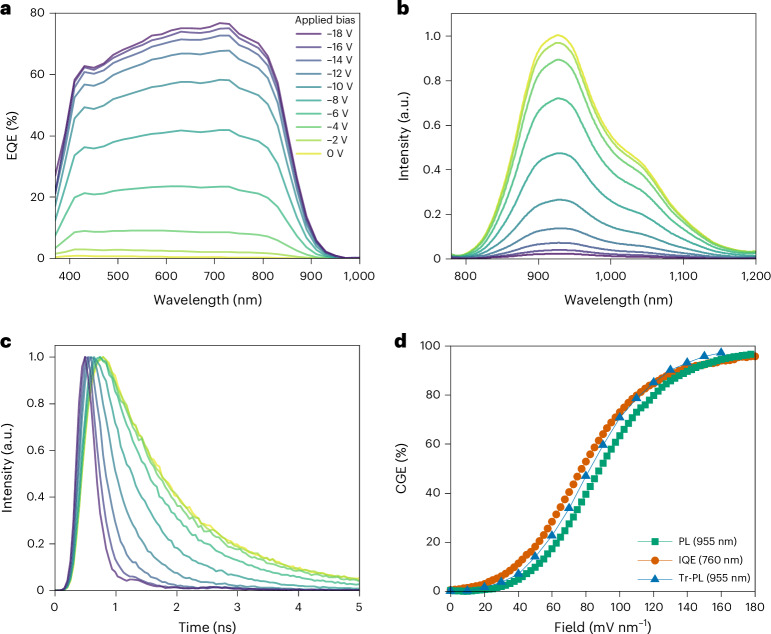
Electric field-dependent CGE of a Y-family single-component device. **a**–**c**, Reverse bias dependence of the EQE (**a**), steady-state PL spectra collected in response to 485 nm laser diode excitation, normalized to the maximum of the 0 V spectrum, (**b**) and tr-PL detected using 485 nm excitation and time-correlated single-photon counting at 955 nm, each trace normalized to its own maximum (**c**). All data measured on a Y5 device with the structure ITO/Br-2PACz/Y5(100 nm)/BCP/Ag. The legend in **a** also applies to **b** and **c**. **d**, Comparison of the electric-field-dependent IQE, PL quenching efficiency established from the steady-state PL and PL quenching efficiency extracted from the tr-PL decay rate as a function of the applied electric field using a kinetic model. Each of these measurements acts as a probe of the electric-field-dependent (free) CGE.

Figure 2a,b shows that the external quantum efficiency (EQE) and PL of a Y5 device (indium tin oxide (ITO)/(2-(3,6-dibromo-9*H*-carbazol-9-yl)ethyl)phosphonic acid (Br-2PACz)/Y5(100 nm)/bathocuproine (BCP)/Ag) increase and decrease, respectively, with an increasing reverse electric field, while the tr-PL lifetime (Fig. 2c) shortens. In Fig. 2d, we show the dependence of the device’s IQE at 760 nm under an applied electric field, which increases from 0.5% at short circuit to 93% at 0.15 V nm^−1^. The increase in IQE is accompanied by a 30-fold quenching in the PL signal, and a decrease in the tr-PL lifetime from 1.1 ns to <0.1 ns (tr-PL fits are shown in Supplementary Fig. 16). Furthermore, by relating each of the PL and tr-PL signals to a CGE (Supplementary Note 2), we find that these optically derived measures of CGE show similar electric field dependences to the IQE for this Y5 device. This indicates that exciton splitting limits the IQE, not recombination losses before charge extraction. The same set of measurements was carried out on single-component devices using three other NFAs: Y6, ITIC and IT-4F. Similar good agreement was found between the CGE curves measured using these three techniques for the ITIC and Y6 devices, but, in the case of IT-4F, the curves diverge slightly at electric fields greater than ~0.1 V nm^−1^ (Supplementary Figs. 17–21). These results demonstrate that, for electric field strengths relevant to OPV operation (<0.02 V nm^−1^), it is exciton dissociation to free charges, not recombination losses during extraction of these free charges, that limits the CGE of single-component NFA devices.

**Fig. 3:**
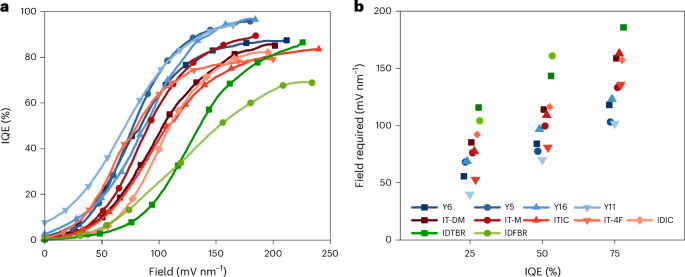
Comparison of electric-field-dependent IQE for different molecular acceptors. **a**, Electric-field-dependent IQEs for various single-component NFA devices. IQEs were measured at 760 nm for the Y family, 650 nm for the IT family, 680 nm for IDTBR and 520 nm for IDFBR. **b**, Electric field strength needed to reach IQEs of 25%, 50% and 75% for the same NFAs shown in **a** (the legend in **b** also applies to **a**). Symbols have been offset along the *x* axis for visual clarity.

Next, by comparing their electric-field-dependent IQEs, we investigate the CGE across various small-molecule acceptor families with both ADA- and ADA′DA-type cores (where A is acceptor and D is donor)^26–28^. We focus on families of NFAs used in high-performance bulk heterojunction solar cells, including members of the Y family (Y5, Y6, Y11 and Y16), the IT family (IT-4F, IT-M, IT-DM, ITIC and IDIC) and the xBR family (IDFBR and IDTBR). All molecular structures and chemical formulae are shown in Supplementary Fig. 22 (Supplementary Figs. 23 and 24 show the light and dark *JV* curves (where *J* is current density and *V* is voltage) and the device performance is summarized in Supplementary Table 1). Figure 3a shows the monochromatic electric-field-dependent IQE for all of the materials studied; all follow a similar sigmoidal trend (corresponding EQEs are shown in Supplementary Figs. 25 and 26 and full EQE and IQE spectra are shown in Supplementary Figs. 27 and 28). However, the Y-family NFAs tend to produce charge at lower applied electric fields than the IT-family NFAs, which, in turn, outperform the xBR family (Fig. 3b). Only two of the measured materials demonstrated IQEs greater than 2% at zero electric field, and both are members of the Y family (Y11 and Y16).

## Offset dependence of charge generation at planar heterojunctions

**Fig. 4:**
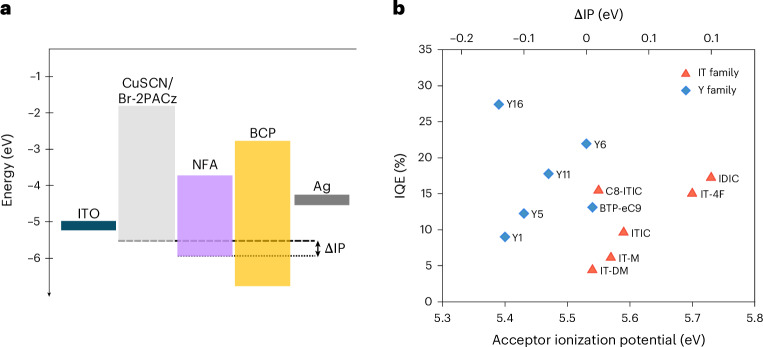
Photocurrent IQE in planar-heterojunction devices with different molecular acceptors. **a**, Energy-level alignment of the layers in the device stack used for these measurements for a generic NFA. **b**, Short-circuit IQE of bilayer devices versus the acceptor ionization potential (bottom *x* axis) and ΔIP with CuSCN (top *x* axis). The chemical structures and formulae of BTP-eC9 (a Y-family NFA) and C8-ITIC (an IT-family NFA) are shown in Supplementary Fig. 22.

We now investigate the performance of these acceptor families in generating photocurrent at donor–acceptor heterojunctions with different energy offsets by measuring the zero-bias IQE of planar-heterojunction devices that contain copper thiocyanate (CuSCN) as the donor (see Fig. 4a for the full device stack and Supplementary Fig. 29 for the *JV* curves). This device structure minimizes microstructural variations compared with a bulk heterojunction, allowing us to better isolate the impact of interfacial energetics, which are known to control the CGE across the donor–acceptor interface^1^. The insolubility of CuSCN in most organic solvents means that a well-defined planar heterojunction can be formed at the CuSCN–acceptor interface, and its wide (>3.4 eV) bandgap ensures that all photoinduced charge generation originates from photon absorption in the acceptor, simplifying analysis using the theoretical framework introduced above^62^. A self-assembled monolayer (Br-2PACz) on top of the CuSCN improved wettability and ensured comparable NFA film formation in single-component and bilayer devices.

Planar-heterojunction IQEs are shown in Fig. 4b, where we plot the IQE at short circuit versus both the IP offset at the CuSCN–acceptor interface (ΔIP = IP_CuSCN_ – IP_NFA_) (top *x* axis) and the IP of the acceptors (bottom *x* axis) (full IQE and EQE spectra are shown in Supplementary Figs. 30–32 and measured IP values are given in Supplementary Table 2 and Supplementary Fig. 33). Bilayers made using IDTBR and IDFBR showed negligible charge generation, probably due to the shallow HOMO relative to CuSCN, and so the xBR family is not represented in this dataset^63–65^. For IT-family devices, we observe a positive correlation of ΔIP with IQE, demonstrating that a less positive energetic offset reduces the efficiency of charge generation. The higher IQE of the C8-ITIC device, compared with that of ITIC, is consistent with its superior performance in bulk heterojunction devices, which was previously attributed to enhanced crystallinity^66^. The Y-family devices show a similar trend, although Y16 is a notable outlier as it achieves the highest IQE despite having the least positive value of ΔIP. Overall, the data indicate that Y-family devices achieve IQEs comparable to the IT family but at smaller energetic offsets. This relative ease of charge generation reflects the better performance of the Y family in single-component devices (Fig. 3). Our findings align with previous reports of higher maximum EQEs for polymer:Y6 bulk heterojunction devices than those using ITIC blended with the same polymer^12,67–69^.

## Discussion

**Fig. 5:**
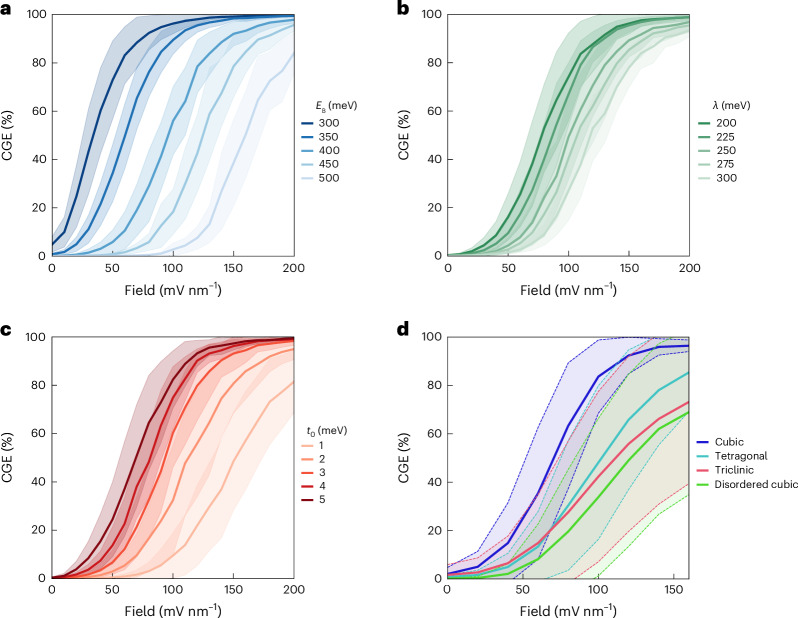
Simulations of CGE of single-component devices as a function of electric field for varying material parameters. **a**–**c**, CGE as a function of the applied electric field for single-component systems simulated using the model described in Methods and Supplementary Information. These simulations used a 10 × 10 square lattice and show the effect of varying *E*_B_ (**a**), *λ* (**b**) and *t*_0_ (**c**). For each parameter value, the shaded intervals indicate the standard deviation from 20 simulations with different realizations of the static disorder and the solid lines indicate the mean value. When not being varied, *E*_B_, *λ* and *t*_0_ take the values stated in Supplementary Table 3. **d**, CGE versus the applied electric field for three-dimensional single-crystal lattices with different symmetries. Details of how the simulations were adapted for crystalline lattices are provided in Supplementary Note 1, Section 1.6. Shaded regions bounded by dashed lines indicate the standard deviation from simulations using 17 different directions of the applied electric field, each of which had 5 realizations of the static disorder (with additional 5 realizations of the geometric disorder in the case of the cubic lattice with geometric disorder), and solid lines indicate the mean value.

To rationalize the experimental observations, we apply the modelling framework described above to explore the impact of the materials’ molecular parameters or packing motifs on charge generation in single materials and heterojunctions. Figure 5a–c shows the modelled CGE versus electric field for varying values of three parameters: *E*_B_, the exciton reorganization energy *λ* (which influences the exciton lifetime; Supplementary Fig. 34) and the nearest-neighbour electronic coupling *t*_0_ (which influences state delocalization; Supplementary Fig. 35). We use values of *t*_0_ that are small compared with calculated NFA electronic couplings to avoid eigenstate delocalization being constrained by the lattice size (Supplementary Note 1, Section 1.3.2). The effects of the varying excitonic coupling, optical gap, polaron reorganization energy and static disorder are shown in Supplementary Figs. 36–39 (the effect of the static dielectric constant is captured in how we vary *E*_B_; Supplementary Note 1, Section 1.1). All parameters other than the one being varied are held constant, with values provided in Supplementary Table 3. Figure 5a–c shows clearly that, at a fixed electric field, the modelled charge generation is higher for smaller *E*_B_, smaller *λ* and larger *t*_0_. Moreover, the simulations reproduce the sigmoidal shape of the measured CGE curves in Fig. 3, with changes in the input parameters varying the ‘turn on’ electric field in a way that resembles the experimental data.

We compare the trends predicted by our model to trends in the relevant parameters reported in literature (Supplementary Table 4). Although *E*_B_ is challenging to measure, recent low-energy inverse photoemission spectroscopy measurements suggest comparable values for IT- and Y-family films, with xBR-family films having larger values^70,71^. Our model would predict a higher turn on electric field for xBR-family materials with their higher *E*_B_, consistent with the observed behaviour (Fig. 5a). A clear trend is also seen in reported values of *λ*, with Y-family materials having the smallest values, followed by the IT family and finally the xBR family^11,24,72–74^. Our model predicts that this would result in the same trend in CGE that is observed experimentally (Fig. 5b). Finally, calculated nearest-neighbour electronic couplings are larger in Y-family crystals than in IT-family crystals, with intermediate values for *o*-IDTBR crystals^23,24,72,75,76^. This is consistent with the observed trend in CGE of IT- and Y-family devices (Figs. 3a and 5c); the low CGE observed experimentally for *o*-IDTBR can be explained by its low crystallinity and the effect of disorder on its electronic couplings^76^. The low crystallinity of *o*-IDTBR may also increase the material’s static disorder and negatively impact CGE, as shown in Supplementary Fig. 38.

Recognizing that anisotropy in intermolecular electronic and excitonic couplings will influence charge generation in real crystalline acceptors, we adapted our model for lattices with different crystal structures, including simple cubic, tetragonal and triclinic, while conserving the mean intermolecular couplings (Supplementary Note 1, Section 1.6). Figure 5d shows that more isotropic intermolecular interactions lead to a higher CGE at low electric field, whereas for the cubic lattice, disorder in electronic and excitonic couplings limits excited-state delocalization and reduces the CGE at low electric fields (Supplementary Fig. 40). These results are consistent with experimental reports^7^ that the photocurrent is affected by small changes in the anisotropy of the acceptor’s unit cell.

**Fig. 6:**
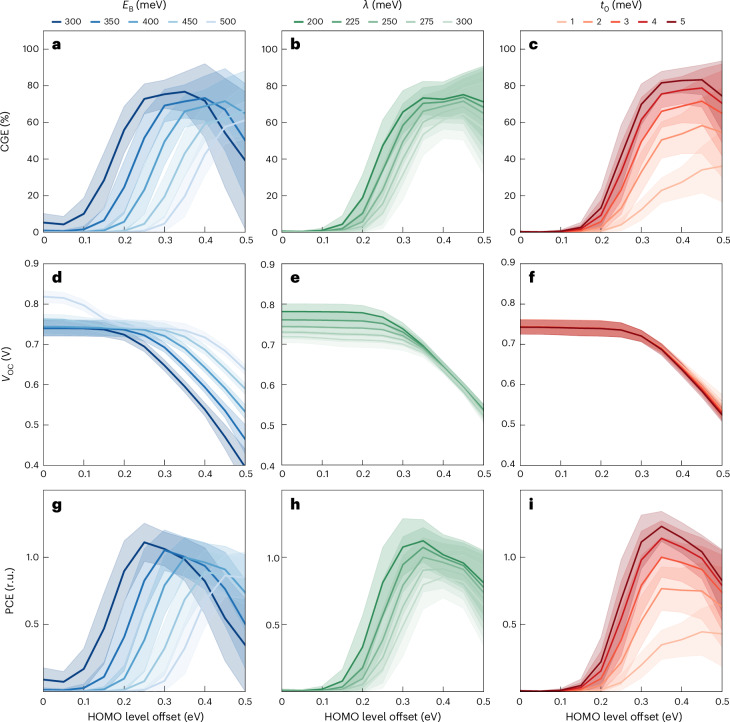
Simulations of the CGE and photovoltaic device behaviour of planar heterojunctions as a function of ΔIP for varying material parameters. **a**–**i**, CGE (**a**–**c**), open-circuit voltage (*V*_OC_) (**d**–**f**) and PCE (**g**–**i**; given in relative units (r.u.) which are defined relative to the maximum PCE achieved by the default parameter set given in Supplementary Table 3) as a function of the HOMO level offset for bilayer devices simulated assuming ideal-diode behaviour, as described in Supplementary Note 1, Section 1.5. Each column corresponds to variation in a different parameter: *E*_B_ (**a**,**d**,**g**), *λ* (**b**,**e**,**h**) and *t*_0_ (**c**,**f**,**i**). When not being varied, each parameter takes the values stated in Supplementary Table 3. In all panels, the shaded intervals indicate the standard deviation from 20 simulations with different realizations of the static disorder and the solid lines indicate the mean value.

To test whether the electric field dependence of charge generation in single-component devices correlates with the CGE in low-offset heterojunctions, we simulated the CGE as a function of ΔIP in planar heterojunctions while varying the parameters investigated above (additional parameters shown in Supplementary Figs. 36–39). Figure 6a–c shows that the same parameters that assist charge generation at low applied biases in single-component systems also facilitate charge generation at low offset in planar heterojunctions. The properties of the common wide-gap donor layer are not varied (although we note that donor electronic coupling can modulate the effect of *t*_0_ on the CGE; Supplementary Fig. 41). Thus, the model predicts that early turn on of electric-field-dependent charge generation is correlated positively with charge generation in low-offset heterojunctions.

The experimental data demonstrate clear differences between material families, with Y-family acceptor-based planar heterojunctions delivering IQEs comparable to or greater than those with IT-family acceptors at less positive ΔIP values (Fig. 4), while also generating higher IQE at low applied electric fields in single-component devices (Fig. 3b). However, the best single-component device materials do not necessarily lead to the best planar heterojunctions. Instead, the heterojunction IQE is better correlated with the device’s ΔIP, illustrating the importance of interfacial energetics. Therefore, while a given acceptor’s electric-field-dependent charge generation cannot safely predict the heterojunction IQE, comparisons between different material families may be indicative of their relative performance in low-offset heterojunctions.

In recent computational work, Balzer and Kassal demonstrated that excited-state delocalization enhances charge generation in low-offset heterojunctions via charges generated in neat material domains^58^. We use our model to explore this relationship and find that the CGE of the lowest-offset heterojunction is comparable to the CGE of the corresponding single-component device at zero electric field, which is large only for low acceptor *E*_B_ (Supplementary Fig. 42). That is, within our model, the contribution of charges generated in the neat material domains to a heterojunction’s CGE does not exceed the CGE of the neat material on its own. As our single-component devices show low IQE at short circuit (≤7%, compared with >80% at high electric field), this suggests that most charge generation in these Y-family heterojunctions occurs across the donor–acceptor interface, as argued elsewhere^20,22,24^. Y16 seems to be an exception to this prediction of our model, achieving an anomalously high IQE in a low-offset heterojunction.

We then apply the model to explore whether exciton dissociation or CT state separation limits CGE in low-offset heterojunctions and low-offset single-component systems, a topic that has been debated in the literature^2,12,77,78^. We find that exciton dissociation limits the CGE in both single-component and low-offset heterojunction devices, while CT state dissociation limits the CGE in higher-offset heterojunctions (Supplementary Note 3). In addition, to consider the impact of state delocalization on the findings, we neglect all off-diagonal couplings in the Hamiltonian and calculate transition rates between states using Marcus–Levich–Jortner theory (Supplementary Note 4). The model without delocalization predicts the same trends as our full model, but approximately double the electric field is needed to reach a CGE of 50% in single-component simulations and the maximum CGE yields in planar heterojunctions are reduced. This indicates that state delocalization is critical in enabling efficient charge generation at low applied bias and at small values of the HOMO level offset.

To explore how the studied material properties impact device performance, we simulate *JV* curves for planar heterojunctions assuming ideal-diode behaviour (see Methods and Supplementary Note 1, Section 1.5) and show the resulting *V*_OC_ (Fig. 6d–f) and relative PCE (Fig. 6g–i) as a function of the HOMO level offset, alongside the corresponding CGE. While the trends in the PCE and CGE are similar, the PCE is more sensitive to variations in molecular parameters. For example, at a ΔIP of 0.3 eV (as reported for PM6:Y6 (ref. ^79^)) the planar-heterojunction PCE may double due to a 30% reduction in the exciton reorganization energy or a 70% increase in electronic coupling. Interestingly, a reduction in *E*_B_ merely shifts the maximum CGE towards less positive HOMO level offsets. This is consistent with the findings of Pranav et al.^2^, who demonstrated that, for given material parameters, the heterojunction CGE depends only on the difference in LE and CT state energies (Supplementary Fig. 43). However, the maximum PCE decreases as *E*_B_ increases (Fig. 6g), because a larger *E*_B_ increases the offset needed to optimize the CGE, thus limiting *V*_OC_ (Fig. 6d). This suggests that, apart from targeting low *E*_B_, materials development should focus on improving other molecular parameters (by reducing the exciton reorganization energy and site energy disorder) and intermolecular parameters (by increasing the magnitude and isotropy of electronic coupling) to improve charge generation and performance in low-offset devices.

## Outlook

Using a new model for charge generation in molecular assemblies that accounts for excited-state delocalization, we have been able to explain observed trends in the charge photogeneration efficiency of different families of small-molecule acceptors in terms of their intramolecular and intermolecular parameters. When compared with IT-family acceptors, Y-family acceptors generate photocurrent at lower electric fields in single domains, and at smaller HOMO level offsets in heterojunctions. Both observations could be explained by superior material parameters, notably lower exciton reorganization energies and larger electronic coupling. A model that could account for delocalized excited states was essential to explain the observed behaviour. Isotropy in molecular packing is also beneficial, helping to explain the importance of crystal packing in achieving a high PCE. Finally, we investigate whether facile charge generation in single-component systems leads to efficient charge generation in planar heterojunctions under short-circuit conditions. While our simulations and experiments agree that the same conditions favour charge generation in both situations, the model shows that there is typically only a small contribution from free charges generated within the acceptor domains to the overall photocurrent generated in a donor–acceptor heterojunction.

## Methods

### Simulation methods

#### Model description

Within our model, the molecular system is represented in a coarse-grained manner by a lattice of molecular centres. The full Hamiltonian is given in Supplementary Note 1, Section 1.1, and we summarize the most important details here. The excited states of the system are described using a Frenkel Hamiltonian that is restricted to a two-particle basis set. This basis set includes both localized excitons (the electron and hole on the same site, |*k, k*〉) and electron–hole pairs (|*i, j*〉, where the first and second indices indicate the sites occupied by electron and hole, respectively), defined using a single HOMO and single LUMO energy for each site (designated *E*_HOMO_ and *E*_LUMO_, respectively). Neat domains and donor–acceptor heterojunctions can be modelled by assigning different HOMO and LUMO energies to different regions of space. The eigenstates of this system are singly excited states, expressed as a linear combination of basis elements. The exciton binding energy, *E*_B_, is defined as the difference between the input singlet exciton energy and the electronic bandgap of the relevant lattice site, *E*_g,el_. This latter quantity is defined as the difference in the HOMO and LUMO energies of the site (that is, the electronic bandgap). Alternatively, *E*_B_ can be expressed in terms of the direct (*J*_0_) and indirect (*K*_0_) Coulomb exchange integrals via *E*_B_ = 2*K*_0_ – *J*_0_. We use values for *E*_B_ that ensure that the energy of the nearest-neighbour CT basis state lies above that of the local exciton. We also include an uncorrelated, Gaussian disorder (called static disorder), *σ*, in the pair energies of our basis states to account for the disordered nature of organic semiconductors^80^.

To illustrate the model, we consider the diagonal elements of the electronic Hamiltonian, which represent the energies of the basis states:

1 $$\begin{array}{l}{\hat{{\bf{H}}}}_{{\rm{el}},{\rm{diagonal}}}\\ =\mathop{\sum }\limits_{k}({E}_{{\rm{g}},{\rm{el}}}-{E}_{{\rm{B}}}+\sigma )|k,k\rangle \langle k,k|+\mathop{\sum }\limits_{i\ne j}({E}_{{\rm{g}},{\rm{el}}}+\sigma -J(|{\bf{r}}|)-q{\bf{r}}\cdot {\bf{F}})|i,j\rangle \langle i,j|\end{array}$$

where the first term represents the energy of excitonic basis elements and the second term represents electron–hole pair elements, **r** is the vector connecting the electron–hole pair, *q* is the electronic charge and *J*(|**r**|) is the electrostatic interaction between the pair modelled using the Mataga potential ($J(|{\bf{r}}|)={J}_{0}/(1+\frac{|{\bf{r}}|}{{r}_{0,j}})$) (ref. ^81^). Basis elements are coupled to one another by either a dipole–dipole coupling if both states are excitonic, or an electronic coupling otherwise. Basis states with |**r**| = 0 are defined as having excitonic character, whereas those for which the electrostatic binding energy of the electron–hole pair is less than the thermal energy (that is, *E*_CS_ ≥ *E*_LUMO_ – *E*_HOMO_ – *k*_B_*T*, where *E*_CS_ is the energy cut-off for defining a charge separated state, *k*_B_ is the Boltzmann constant and *T* is temperature) are defined as having CS character. Excitonic basis states can be populated by photoexcitation, while basis states with CS character are coupled to the external circuit, enabling charge extraction. We include the effects of a uniform electric field, **F**, by altering the energies of electron–hole basis elements by amount $\Delta E=q{\bf{r}}\cdot {\bf{F}}$. The energies of excitonic basis states are unchanged by the electric field. Properties of the basis states can be varied to represent the material type (for example, donor or acceptor sites) in heterojunction systems, and crystalline microstructure can be modelled by using appropriate coupling terms for each molecular pair within the crystal structure. The model does not currently account for short-range electrostatic interactions, such as quadrupoles, which could modulate the offset experienced by charge carriers at a heterojunction^1^.

To calculate the CGE of the ensemble of states for a given set of input parameters, we first find the populations of the eigenstates under steady-state conditions, *P*_*α*_, by solving the system’s rate equations; that is:

2 $${G}_{\alpha }+\mathop{\sum }\limits_{\beta }^{\alpha \ne \beta }\left({k}_{\beta \alpha }{P}_{\beta }-{k}_{\alpha \beta }{P}_{\alpha }\right)-{{k}_{\mathrm{rec}}}^{(\alpha )}{P}_{\alpha }-{{k}_{\mathrm{CS}}}^{(\alpha )}{P}_{\alpha }=0$$

where *G*_*α*_ is the rate of generation into the eigenstate *α*, *k*_*αβ*_ is the rate constant describing population transfer from eigenstate *α* to eigenstate *β*, *k*_rec_^(*α*)^ is the decay rate of *α* to the ground state and *k*_CS_^(*α*)^ is the charge separation rate of *α*. We calculate the rates of these processes as follows, with full details given in Supplementary Note 1, Section 1.3. Decay rates of the eigenstates to the ground state are calculated using generalized Marcus–Levich–Jortner theory, expressed in terms of inner and outer reorganization energies derived from the spectral density function^11,56^, while their generation and extraction rates are calculated on the basis of the contribution to the eigenstate from basis elements with excitonic and CS character, respectively. Rates of population transfer between eigenstates are calculated using secular Redfield theory and, unless otherwise stated, we assume that the coupling of excitons, electrons and holes to the phonon bath can all be described using the same spectral density.

Once we know the populations of the eigenstates, we calculate the CGE using:

3 $$\mathrm{CGE}=\frac{\displaystyle {\sum }_{\alpha }{{k}_{\mathrm{CS}}}^{(\alpha )}{P}_{\alpha }}{\displaystyle {\sum }_{\alpha }{G}_{\alpha }}$$

By addressing the ensemble of states, the free energetic gradients that drive state dynamics are fully represented and thus our model accounts for entropic, as well as energetic, contributions to the driving force for charge separation.

#### Calculation of free energies

To calculate the free energies of the states shown in Fig. 1a,d, we first used the default parameters listed in Supplementary Table 3 to generate an ensemble of microstates. We then binned the microstates into three pools as follows: eigenstates were defined as excitonic if the expectation value of the electron–hole separation was less than the spacing between lattice sites, charge separated if the expectation value of the eigenstate’s extraction rate was over half the value assigned to CS basis states and charge transfer otherwise. Having defined these three ensembles, the free energy of each was then calculated using the formula:

4 $$\Delta G=-\langle {k}_{{\rm{B}}}T\mathrm{ln}(Z)\rangle ;Z=\mathop{\sum }\limits_{\alpha =1}^{n}\exp \left(\frac{-{E}_{\alpha }}{{k}_{{\rm{B}}}T}\right)$$

where the summation over *n* includes all the states within a given pool and *Z* is the canonical partition function. We note that the use of this formula implies that each pool is at quasi-thermal equilibrium, which may not be the case under short-circuit conditions (see Supplementary Note 3 for a further discussion of this assumption). However, as the figure is largely for illustrative purposes, we have made this approximation for simplicity.

To calculate the value of *r*_eh_ for each state shown in Fig. 1a,d, we used the formula

5 $${r}_{\mathrm{eh}}=\frac{\displaystyle {\sum }_{\alpha =1}^{n}{r}_{\alpha }\exp \left(\frac{-{E}_{\alpha }}{{k}_{{\rm{B}}}T}\right)}{Z}$$

where *r*_*α*_ is the expectation value of the electron–hole separation in eigenstate *α*.

#### Calculation of *JV* curves

To go beyond the CGE and study the impact of the electronic and microstructural properties of the system on device performance across the *JV* curve, we include an effective state representing the collection of free charges (FC) generated from the calculated excited states. This FC state models a collection of generated charges in quasi-thermal equilibrium, from which charges can be extracted or reform excited states. This approach enables the construction of a simplified ideal device model based on the lattice of the system, while neglecting additional loss mechanisms such as those arising from charge transport or extraction (for example, shunt and series resistances).

Incorporating the FC effective state into the system’s rate equation (equation (2)) gives:

6 $${G}_{\alpha }+\mathop{\sum }\limits_{\beta }^{\alpha \ne \beta }\left({k}_{\beta \alpha }{P}_{\beta }-{k}_{\alpha \beta }{P}_{\alpha }\right)-{{k}_{\mathrm{rec}}}^{(\alpha )}{P}_{\alpha }-{{k}_{\mathrm{CS}}}^{(\alpha )}{P}_{\alpha }+{{k}_{\mathrm{rf}}}^{(\alpha )}{P}_{\mathrm{FC}}=0$$

7 $$\mathop{\sum }\limits_{\alpha }({{k}_{{\rm{CS}}}}^{(\alpha )}{P}_{\alpha }-{{k}_{{\rm{rf}}}}^{(\alpha )}{P}_{{\rm{FC}}})-{k}_{{\rm{ext}}}{P}_{{\rm{FC}}}=0$$

The FC state is characterized by a free energy ${{\mathcal{G}}}_{\mathrm{FC}}$. Each state *α* is connected to the FC state by a rate constant *k*_CS_^(*α*)^. The reformation rate back to state *α*, *k*_rf_^(*α*)^, depends on the free-energy difference between state *α* and FC, and is given by ${{k}_{\mathrm{rf}}}^{(\alpha )}=\exp (({{\mathcal{G}}}_{\mathrm{FC}}-{E}_{\alpha })/{k}_{{\rm{B}}}T){{k}_{\mathrm{CS}}}^{(\alpha )}$.

The extraction flux *ϕ*_ext_ from the system is controlled by the extraction rate constant *k*_ext_ and is given by the final term in equation (7):

8 $${{{\phi }}}_{\mathrm{ext}}={k}_{\mathrm{ext}}{P}_{\mathrm{FC}}$$

This extraction rate determines the chemical potential of the FC state, which ultimately defines the operating chemical potential of the system. At low extraction rates, the population of the FC state builds up, resulting in a non-zero chemical potential. On the other hand, when extraction is very fast, the chemical potential approaches zero. The chemical potential is expressed in terms of the population of the FC state under illumination *P*_FC_ and when the system is undisturbed, in the dark, ${P}_{{{\rm{FC}}}_{0}}$

9 $${\mu }_{\mathrm{CS}}=-{k}_{{\rm{B}}}T\,\mathrm{ln}({P}_{\mathrm{FC}}/{P}_{{\mathrm{FC}}_{0}})={qV}$$

We assume that, in the dark, the FC state is thermally populated according to a Boltzmann distribution ${P}_{{\mathrm{FC}}_{0}}=\exp (-{{\mathcal{G}}}_{{\mathrm{FC}}_{0}}/{k}_{{\rm{B}}}T)$.

By solving equations (6) and (7) for different values of *k*_ext_, we obtain current–chemical potential pairs, which define the current–voltage characteristic of the system.

More details of the simulation of the current–potential curves and their characteristics are provided in Supplementary Note 1, Section 1.5.

### Device fabrication

#### Single-component devices

The ITO glass substrates were cleaned by sonication in soapy water, deionized water, acetone and isopropyl alcohol for 10 min each. The cleaned substrates were then UV–ozone treated for 15 min. Br-2PACz was dissolved in ethanol at a concentration of 0.3 mg ml^−1^ and stirred overnight. Then, before spin-casting, the Br-2PACz solution was sonicated for 10 min. In an ambient atmosphere, the Br-2PACz was spin-cast onto the UV–ozone treated substrates in a static fashion at 3,000 r.p.m. for 30 s. Here it is important to note that the spinning was started 10 s after the solution was dropped to allow the monolayer to assemble. The substrates were then placed on a hot plate at 90 °C for 10 min. The substrates were then cooled down to room temperature before another round of spin-casting with Br-2PACz. The spin-caster was set to 7,000 r.p.m. for 30 s, during which time the solution was deposited twice to wash off any excess monolayer chains. The substrates were then placed back on the hot plate at 90 °C for 2 min to evaporate any remaining ethanol. NFA were dissolved in chloroform at a concentration of 20 mg ml^−1^ and stirred for 2 h at 55 °C. The solution was then filtered through a 0.45 μm PTFE filter and spin-cast in nitrogen at 3,000 r.p.m. for 30 s, then placed on a hot plate at 85 °C for 5 min. After placing the samples into an evaporator, an 8 nm layer of BCP was deposited through evaporation, followed by a 120 nm layer of silver.

Electron- and hole-selective interlayers (BCP and Br-2PACz, respectively) were used to prevent either electrode presenting a heterojunction for exciton dissociation. This approach of eliminating exciton dissociation at the contacts is the reason why our device structure delivers lower photocurrents than some single-component NFA devices reported in the literature.

We note here the necessity and difficulty of fabricating single-component devices of extremely high uniformity and quality (that is, without pinholes) to withstand the high reverse biases (up to −18V) investigated. Owing to the difficulty of forming films of sufficient quality, BTP-eC9 and C8-ITIC are not shown in our single-component data.

#### Planar-heterojunction devices

ITO glass substrates were cleaned and UV–ozone treated in the same fashion as the single-component devices. Then, copper(I) thiocyanate (CuSCN) was dissolved in diethyl sulfide (DES) at 30 mg ml^−1^ and stirred for 2 h at 60 °C. The solution was then passed through a 0.45 μm PTFE filter, and statically spin-cast in nitrogen onto the substrates at 3,500 r.p.m. for 45 s. The substrates were subsequently placed on a hot plate at 110 °C for 30 min. Next, the Br-2PACz layer was deposited following the same method as described above (in ambient atmosphere). NFA solutions with concentrations of 10 mg ml−1 for the IT-family materials (IT-DM, IT-M, ITIC, IT-4F and IDIC) and 8 mg ml^−1^ for the Y-family materials (Y6, Y5, Y1, Y11, BTP-eC9 and L8-BO) were made and stirred for 2 h at 40 °C. These solutions were then dynamically spin-cast in nitrogen at 1,500 r.p.m. for 45 s, after which they were placed on a hot plate at 85 °C for 5 min. The samples were then loaded into the evaporator, where an 8 nm layer of BCP and a 120 nm layer of silver were deposited through evaporation. After completion, the samples were left overnight under high vacuum (<5 × 10^−7^ mbar) to draw out as much DES from the CuSCN layer as possible.

### Device characterization

#### *JV* measurements

*JV* measurements (in the dark and at AM 1.5) were performed using a Keithley 2400 source measure unit. We used a Newport solar simulator (Ozone free Xenon Arc Lamp, 300 W) to provide the one-Sun (AM 1.5) illumination spectrum at an intensity of 100 mW cm^−2^. A switch-box was used to measure all six pixels in sequence (where one pixel is an active device area of 4.5 mm^2^).

#### EQE

EQE measurements were performed with a Zürich Instruments HF2LI lock-in amplifier together with a chopper at 300 or 700 Hz. A current preamplifier was used to amplify the measured signal (Stanford Research System SR570 or Zürich Instruments HF2TA). A monochromator (Bentham M300) was used to generate the incident monochromatic light beam from a quartz tungsten halogen light source (750–1,100 nm) and a xenon light source (350–750 nm). The light was split at a 50/50 beam splitter with one part relayed to a reference photodiode and the other part focused by an apochromatic objective on a pixel at the sample. A switch-box was used to select the current generated at the illuminated pixel. The EQE was calculated based on the current measured at the reference photodiode and at the sample, respectively. For bias-dependent EQE measurements, a Keithley 2400 source measure unit was used to provide the applied bias.

#### Refractive indices and IQE

The refractive indices of all materials were measured by variable-angle spectroscopic ellipsometry using a SOPRALAB GES5E rotating polarizer ellipsometer with a xenon lamp as a light source and a charge-coupled device (CCD) detector to record optical spectra from 1.2–5.6 eV. Three incidence angles were typically recorded for each sample, varying between 55° and 75°. Light propagation through the device stack was modelled using transfer matrix calculations^82^ using the optical data measured from ellipsometry and device thicknesses measured using a Dektak profilometer (Bruker DektakXT). IQEs were calculated using the measured EQE and the modelled absorption from transfer matrix modelling.

#### PL

For the photoexcitation, we used a 485 nm pulsed diode laser (PicoQuant) with an 80 MHz repetition rate. The excitation power used was 0.4 mW. The resulting luminescence was recorded with a Shamrock 303 spectrograph combined with an iDUS InGaAs array detector (Andor SR 303i-B) cooled to −75 °C. The recorded spectra were background-corrected and normalized to the detector sensitivity, which was determined through calibration with a halogen lamp. A Keithley 236 source measure unit and a switch-box were connected to the device to apply bias to the selected pixel. To determine the PL versus bias at a fixed wavelength, the Shamrock 303 spectrograph combined with a PMT detector (Hamamatsu H10330A-45 or H11706-20 depending on the wavelength range (near-infrared or visible, respectively)) was used. The signal was measured via a lock-in detection scheme (Stanford Research System SR830 DSP) with a chopper operating at 250 Hz.

#### Tr-PL

For the photoexcitation, we used a 485 nm pulsed diode laser (PicoQuant) with 40 MHz repetition rate and an estimated pulse length of ~200 ps. The excitation power was 0.5 mW. The PL was detected via a PMT detector (Hamamatsu H10330A-45 or H11706-20 depending on the wavelength range (near-infrared or visible, respectively)). PL decay traces were recorded with a time-correlated single-photon counting system (Becker-Hickl SPC-130-EM). For bias-dependent photoluminescence measurements, a Keithley 236 source measure unit and a switch-box were connected to the device to apply bias to the selected pixel. To extract exciton lifetimes from the measured PL decay traces, a single exponential decay convoluted with the instrument response function was fitted to the data from single-component devices of Y5 and Y6. The highest applied bias (−18 V) decay trace was used as instrument response function. For single-component devices of ITIC and IT-4F, the lifetimes were shorter and could not be reliably extracted from the fit. The trend of increasingly faster decay was instead captured by fitting only the first nanosecond of decay with a single exponential.

#### Ionization energy measurements

The ionization potential of all materials was measured using air photoemission spectroscopy using the KPTechnology Ltd APS02 system, in which the material is illuminated with a tunable UV lamp and a gold tip placed close to the surface collects the emitted electrons (photo-electron detector). The measurements were performed under air (ambient pressure).

#### Light-intensity-dependent measurements

Light-dependent EQE measurements on planar-heterojunction and single-component devices were conducted by adding a background bias light provided by an external LED ring. The light intensity from the LED ring was set in terms of Suns by using a calibrated photodiode.

Light-dependent PL measurements were performed by varying the excitation intensity coming from the 485 nm pulsed diode laser.

## Online content

Any methods, additional references, Nature Portfolio reporting summaries, source data, extended data, supplementary information, acknowledgements, peer review information; details of author contributions and competing interests; and statements of data and code availability are available at 10.1038/s41563-026-02509-6.

## Supplementary information

Supplementary Information Supplementary Notes 1–4, Figs. 1–43 and Tables 1–6.

## Data Availability

The data needed to reproduce Figs. 1–6 are available via Zenodo at 10.5281/zenodo.18151704 (ref. ^83^).

## Code availability

A version of the code used in this article is available via GitHub at https://github.com/ImperialCollegeLondon/ReCoDE_Lattice_Hamiltonian.
