# Supplementary material for: Molecular factors controlling charge pair generation in organic photovoltaic materials
Source: Nat Mater. 2026 Feb 27;25(7):1209–18. doi: 10.1038/s41563-026-02509-6 (PMC13323082; doi:10.1038/s41563-026-02509-6)
Supplement: Supplementary file 1 — Supplementary Notes 1–4, Figs. 1–43 and Tables 1–6. [file 41563_2026_2509_MOESM1_ESM.pdf]

nature materials

Supplementary information

https://doi.org/10.1038/s41563-026-02509-6

Molecular factors controlling charge pair generation in organic photovoltaic materials

In the format provided by the authors and unedited

1 SUPPLEMENTARY NOTE ONE: DESCRIPTION OF THE MODEL 2

1.1 HAMILTONIAN 2
1.2 PROPERTIES OF THE EIGENSTATES 6
1.3 CALCULATION OF RATES 7
1.3.1 SECULAR REDFIELD 7
1.3.2 VALIDATION OF RATES CALCULATED USING SECULAR REDFIELD 8
1.3.3 GENERALISED MARCUS-LEVICH-JORTNER 9
1.3.4 GENERATION AND EXTRACTION 11
1.4 CALCULATION OF CHARGE GENERATION EFFICIENCY 12
1.5 CURRENT-POTENTIAL CURVE SIMULATION 12
1.6 LATTICE STRUCTURE 14
1.7 APPLICATION OF THE MODEL TO CALCULATE THE STATES FOR A Y6 CRYSTALLITE 16

2 SUPPLEMENTARY NOTE TWO: CALCULATION OF CHARGE GENERATION EFFICIENCY FROM BIAS DEPENDENT PHOTOLUMINESCENCE MEASUREMENTS . 18

2.1 PROBING CHARGE GENERATION EFFICIENCY USING PHOTOLUMINESCENCE SPECTRA 18
2.2 PROBING CHARGE GENERATION EFFICIENCY USING TIME RESOLVED PHOTOLUMINESCENCE 19

3 SUPPLEMENTARY NOTE THREE: INTERPRETATION OF THE SIMULATION RESULTS USING AN EFFECTIVE THREE-STATE MODEL 20

4 SUPPLEMENTARY NOTE FOUR: RESULTS OF MODEL WITH NO DELOCALISATION 24

5 SUPPLEMENTARY FIGURES 27

6 SUPPLEMENTARY TABLES 49

1 Supplementary Note One: Description of the Model

1.1 Hamiltonian

In our model, the system is represented as a lattice, and the excitation Hamiltonian is constructed in the basis of the lattice sites. The basis elements describe configuration where a hole (*h*) and an electron (*e*) occupy specific sites on a lattice of *N* sites, with all other molecules in their ground state (*g*). When both electron and hole occupy the same site, the corresponding basis element is referred to as excitonic (*x*), whereas if they are located at different sites, the element is a charge transfer (CT) element. Each basis element is described as:

$$|k,k\rangle = |g_1 g_2 \dots x_k \dots g_N\rangle$$
$$|i,j\rangle = |g_1 g_2 \dots h_i \dots e_j \dots g_N\rangle$$

To incorporate interactions with the surrounding thermal bath, we assume that each lattice site is linearly coupled to an independent set of phonon modes which are treated as harmonic oscillators. Thus, we split the Hamiltonian into three terms

$$\hat{H} = \hat{H}_{el} + \hat{H}_{el-ph} + \hat{H}_{ph}$$

where the electronic Hamiltonian is given by $\hat{H}_{el}$, the electron phonon coupling by $\hat{H}_{el-ph}$ and the Hamiltonian of the phonon modes by $\hat{H}_{ph}$. In more detail, the electronic Hamiltonian is

$$\begin{aligned}\hat{H}_{el} = \sum_{k}\big(E_{g,el} - E_B + \sigma\big)|k,k\rangle\langle k,k| \\ + \sum_{i\neq j}\big(E_g + \sigma - J(|\vec{r}|) - q\vec{r}.\vec{F}\big)|i,j\rangle\langle i,j| \\ + \sum_{k\neq k'} d(|\vec{r}|)|k,k\rangle\langle k',k'| + \sum_{i\neq j,j'} t_0^{HOMO}(|\vec{r}|)|i,j\rangle\langle i,j'| + \sum_{i,i'\neq j} t_0^{LUMO}(|\vec{r}|)|i,j\rangle\langle i',j| \\ + \sum_{i\neq k} t_{0,LE-CT}^{HOMO}(|\vec{r}|)(|k,k\rangle\langle i,k| + |i,k\rangle\langle k,k|) + \sum_{j\neq k} t_{0,LE-CT}^{LUMO}(|\vec{r}|)(|k,k\rangle\langle k,j| + |k,j\rangle\langle k,k|)\end{aligned}$$

The first and second terms describe the site energies (ε_{ij}, see **Figure 1h**) of the basis elements in which $E_{g,el}$ is the electronic bandgap of the material, E_B the exciton binding energy, $\vec{r}$ the vector describing the separation of the electron and hole, $J(|\vec{r}|)$ the electrostatic interaction between electron-hole pairs, q the fundamental charge, and $\vec{F}$ the externally applied electric field. To account for the static disorder of organic semiconductors, the site energies of the basis elements are assumed to be normally distributed with σ = 50 meV.[2] The electronic bandgap is calculated as $E_{g,el} = E_{LUMO} - E_{HOMO}$ and, for convenience, we set E_{HOMO} of the acceptor to be zero. The final terms describe the coupling between basis elements where $d(|\vec{r}|)$ is the strength of the dipole-dipole coupling between neighbouring excitons, while $t_0^{HOMO}(|\vec{r}|)$ ($t_0^{LUMO}(|\vec{r}|)$) characterises the electronic coupling between neighbouring holes (electrons) when both the basis states involved in the transition have CT character. $t_{0,LE-CT}^{HOMO}(|\vec{r}|)$ and $t_{0,LE-CT}^{LUMO}(|\vec{r}|)$ perform the same role but describe transitions where either the initial or final basis state is excitonic and the other is a CT state. For simplicity, we assume that these couplings are of the same strength as those involved in CT-to-CT transitions. Comparing this Hamiltonian to that shown in **Figure 1h**, we can see that $d(|\vec{r}|) \equiv V_{ij}$, $t_{0,LE-CT} \equiv T_{i,nm}$ and $t_0 \equiv T_{ij,nm}$. The nomenclature used in **Figure 1h** allows for greater generality which is necessary to describe real crystal structures (see **Sections 1.6-1.7**) since here the coupling between sites is not isotropic.

The electrostatic binding energy of an electron-hole pair can be expressed in terms of the direct, J, and exchange, K, Coulomb integrals. For singlet exciton elements, the exciton binding energy is given by $E_B = J_0 - 2K_0$, where J_0 and K_0 are the molecular direct and exchange integral, respectively. The two interactions decay at different rates as a function of the electron-hole separation: while the direct integral scales as $1/|\vec{r}|$, the exchange integral decays exponentially with electron-hole separation. Consequently, we neglect exchange interactions for CT elements.

To describe the direct Coulomb interaction, we use the Mataga potential,[3] which takes the functional form

$$J(|\vec{r}|) = \frac{J_0}{1 + |\vec{r}|/r_{0,j}}$$

where J_0 and $r_{0,j}$ are the two parameters which characterise the shape of the electrostatic potential and control the energetic offset between charge transfer states and local excitons. This form of the electrostatic energy is applicable to singlet excitons and its functional form is designed to empirically account for the effects of electron correlation. [4] We note that E_B can be tuned by changing either of the parameters K_0 or J_0. In the main text, we have shown results obtained by varying J_0 as changing this parameter has a similar effect to that which would be obtained by varying the material's static dielectric constant when using the Coulomb potential and previous work has related an increase in the static dielectric constant of non-fullerene acceptors to their higher charge generation efficiency.[5]

We approximate the excitonic coupling as a dipole-dipole interaction between molecular excitons. Within the point dipole approximation, the coupling between two excitons is given by [6]

$$d(|\vec{r}|) = \frac{\vec{\mu}_1 \cdot \vec{\mu}_2 - 3(\vec{\mu}_1 \cdot \hat{r})(\vec{\mu}_2 \cdot \hat{r})}{|\vec{r}|^3}$$

where $\vec{\mu}_1$ and $\vec{\mu}_2$ are the transition dipole moments of the molecular excitons at sites 1 and 2, respectively, and $\hat{r} = \vec{r}/|\vec{r}|$ is the unitary vector connecting the two sites. The magnitude of the excitonic coupling depends on both the separation between sites and the relative orientation of their transition dipole moments.

In the case of having a 2D lattice with all transition dipole moments aligned in parallel and pointing in the same direction (in a pure H-aggregate), the point dipole approximation can be further approximated as [3]

$$d(|\vec{r}|) = \frac{d_0}{\left(1 + (|\vec{r}| - a)/r_{0,d}\right)^3}$$

where d_0 and $r_{0,d}$ are the two positive parameters which characterise the strength and screening of the dipole-dipole interaction and a is the spacing between lattice sites. The maximum separation for which coupling between excitonic basis states is included in the Hamiltonian can be specified an input parameter to the simulation. For the results shown in the main text, we considered dipole-dipole interactions between all adjacent lattice sites, including those on the diagonals. Inclusion of sites at a greater separation was found to cause a negligible change in the simulation results due to our choice of damping parameters.

The electronic coupling depends on the wavefunction overlap, which decays exponentially with distance. We approximate this coupling as [3]

$$t^{HOMO}(|\vec{r}|) = t_0^{HOMO} \exp\left(-\frac{|\vec{r}| - a}{r_{0,t}}\right)$$

$$t^{LUMO}(|\vec{r}|) = t_0^{LUMO} \exp\left(-\frac{|\vec{r}| - a}{r_{0,t}}\right)$$

where t_0^{HOMO} and t_0^{LUMO} represent the nearest-neighbour HOMO and LUMO coupling, respectively, and $r_{0,t}$ a damping parameter controlling the spatial decay. Due to the exponential form, coupling is dominated by nearest neighbours. The use of a single exponential decay assumes spherically symmetric orbitals and does not account for potential sign changes in the coupling, which can occur in systems with directional orbital character or more complex wavefunction symmetry.

In simulations of bilayers, the lattice was split in half and half the sites were defined to be acceptor sites and the other half donor sites. Donor and acceptor sites were given different values of E_g, E_B, t_0^{HOMO} and t_0^{LUMO} and excitons on the donor were assumed not to contribute to generation, reflecting the large bandgap of the CuSCN. Furthermore, the electronic coupling between donor and acceptor sites was described by separate parameters, $t_{0,DA}^{LUMO}$and $t_{0,DA}^{HOMO}$. We found that, for our default choice of parameters, changing the values of $t_{0,DA}^{LUMO}$and $t_{0,DA}^{HOMO}$ had little effect on the CGE as a function of HOMO level offset, as we show in **Supplementary Supplementary Figure 1a.** However, we note that there are regions of parameter space where the size of $t_{0,DA}$ would have a significant effect on CGE, for instance if the exciton lifetime were shorter such that the rate of exciton decay competes with the rate of charge transfer across the donor:acceptor interface even at large values of the HOMO level offset (**Supplementary Supplementary Figure 1b**).

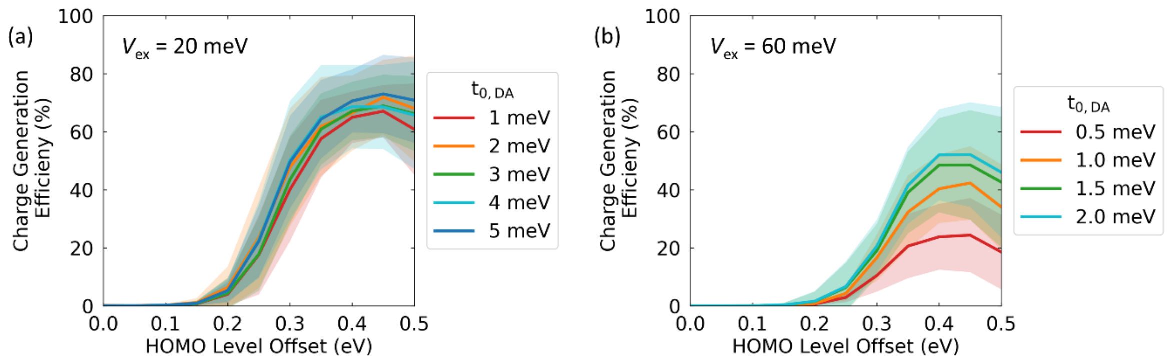

Supplementary Figure 1. Charge generation efficiency versus HOMO level offset for bilayer devices while varying values of the electronic coupling between donor and acceptor sites, $t_{0,DA}$. In all simulations we have assumed that $t_{0,DA}^{LUMO} = t_{0,DA}^{HOMO}$. In (a) we use our default parameter set where the exciton coupling to the ground state, V_{ex}, is 20 meV while, in (b), we set this parameter to 60 meV. The shaded intervals indicate the standard deviation from 20 simulations with different realisations of the static disorder and the solid lines indicate the mean value.

The Hamiltonian of the independent phonon modes is given by

$$\hat{H}_{ph} = \sum_k \sum_\xi \omega_{k,\xi} \ \hat{a}_{k,\xi}^\dagger \hat{a}_{k,\xi}$$

where $\omega_{k,\xi}$ is the characteristic frequency of the ξth mode on site k. Each mode has the creation and annihilation operators $\hat{a}^{\dagger}_{j,\xi}$ and $\hat{a}_{j,\xi}$. We assume that these modes couple linearly to the system, such that

$$\hat{H}_{el-ph} = \left[\sum_{k}\sum_{\xi} \hbar\omega_{k,\xi} g^{ex}_{k,\xi} |k,k\rangle\langle k,k| \left(\hat{a}^{\dagger}_{k,\xi} + \hat{a}_{k,\xi}\right) + \sum_{i\neq j}\sum_{\xi} \hbar\omega_{k,\xi} g^{el}_{i,\xi} |i,j\rangle\langle i,j| \left(\hat{a}^{\dagger}_{i,\xi} + \hat{a}_{i,\xi}\right) + \sum_{i\neq j}\sum_{\xi} \hbar\omega_{k,\xi} g^{hole}_{j,\xi} |i,j\rangle\langle i,j| \left(\hat{a}^{\dagger}_{j,\xi} + \hat{a}_{j,\xi}\right)\right] + H.C.$$

where $g^{x}_{i,\xi}$ is a dimensionless constant describing the coupling of basis states of type x on site i to the ξth mode. To simplify the problem and make it computationally tractable, we avoid summing over multiple phonon modes by replacing the discrete spectral density with a continuous one

$$J^{x}(\omega) = \sum_{\xi} \left(\omega_{\xi} g^{x}_{\xi}\right)^{2} \delta\left(\omega - \omega_{\xi}\right)$$

Additionally, we assume that all types of basis state couple to the phonon modes with the same strength and so drop the superscript x. We define the spectral density function of the basis states using the following functional, previously employed by Renger and Marcus. [7]

$$J(\omega) = A\omega\, exp\left(-\left(\frac{\omega}{\Omega}\right)^{n}\right)$$

The parameter Ω is chosen such that the maximum of $J(\omega)$ occurs at $\hbar\omega = 0.16$ eV, a typical energy for common intra-molecular vibrations in conjugated organic molecules, such as C-C stretching bonds. We use a high value of n ($n = 15$) to ensure that $J(\omega)$ rapidly decays to zero for $\omega > \Omega$ to reflect the fact that organic molecules do not couple strongly to phonon modes with energies greater than ~ 0.2 eV (1600 cm^{-1}). Lastly, A is calculated so that the following normalisation condition is satisfied

$$\frac{\lambda}{\hbar} = \int_{0}^{\infty} d\omega\, \frac{J(\omega)}{\omega}$$

where λ is the total reorganisation energy of the basis state. We show $J(\omega)$ in **Supplementary Figure 2**.

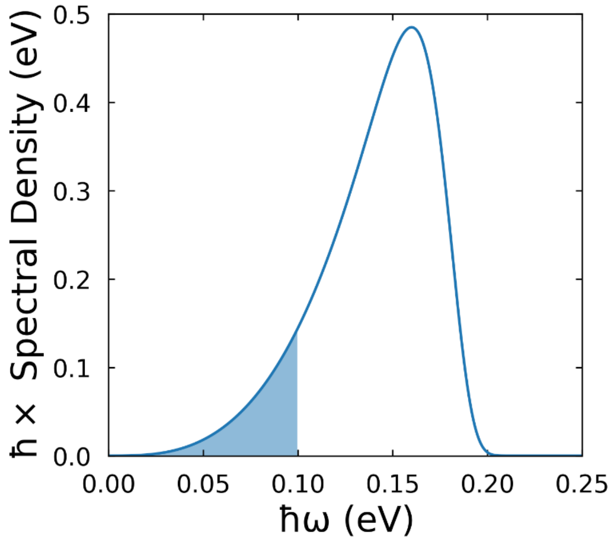

Supplementary Figure 2. Spectral density functional used to calculate rates between states according to the theory described in **Section 1.3.1**. The shaded region represents the modes which are treated as low-frequency modes when calculating decay rates using generalised Marcus-Levich-Jortner (see **Section 1.3.3**).

1.2 Properties of the Eigenstates

Having defined the electronic Hamiltonian of the system, we diagonalise it to find the system's eigenstates for a given set of input parameters

$$\hat{H}_{el}\psi = E\psi$$

where the excited states are solutions to the eigenvalue problem. An excited state α is characterised by an energy E_α and eigenvector

$$\psi^{(\alpha)} = \sum_k c_{kk}{}^{(\alpha)}|k,k\rangle + \sum_{i\neq j} c_{ij}{}^{(\alpha)}|i,j\rangle$$

The electron-hole separation of the state is calculated as

$$r^{(\alpha)} = \langle\psi^{(\alpha)}||\vec{\boldsymbol{r}}_{ij}||\psi^{(\alpha)}\rangle = \sum_{i\neq j}\left|c_{ij}{}^{(\alpha)}\right|^2\left|\vec{\boldsymbol{r}}_{ij}\right|$$

Where the electron-hole separation of the basis element $|i,j\rangle$ is given by $\left|\vec{\boldsymbol{r}}_{ij}\right| = \left|\vec{\boldsymbol{r}}_i - \vec{\boldsymbol{r}}_j\right|$.

The excitonic and charge transfer character of each eigenstate state can be evaluated using the contributions from excitonic$|k,k\rangle$ and charge transfer states $|i,j\rangle$ basis elements, respectively, using the expressions

$$\rho_{ex} = \sum_k\left|c_{kk}{}^{(\alpha)}\right|^2$$

$$\rho_{CT} = \sum_{i\neq j}\left|c_{ij}{}^{(\alpha)}\right|^2$$

The calculated excited states will be delocalised over the whole basis, where the degree of delocalization is given by the inverse participation ratio, which is generally defined as

$$IPR^{(\alpha)} = \sum_i\left|c_i{}^{(\alpha)}\right|^{-4}$$

For a given eigenstate, the inverse participation ratio of the exciton, electron and hole is calculated by first defining a reduced wavefunction for each of these species. For example, in the case of the electron

$$\psi_e^{(\alpha)} = \frac{1}{\sqrt{\sum_{i\neq j} |c_{ij}{}^{(\alpha)}|^2}} \sum_i \sum_{j,i\neq j} c_{ij}{}^{(\alpha)} |i,j\rangle = \frac{1}{\sqrt{\rho_{CT}}} \sum_i \tilde{c_i}{}^{(\alpha)} |i\rangle$$

where the prefactor ensures that the new wavefunction is properly normalised. This wavefunction is then substituted into the definition of the inverse participation ratio to get

$$IPR_e{}^{(\alpha)} = \frac{\rho_{CT}^2}{\sum_i \left|\tilde{c_i}{}^{(\alpha)}\right|^4}$$

The same reasoning can be applied to the exciton and hole species to get

$$IPR_{ex}{}^{(\alpha)} = \frac{\rho_{ex}^2}{\sum_{kk} \left|c_{kk}{}^{(\alpha)}\right|^4}$$

$$IPR_h{}^{(\alpha)} = \frac{\rho_{CT}^2}{\sum_j \left|\tilde{c_j}{}^{(\alpha)}\right|^4}$$

1.3 Calculation of Rates

1.3.1 Secular Redfield

To calculate rates between eigenstates, we use those which come from solving the Redfield Master Equation in the secular approximation (for a derivation see e.g., ref [8]). As the secular approximation decouples diagonal and off-diagonal elements of the density matrix (assuming non-degenerate energy levels), we focus on the equation describing the dissipative dynamics of the populations

$$\left(\frac{\partial \rho_{\alpha\alpha}}{\partial t}\right)_{diss} = -\sum_{\beta\beta} R_{\alpha\alpha,\beta\beta} \rho_{\beta\beta}(t)$$

where $\rho_{\alpha\beta}(t)$ is the reduced density matrix of the system after summing over the bath degrees of freedom and $R_{\alpha\beta,\gamma\delta}$ is the Redfield tensor which, for the system-bath Hamiltonian described by $\widehat{H}_{el-ph}$ in **Section 1.1**, can be written as

$$R_{\alpha\alpha,\beta\beta} = \delta_{\alpha\beta} \sum_{\gamma} k_{\alpha\gamma} - k_{\beta\alpha}$$

in which

$$k_{\alpha\beta} = \left[\sum_k \left|c_{kk}{}^{(\alpha)}\right|^2 \left|c_{kk}{}^{(\beta)}\right|^2 C^{ex}(\omega_{\alpha\beta}) + \sum_{i\neq j} \left|c_{ij}{}^{(\alpha)}\right|^2 \left|c_{ij}{}^{(\beta)}\right|^2 C^{el}(\omega_{\alpha\beta}) + \sum_{j\neq i} \left|c_{ij}{}^{(\alpha)}\right|^2 \left|c_{ij}{}^{(\beta)}\right|^2 C^{hole}(\omega_{\alpha\beta})\right]$$

and where $C^x(\omega)$ is the correlation function for basis states of type x, which is defined in terms of the spectral density function as follows

$$C^x(\omega) = 2\pi\omega^2[1 + n(\omega)][J^x(\omega) - J^x(-\omega)]$$

with n(ω) being the Bose-Einstein occupation function. We note that, when deriving the secular Redfield rates, it is assumed that the phonon bath is thermally occupied. Thus, our model does not account for electronic states which have excess vibrational energy relative that expected in thermal equilibrium.

1.3.2 Validation of Rates Calculated Using Secular Redfield

We note here that secular Redfield theory assumes a weak coupling of the electronic states to the phonon bath. Thus, the energy eigenstates of the system are taken to be those found by diagonalising the electronic Hamiltonian and the rates of transitions between these states are derived by treating the electron-phonon coupling as a second order perturbation. When this framework is applied to typical organic systems, it will tend to overestimate the degree to which states delocalise as polaron formation is neglected. As a result, the calculated transfer rates between states will be too high, especially for those states with a small energy offset.[9,10] To ameliorate this issue, we have limited the size of the electronic couplings we explore to those where the inverse participation ratio of the polaron states is not limited by the lattice size (see **Supplementary Figure 3**). Consequently, the values of the couplings we have used in this work are small compared to values typically calculated for organic crystals.

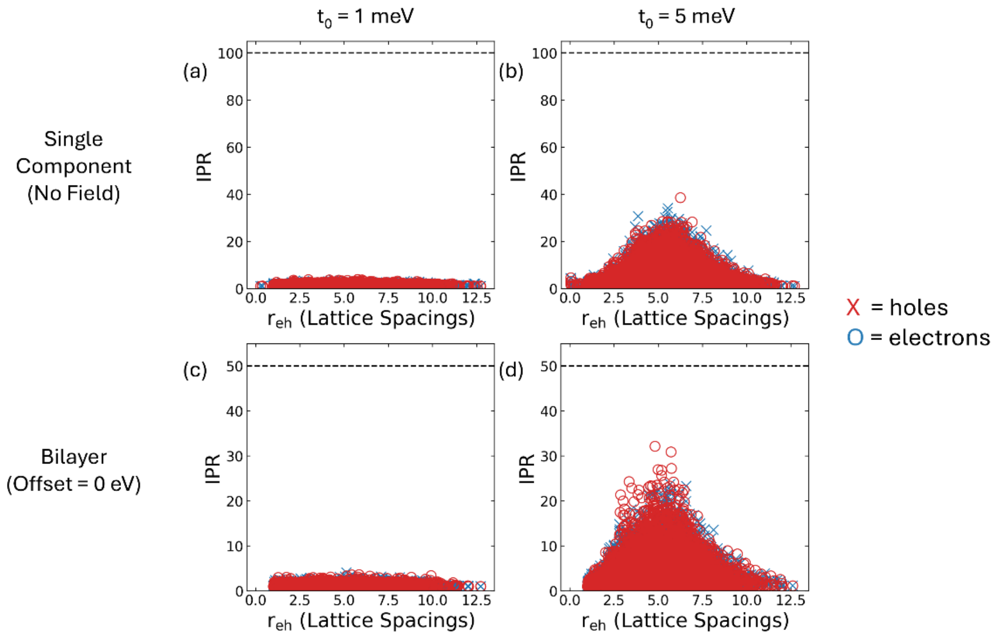

Supplementary Figure 3. Calculated inverse participation ratios (IPR) for electron and hole states in (a-b) single component simulations of a regular lattice containing 10x10 sites with no electric field and (c-d) bilayer simulations with no offset, where half of the lattice is donor and the other half acceptor. In the left-hand coupling, the electronic coupling of the electrons (t_0^{LUMO}) was set to 1 meV and, in the right-hand column, it was set to 5 meV. In single component simulations, we assume $t_0^{LUMO} = t_0^{HOMO}$ and, in bilayer simulations, we fix $t_0^{HOMO} = 3$ meV.

Recent work has overcome this issue by instead using polaron transformed secular Redfield. In this theory, the rates of population transfer are calculated for the eigenstates formed after applying the polaron transformation to the electronic Hamiltonian.[11-13] This transformation has the effect of reducing the size of off-diagonal couplings and thus decreasing delocalisation. For example, in the case of spectral density used herein, an initial electronic coupling of 35 meV between basis states is reduced to a coupling of 3 meV between the basis states of the polaron transformed Hamiltonian, significantly

decreasing the delocalisation of the eigenstates. However, calculating the polaron transformed rates is computationally costly and could only be made tractable for large lattices in refs [11,12] by studying the dynamics using kinetic Monte Carlo, rather than solving Pauli's master equation in the steady state, as is done in this work (see **Section 1.3** , below).

To ensure that the rates we calculate using secular Redfield are not excessively high, we developed code which allows us to calculate rates between states using polaron transformed secular Redfield for a 6×6 lattice and thus directly compare these rates to those calculated using secular Redfield without the polaron transform. The results are shown in **Supplementary Supplementary Figure 4**. Comparison of the rates calculated between eigenstates with an energy separation of ΔE using secular Redfield and polaron transformed secular Redfield. The spectral density is as described in **Section 1.1** and we assumed a reorganisation energy of 250 meV for both excitons and polarons. We used a bare electronic coupling of 35 meV in the polaron transformed secular Redfield calculations, which was reduced to 2.98 meV following renormalisation of the Hamiltonian using the polaron transform. For comparison, we have calculated the non-polaron transformed secular Redfield rates for both these values of the electronic coupling. **,** where we have performed the non-polaron transformed secular Redfield calculations for the value of the electronic coupling obtained before (35 meV) and after (3 meV) applying the polaron transform. It can be seen that using 35 meV in non-polaron transformed secular Redfield leads to a large overestimation of the rates between states in the region where the spectral density is non-zero and would thus lead to a significant overestimation of the CGE. However, when using an electronic coupling of 3 meV, we obtain rates between states which are less than or equal to those calculated using the polaron transformed theory. This observation further motivates our use of electronic couplings in the range 1-5 meV for the simulation results shown in this work.

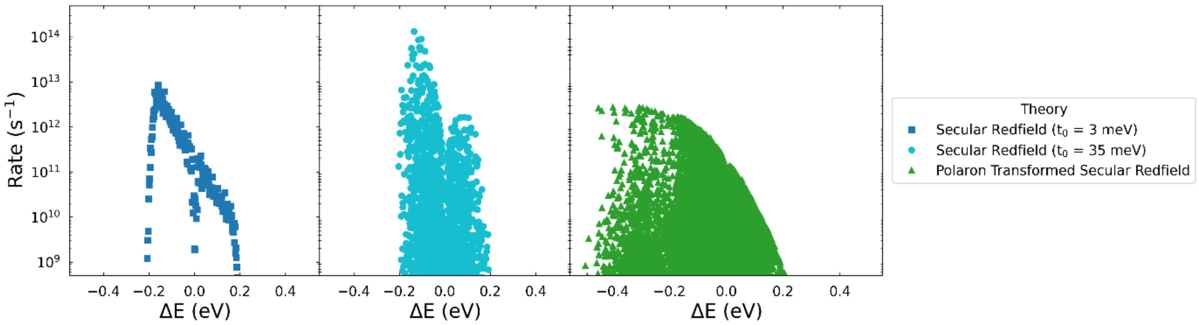

Supplementary Figure 4. Comparison of the rates calculated between eigenstates with an energy separation of ΔE using secular Redfield and polaron transformed secular Redfield. The spectral density is as described in **Section 1.1** and we assumed a reorganisation energy of 250 meV for both excitons and polarons. We used a bare electronic coupling of 35 meV in the polaron transformed secular Redfield calculations, which was reduced to 2.98 meV following renormalisation of the Hamiltonian using the polaron transform. For comparison, we have calculated the non-polaron transformed secular Redfield rates for both these values of the electronic coupling.

Additionally, we note that there is a significant discrepancy between the two theories at large, negative values of ΔE, corresponding to a significant driving force for the state transfer to occur. This is unavoidable as secular Redfield theory only considers single phonon processes, while polaron transformed secular Redfield includes the effects of multi-phonon processes. A consequence of this difference is that some of our bilayer simulations show an 'inverted regime', in which the CGE begins to decrease at large values of the HOMO level offset. We believe that this behaviour is solely due to the limitations of secular Redfield theory, and thus we are not surprised that our experimental data do not exhibit such a trend.

1.3.3 Generalised Marcus-Levich-Jortner

The recombination of the eigenstates to ground state is assumed to be dominated by non-radiative recombination, calculated using a generalised version of Marcus-Levich-Jortner equivalent to generalised Förster or generalised Marcus theories [14,15]

$$k_{rec}{}^{(\alpha)} = \frac{2\pi}{\hbar}\left|V^{(\alpha)}\right|^2 FCWD^{(\alpha)}(\hbar\omega = 0)$$

where the Franck-Condon weighted density of states evaluated at $\hbar\omega = 0$ for a transition from excited state to the ground state is given by

$$FCWD(0) = \frac{1}{\sqrt{4\pi\lambda_l k_B T}} \sum_{w=0}^{\infty}\sum_{t=0}^{\infty} \frac{e^{-S}S^{w-t}t!}{w!}\left[L_t^{w-t}(S)\right]^2 e^{-\{[E+\lambda_l+(w-t)\hbar\Omega]^2/4\lambda_l k_B T\}} \frac{e^{-t\hbar\Omega\beta/k_B T}}{Z_{\hbar\Omega}}$$

It is defined in terms of the energy of the state with respect to ground state E, the reorganization energy of thermally occupied low frequency phonon-modes coupled with the transition λ_l and the Huang-Rhys factor S of an effective high energy mode of energy $\hbar\Omega$ ($S = \lambda_h/\hbar\Omega$). These parameters are extracted from the spectral density function described in **Section 1.1** by defining the low frequency modes as those which have $\hbar\omega < 0.1$ eV (see **Supplementary Supplementary Figure 2**). This cut-off was chosen to give a reasonable split of the total reorganisation energy (250 meV in all simulations unless otherwise specified) into high (202 meV) and low (48 meV) frequency components. $\hbar\Omega$ was chosen to equal 0.16 eV, corresponding to the maximum of the spectral density function, and giving a Huang-Rhys factor of 1.3.

The overlap of the wavefunctions is approximated with the generalized Laguerre polynomials of degree t, $L_t^{w-t}(S)$. The number of ground state and excited states phonon modes w and t can generally be truncated depending on the temperature and energy of the effective mode. Phonon states are considered to be in thermal equilibrium and their occupation in the initial excited state is normalised with the canonical partition function

$$Z_{\hbar\Omega} = \sum_{t=0}^{\infty} e^{-t\hbar\Omega/k_B T}$$

We distinguish between the recombination from excitons and charge transfer elements, where the couplings to the ground are respectively

$$V_{ex}{}^{(\alpha)} = \sum_{kk} c_{kk}{}^{(\alpha)} V_{ex}$$

$$V_{CT}{}^{(\alpha)} = \sum_{ij} c_{ij}{}^{(\alpha)} V_{CT}$$

where V_{ex} is the coupling of excitonic basis elements and V_{CT} is the coupling of CT elements. We consider that only CT elements with electron and hole on adjacent sites have significant coupling to ground state.

Assuming that all basis elements are coupled to the same effective phonon mode, for a delocalized excited state α the low frequency reorganization energy and Huang-Rhys factor for the excitonic and charge transfer recombination is given by

$$\lambda_{l,ex}{}^{(\alpha)} = \frac{\lambda_{l,ex}}{IPR_{ex}}$$

$$S_{ex}{}^{(\alpha)} = \frac{S_{ex}}{IPR_{ex}}$$

$$\lambda_{l,CT}{}^{(\alpha)} = \frac{\lambda_{l,h}}{IPR_h} + \frac{\lambda_{l,e}}{IPR_e}$$

$$S_{CT}{}^{(\alpha)} = \frac{S_h}{IPR_h} + \frac{S_e}{IPR_e}$$

1.3.4 Generation and Extraction

The excited state generation upon illumination is proportional to the square of the state's transition dipole moment. The transition dipole moment of the excited state α is defined as a function of the transition dipole moments of excitonic ($\vec{\mu}_k$) and CT ($\vec{\mu}_{ij}$) elements

$$\vec{\mu}_\alpha = \sum_k c_{kk}\vec{\mu}_{kk} + \sum_{i \neq j} c_{ij}\vec{\mu}_{ij}$$

The generation probably of the excited states is then given by:

$$G^{(\alpha)} = |\vec{\mu}_\alpha|^2$$

In the case of 2D lattices, we simplified the model to reduce the degrees of freedom and the computational costs, as we discuss further in **Section 1.6**. Consequently, we do not explicitly assign transition dipole moments to individual sites in the 2D case. Instead, we assume generation probabilities for excitonic (G_{ex}) and CT elements (G_{CT}), where the generation probability of a given eigenstate is calculated based on exciton and CT character (see **Section 1.2**) as follows:

$$G^{(\alpha)} = \rho_{ex}G_{ex} + \rho_{CT}G_{CT}$$

This simplification implies that the states will only be generated in terms of their density of excitonic basis elements, $\sum_k \left|c_{kk}{}^{(\alpha)}\right|^2$, regardless of their oscillator strength, spectral density or delocalization. Despite the roughness of this approximation, it allows us to describe the absorption process as one in which the excitation comes from excitons with strong transition dipole moment, without adding further complexity to the system. Furthermore, the system's parameters result in fast relaxation of the photogenerated excitons. Consequently, this approximation has minimal implications on the performance of the system since the photogenerated states will relax to lower energy states which have largely excitonic character before recombining or forming a charge transfer state.

Given the low absorption coefficients of CT states, we assume that only excitonic elements absorb light and thus generate excitation into the system. That is, we set $|\vec{\mu}_{kk}| = 1$ and $|\vec{\mu}_{ij}| = 0$, or equivalently, $G_{ex} = 1$ and $G_{CT} = 0$.

We consider basis elements with electron-hole separation greater than a threshold distance r_{thr} generate separate charges with a probability $\theta_{CS} = 1$ and rate constant k_{CS}, otherwise $\theta_{CS} = 0$. The probability that the excited states generate separated charges is given by their density in the CT elements above this threshold, the extraction rate constant of the state being

$$k_{CS}{}^{(\alpha)} = \sum_{ij} \left|c_{ij}{}^{(\alpha)}\right|^2 \theta_{CS,ij} k_{CS}$$

where

$$\theta_{CS,ij} = \begin{cases} 0 & \text{if} \quad |\boldsymbol{r}_j - \boldsymbol{r}_i| < r_{thr} \\ 1 & \text{if} \quad |\boldsymbol{r}_j - \boldsymbol{r}_i| \geq r_{thr} \end{cases}$$

We define r_{thr} as the smaller of the lattice size or the distance at which the Coulomb binding energy of the CT state falls below $k_B T$.

1.4 Calculation of Charge Generation Efficiency

Having calculated the rate constants for excited state generation, relaxation, recombination and extraction, the populations of the states P_α can be obtained by solving Pauli's master equation:

$$\partial/\partial t\, P_\alpha(t) = G_\alpha + \sum_\beta^{\alpha \neq \beta} \left(k_{\beta\alpha} P_\beta(t) - k_{\alpha\beta} P_\alpha(t)\right) - k_{rec}{}^{(\alpha)} P_\alpha(t) - k_{CS}{}^{(\alpha)} P_\alpha(t)$$

At the steady state, $\partial/\partial t\, P_\alpha(t) = 0$, the populations are time independent. The system of equation to solve is then:

$$G_\alpha + \sum_\beta^{\alpha \neq \beta} \left(k_{\beta\alpha} P_\beta - k_{\alpha\beta} P_\alpha\right) - k_{rec}{}^{(\alpha)} P_\alpha - k_{CS}{}^{(\alpha)} P_\alpha = 0$$

Once the steady-state populations P_α are known, we can compute the quantum yields for charge generation efficiency (CGE) and recombination as:

$$CGE = \frac{\sum_\alpha k_{CS}{}^{(\alpha)} P_\alpha}{\sum_\alpha G_\alpha}$$

$$K_{rec} = \frac{\sum_\alpha k_{rec}{}^{(\alpha)} P_\alpha}{\sum_\alpha G_\alpha}$$

1.5 Current-Potential Curve Simulation

To study how electronic and microstructural properties influence device performance, we introduce an effective state representing the collection of free charges (FC) state representing a population of generated charges in quasi-thermal equilibrium. From this FC state, charges can be extracted or reform excited states, from which they may then recombine. This enables simulation of an idealised device model based on the system's lattice, while neglecting additional losses such as charge transport and contact resistances.

Incorporating the FC state into the rate equation yields:

$$G_\alpha + \sum_\beta^{\alpha \neq \beta} \left(k_{\beta\alpha} P_\beta - k_{\alpha\beta} P_\alpha\right) - k_{rec}{}^{(\alpha)} P_\alpha - k_{CS}{}^{(\alpha)} P_\alpha + k_{rf}{}^{(\alpha)} P_{FC} = 0$$

$$\sum_\alpha \left(k_{CS}{}^{(\alpha)} P_\alpha - k_{rf}{}^{(\alpha)} P_{FC}\right) - k_{ext} P_{FC} = 0$$

The FC state is characterized by a free energy $\mathcal{G}_{FC}$. Each state α connects to the FC state via a forward rate constant $k_{CS}{}^{(\alpha)}$, as defined in **Section 1.3.4**, and a reverse rate $k_{rf}{}^{(\alpha)} = \exp((\mathcal{G}_{FC} - E_\alpha)/k_B T)\, k_{CS}{}^{(\alpha)}$.

The extraction current is:

$$\phi_{ext} = k_{ext} \cdot P_{FC}$$

This extraction rate determines the chemical potential of the FC state, setting the system's operating potential (V), which is given by:

$$\mu_{FC} = qV = -k_B T \ln\left(P_{FC}/P_{FC_0}\right)$$

where $P_{FC_0} = \exp\left(-\mathcal{G}_{FC_0}/k_B T\right)$ is the equilibrium population of the FC state in the dark, which we assume to follow a Boltzmann distribution.

By solving the rate equations containing the FC state for different values of k_{ext}, we obtain (ϕ_{ext}, μ_{FC}) pairs that define the current-potential characteristics, that depend on the balance between losses and charge generation, determined by the system's electronic and microstructural properties. For every k_{ext}, the extracted power of the system is:

$$\mathcal{P} = \phi_{ext} \cdot \mu_{FC}$$

At $k_{ext} = 0$, the system is at open-circuit, with maximum potential V_{oc}. As $k_{ext} \to \infty$, the system reaches short-circuit conditions, where all generated charges are extracted. Thus, $\phi_{ext,sc} = J_{sc} = CGE$ and $\mu_{FC} = 0$. We note that taking the limit $k_{ext} \to \infty$ is an approximation which, although valid in the idealised cells considered herein, is unlikely to apply to real organic solar cells due to the relatively low carrier mobilities of organic semiconducting materials.

The current and the operating potential of the system, and thus the extracted power, depends on the competition between charge generation and loss processes in the material, and is therefore determined by the material's properties. For example, **Supplementary Figure 5b** shows the current–voltage curves of a 10×10-site 2D bilayer system with different offsets between the HOMO energy levels of the donor and acceptor. It can be seen how increasing the energy offset enhances charge generation, resulting in a higher J_{sc}, but with the trade-off of a reduced V_{oc}. As discussed in **Figure 6** and the main text, an optimal offset can be found depending on the chemical properties of the material.

The maximum chemical potential of the FC state also depends on the exciton generation rate G_α. Higher generation rates (i.e., higher light intensity) lead to a linear increase in the extracted current, and thus in J_{sc}. Meanwhile, because all transitions are treated as first order, V_{oc} scales logarithmically with G_α (or equivalently the light intensity φ), yielding an ideality factor $n_{id} = 1$, as shown in **Supplementary Figure 5c**:

$$n_{id} = k_B T \frac{d\, V_{oc}}{d \ln(\varphi)}$$

This ideal behaviour reflects the model's neglect of processes such as trap mediated recombination and non-uniform carrier distributions due to space-charge effects. As a result, our simulated current–potential curves represent an upper limit to the photovoltaic performance of the simulated material under idealized conditions. This is further supported by the agreement between the simulated fill factor (FF) and Green's empirical expression for the maximum FF [16] (see **Supplementary Figure 5d**):

$$FF_{emp} = \frac{\gamma_{oc} - \ln(\gamma_{oc} + 0.72)}{\gamma_{oc} + 1}$$

with $\gamma_{oc} = qV_{oc}/n_{id} k_B T$.

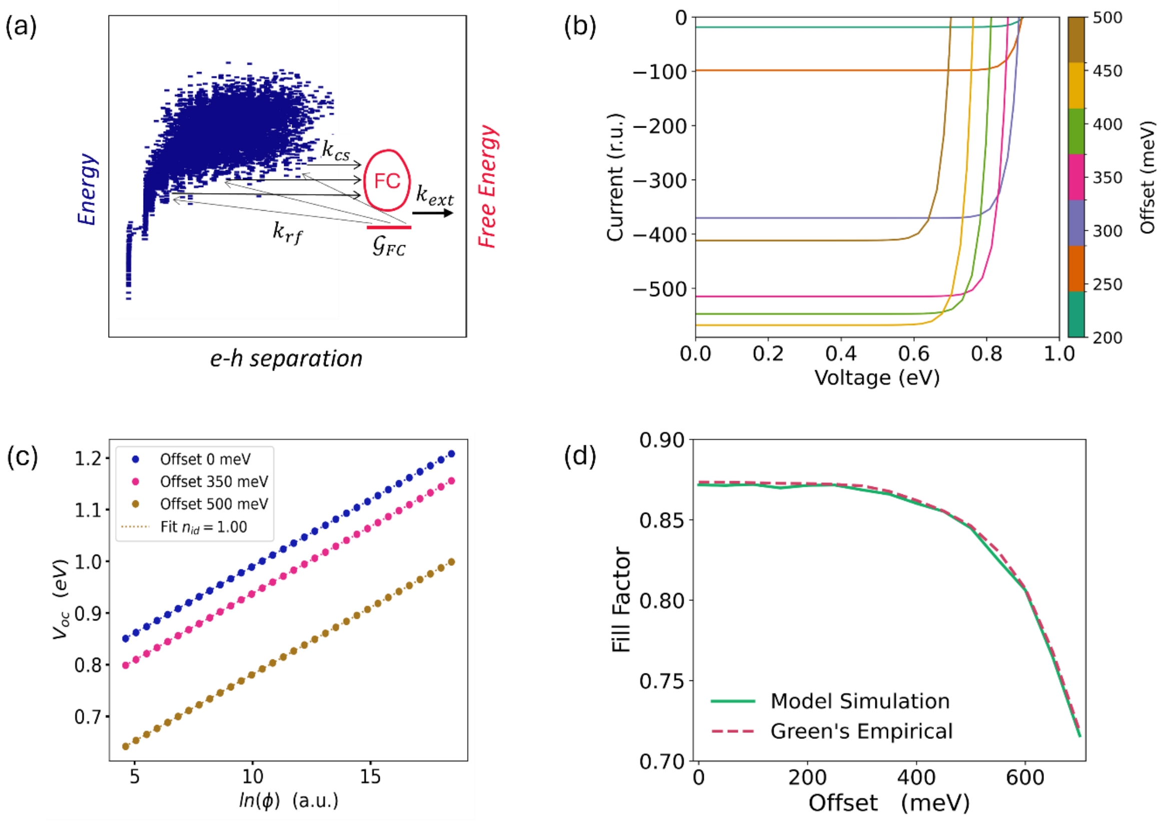

Supplementary Figure 5. (a) Schematic diagram of the calculated excited states for a 2D system with 10x10 sites, connected to an effective FC state. (b) Simulated J–V curves for a 2D planar bilayer system with 10x10 sites under varying energy offsets between donor and acceptor sites, as shown in Figure 6 of the main text. Increasing the offset raises the current but lowers the V_{oc}. (c) Open circuit voltage as a function of light intensity ϕ, proportional to excited state generation $\sum G_\alpha$, for three different bilayer systems, and all showing an ideality factor of 1. (d) Comparison of the simulated fill factor with the empirical fill factor (see main text) for the offset variations shown panel (b).

From the calculated power-potential curves, we determine the power extracted at the maximum power point. To allow a meaningful comparison between systems, we normalise all the extracted powers to the maximum value obtained by the default parameter set given in **Supplementary Table 3**.

1.6 Lattice structure

The model described here can, in principle, be applied to any molecular structure, where the system is coarse grained such that each molecule is represented by a single site. Interactions between these sites are approximated using methods generally applicable to organic conjugated molecules, such as those described in **Section 1.1**. The basis set includes both local excitons and CT elements, leading to a total N^2 basis elements for a lattice of N sites. After computing the N^2 eigenstates, we construct a rate matrix that captures transition rate constants between all unique pairs of eigenstates and to the ground state. This involves calculating $N^2(N^2 - 1)$ inter-state (off-diagonal) rates and N^2 recombination (diagonal) rates, a total of N^4 calculations. Thus, calculating the full rate matrix comes with a high computational cost, which becomes a significant challenge for large systems. At the same time, sufficiently large lattices are necessary to capture the charge generation character of the states, defined here as those with a Coulomb interaction lower than $k_B T$. This presents a trade-off between physical realism and computational feasibility.

Given this trade-off, we adapt the lattice structure depending on the specific aspect of the system we aim to investigate. To study the effects of electronic parameters—such as electronic energy levels, coupling strength, or reorganization energy—we prioritize spatially larger lattices. Thus, in these cases, we use 2D structures with reduced degrees of freedom, enabling significantly larger system sizes to be simulated within manageable computational costs. Specifically, we use a 10×10 superlattice (100 sites), as illustrated in **Supplementary Figure 6a**, with a lattice constant of 10 Å, resulting in a superlattice side length of 100 nm.

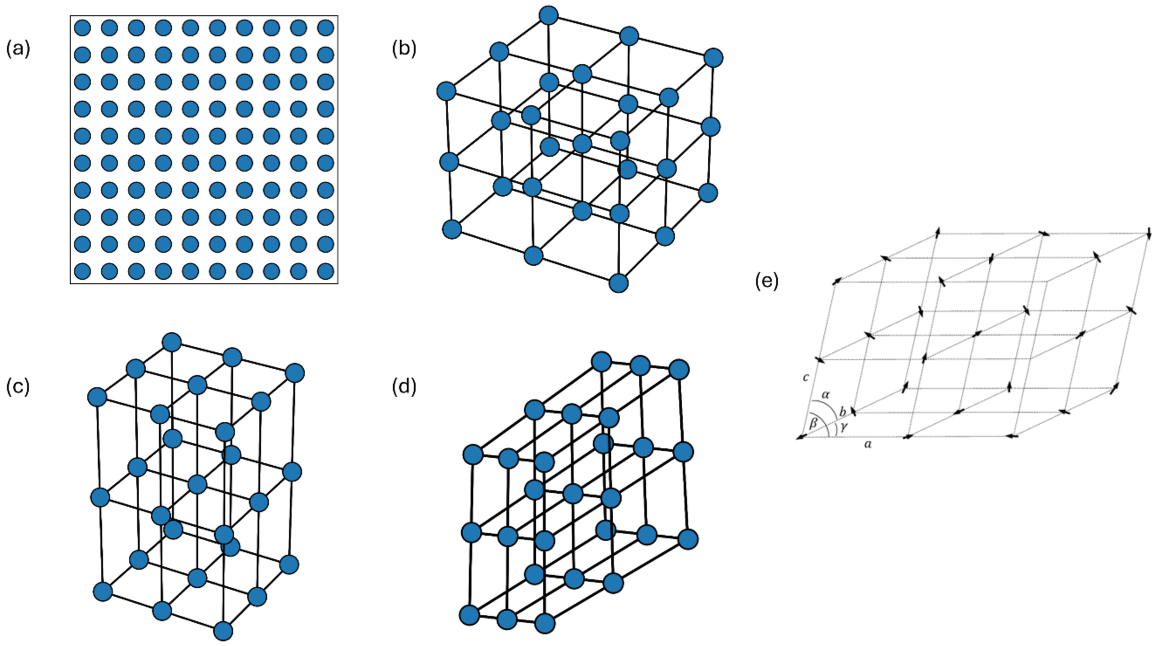

Supplementary Figure 6. Structures of the lattices used in this work. (a) 10x10 2D lattice, (b) simple cubic lattice, (c) tetragonal lattice, (d) triclinic lattice with one molecule per unit cell. (e) illustrates the definition of the lattice's side lengths (a, b, c) and interaxial angles (α, β, γ).

By contrast, to study the effects of structural disorder, we need to use a structure which can better capture the complexity of real molecular systems. Thus, we use 3D lattices with varied unit cells. However, 3D lattices significantly increase computational demands as they increase the number of sites needed to reach a given maximum electron-hole separation and they introduce additional degrees of freedom, such as the orientation of transition dipole moments and the direction of the external field relative to the symmetry axes of the 3D lattice. As a result, the overall lattice size must be constrained.

The unit cell of a periodic 3D structure can be defined by its side lengths (a, b, c) and interaxial angles (α, β, γ), as illustrated in **Supplementary Figure 6e**. In **Figure 5d** of the main text, we compare a simple cubic structure ($a = b = c = 10$ Å; $\alpha = \beta = \gamma = 90°$, **Supplementary Figure 6b**), a tetragonal structure ($a = b = 11$ Å, $c = 8$ Å; $\alpha = \beta = \gamma = 90°$, **Supplementary Figure 6c**), and a triclinic structure ($a = 7$ Å, $b = 9$ Å, $c = 14$ Å; $\alpha = 80°$, $\beta = 75°$, $\gamma = 115°$, **Supplementary Figure 6d**). For all cases we used a superlattice of $5 \times 5 \times 5$ unit cells (125 sites).

To reduce the degrees of freedom in the 3D systems, we assume that all the transition dipole moments are parallel. This approximation does not have a significant impact on the results, as shown in **Supplementary Figure 7a**. Additionally, it enables a more consistent comparison with the 2D case. To ensure that the average coupling remains consistent across different lattice geometries, we scale the nearest neighbour coupling parameter, t_0, such that the average coupling, given by $\sum t_0/(N^2(N^2-1))$, is the same for all structures considered. In the case of the simple cubic crystal structure, we also consider the effect of geometric disorder in the site coordinates. To do this, we displace the lattice sites

from their equilibrium positions by a vector in which each component is chosen at random from a normal distribution with a given standard deviation. To generate the results shown in **Figure 5d**, we used a standard deviation of 1.5 Å. Results for different values of the standard deviation are shown in **Supplementary Figure 7b**.

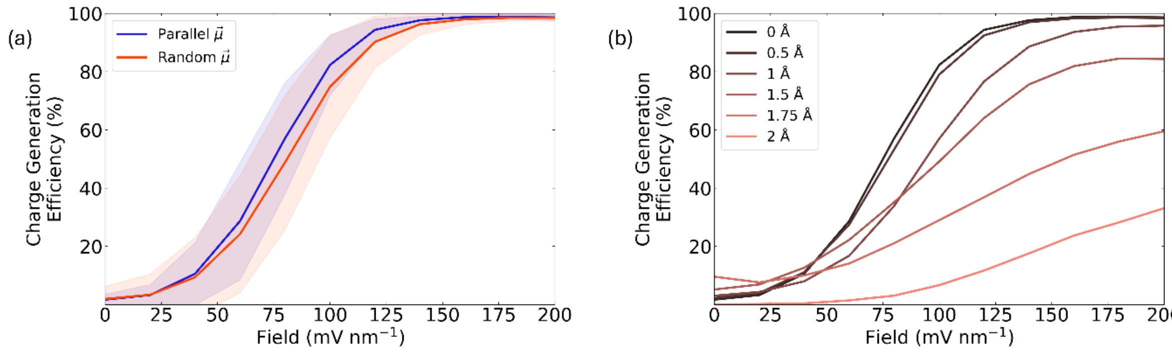

Supplementary Figure 7. (a) Simulated CGE versus field for a 3x3x3 simple cubic lattice in which we either assume a random orientation of the transition dipole moments (random $\vec{\mu}$) or, as done for the results in them main text, we assume that all the transition dipole moments are parallel (parallel $\vec{\mu}$). This choice does not significantly impact the shape of the CGE versus field. (b) Simulated CGE versus field for a 3x3x3 simple cubic lattice in which we have used different values for the standard deviation of the geometric disorder in the site coordinates.

1.7 Application of the Model to Calculate the States for a Y6 Crystallite

We use the Y6 crystal structure provided in **ref.** [1] as an example to illustrate how the Hamiltonian depicted in **Figure 1h** can be parametrized for a specific molecular system and our results are shown in **Supplementary Figure 8**, below. We calculate the off-diagonal coupling terms (V_{ij}, $T_{i,nm}$ and $T_{ij,nm}$) from the molecular geometries in a crystal lattice of 3x3x3 unit cells (108 molecules), see **Figure 1g**. Electronic couplings are computed for every unique molecular pair with an edge-to-edge separation below 15 Å and are found to be negligible for separations greater than 7 Å. These couplings are obtained from HOMO and LUMO orbitals in DFT calculations (B3LYP/6-31g*) on the molecular crystal geometries, using the DIPRO method as described in ref. [17]. Excitonic couplings are evaluated with the TrESP method (ref. [18]), which uses atomic transition charges obtained by fitting the electrostatic potential (ESP) of the TDDFT calculated transition density. We derive transition charges using Multiwfn software (ref. [19]). Site energies of molecular exciton and charge-transfer states between nearest neighbours are taken from ref. [20]. The distance dependence of the electron–hole Coulomb potential is determined from cation and anion atomic charges, obtained by fitting the ESP of molecules in their charged states. The cation and anion atomic charges used in the Coulomb calculations, as well as the transition charges used for excitonic coupling, are derived from an optimized Y6 molecule and then mapped onto the corresponding atoms in the crystal structure. We optimize the molecular geometry with DFT using the B3LYP functional and the 6-311+G(d,p) basis set. Subsequent DFT and TDDFT calculations to obtain cation, anion, and transition densities employ the same functional and basis set.

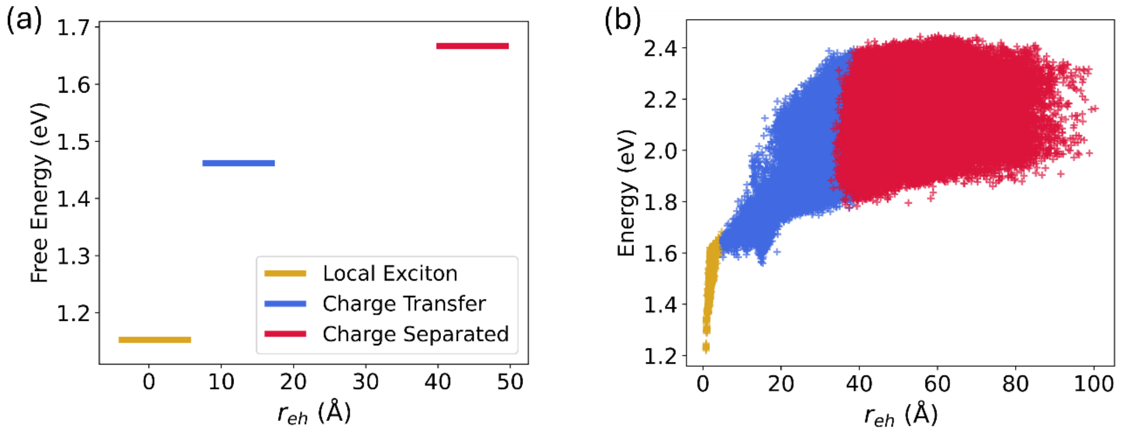

Supplementary Figure 8. (a) Schematic state diagram showing free energy of the local exciton (LE), charge transfer (CT) and charge separated (CS) state as calculated for a 3x3x3 supercell of Y6 (108 molecules) using the crystal structure reported in ref.[1]. (b) Detailed state diagram for a single crystal of Y6 showing the enthalpy of each singly excited state plotted against its average electron-hole separation, r_{eh}. States coloured yellow, blue and red correspond to excitonic, CT and CS states, respectively.

2 Supplementary Note Two: Calculation of Charge Generation Efficiency from Bias Dependent Photoluminescence Measurements

2.1 Probing Charge Generation Efficiency using Photoluminescence Spectra

When measuring photoluminescence (PL), we measure the number of photons emitted from the sample following the radiative decay of the excited state generated by the laser. If we assume that the main emissive state is the exciton and that the measurement is done under steady state conditions, we can relate the photoluminescence intensity to the number of excitons present in the sample as follows:

$$I_{\mathrm{PL}}(V) \propto n_{\mathrm{ex}}(V) \tag{1}$$

Where $I_{\mathrm{PL}}(V)$ is the PL intensity under an applied voltage V and $n_{\mathrm{ex}}(V)$ is the number of excitons in the sample under steady state at voltage V. We then describe the system's kinetics using the following set of rate equations:

$$\frac{dn_{\mathrm{ex}}}{dt} = G - k_{\mathrm{rec}} n_{\mathrm{ex}} - k_{\mathrm{dis}} n_{\mathrm{ex}} \tag{2}$$

$$\frac{dn_{\mathrm{fc}}}{dt} = 2k_{\mathrm{dis}} n_{\mathrm{ex}} - k_{\mathrm{collect}} n_{\mathrm{fc}} \tag{3}$$

Where G is the generation rate per unit volume, n_{ex} is the number density of excitons, k_{rec} is the rate constant of exciton recombination (including both radiative and non-radiative pathways), k_{dis} is the rate constant of exciton dissociation, n_{fc} the number of free charges, and k_{collect} the rate of collection of the free charges. Under steady state conditions we can rewrite the equations in the form:

$$G = k_{\mathrm{rec}} n_{\mathrm{ex}} + k_{\mathrm{dis}} n_{\mathrm{ex}} = k_{\mathrm{rec}} n_{\mathrm{ex}} + k_{\mathrm{collect}} \frac{n_{\mathrm{fc}}}{2} \tag{4}$$

$$2k_{\mathrm{dis}} n_{\mathrm{ex}} = k_{\mathrm{collect}} n_{\mathrm{fc}} \tag{5}$$

We also define the charge generation efficiency as:

$$CGE(V) = \frac{k_{\mathrm{collect}} \frac{n_{\mathrm{fc}}}{2}}{G} = \frac{G - k_{\mathrm{rec}} n_{\mathrm{ex}}}{G} = 1 - \frac{k_{\mathrm{rec}} n_{\mathrm{ex}}(V)}{G} \tag{6}$$

where CGE(V) is the charge generation efficiency at the externally applied voltage V. If we assume that exciton dissociation can be considered negligible under no applied bias, then

$$k_{\mathrm{rec}} n_{\mathrm{ex}}(0) \propto G, \tag{7}$$

which means all the photogenerated excitons recombine. Using this assumption, the charge generation efficiency can be related to the intensity of the PL as

$$CGE(V) = 1 - \frac{I_{\mathrm{PL}}(V)}{I_{\mathrm{PL}}(0)}. \tag{8}$$

2.2 Probing Charge Generation Efficiency using Time Resolved Photoluminescence

We can relate the charge generation efficiency to the measured time constant of the transient photoluminescence decay (τ_{TRPL}) as follows. First, we note that the intensity of the transient photoluminescence (tr-PL) signal is directly proportional to the exciton population

$$I_{\mathrm{TRPL}}(t, V) \propto n_{\mathrm{ex}}(V, t) \tag{9}$$

We then assume that the exciton population decays as follows:

$$n_{\mathrm{ex}}(V, t) \propto \exp\left(-\frac{t}{\tau_{\mathrm{TRPL}}}\right) \tag{10}$$

As for the steady-state PL, we can use equations (2) and (3) to describe the system's kinetics. During the tr-PL decay, there is no generation of excitons, and hence we can combine equations 2 and 10 to find that:

$$\frac{dn_{\mathrm{ex}}}{dt} = -\frac{n_{\mathrm{ex}}(V, t)}{\tau_{\mathrm{TRPL}}(V)} = -k_{\mathrm{dis}}(V) n_{\mathrm{ex}}(V, t) - k_{\mathrm{rec}}(V) n_{\mathrm{ex}}(V, t) \tag{11}$$

If the recombination rate (k_{rec}) is independent of the voltage applied/ electric field, it follows that the rate of exciton dissociation is:

$$k_{\mathrm{dis}}(V) = \frac{1}{\tau_{\mathrm{TRPL}}(V)} - k_{\mathrm{rec}} \,. \tag{12}$$

The charge generation efficiency can be written in terms of rate constants (we consider only the decay of the exciton as the main loss mechanism)

$$CGE(V) = \frac{k_{\mathrm{dis}}(V)}{k_{\mathrm{rec}} + k_{\mathrm{dis}}(V)} = \frac{\frac{1}{\tau_{\mathrm{TRPL}}(V)} - k_{\mathrm{rec}}}{\frac{1}{\tau_{\mathrm{TRPL}}(V)}} \tag{13}$$

The rate of recombination of the exciton (k_{rec}) can be inferred from the results at no applied voltage where the charge generation efficiency is normally very small, in other words:

$$\frac{1}{k_{\mathrm{rec}}} = \tau_{\mathrm{TRPL}}(0) \,. \tag{14}$$

Finally, we can write the charge generation efficiency in the form:

$$CGE(V) = 1 - \frac{\tau_{\mathrm{TRPL}}(V)}{\tau_{\mathrm{TRPL}}(0)} . \tag{15}$$

3 Supplementary Note Three: Interpretation of the Simulation Results Using an Effective Three-State Model

This section details how the results of the simulations can be expressed in terms of three excited states, namely a single effective local exciton (LE), charge transfer (CT) and charge separated (CS) state. This allows the key limitations and loss pathways during photocurrent generation to be quantified in terms of these three classes of state and thus relate the results of the simulations to other findings in the literature.

Method

First, we use the steady state populations of the eigenstates, P_α, (see **Section 1.4**) to find the system's grand canonical partition function and the chemical potential, μ_α, of each eigenstate using the formulae

$$\mathcal{Z} = \sum_{\alpha} \exp\left(\frac{\mu_\alpha - E_\alpha}{k_B T}\right) = \sum_{\alpha} P_\alpha$$

$$\mu_\alpha = E_\alpha + k_B T ln(P_\alpha)$$

In which $\mathcal{Z}$ is the grand canonical partition function of the system and E_α is the energy of the eigenstate α. The chemical potential is calculated under the assumption that all the microstates share a common chemical potential in the dark and thus their dark equilibrium populations follow a Boltzmann distribution.

Then, to evaluate our simulation results within the framework of a three-state model, we binned the eigenstates into three macrostates as follows: eigenstates were defined as excitonic (LE) if the expectation value of the electron-hole separation was less than the spacing between lattice sites, charge separated (CS) if the expectation value of the eigenstate's extraction rate was over half the value assigned to CS basis states and charge transfer (CT) otherwise. Having binned the states in this way, we could then calculate the rate of population transfer from macrostate i to macrostate j as follows

$$R_{i,j} = \sum_{\alpha_i} \sum_{\beta_j} k_{\alpha_i \beta_j} P_{\alpha_i}$$

where $k_{\alpha\beta}$ is the transfer rate calculated between eigenstates α and β in the full model (see **Section 1.3.1**) and here a summation over α_i means to sum over all the eigenstates which have been categorised as belonging to the ith macrostate. Similarly, the rates of extraction (from CS states) and decay (from LE and CT states) can be calculated via

$$R_{out} = \sum_{\alpha_{CS}} {k_{CS}}^{(\alpha_{CS})} P_{\alpha_{CS}}$$

$$R_{rec,i} = \sum_{\alpha_i} {k_{rec,i}}^{(\alpha_i)} P_{\alpha_i}$$

where i can refer to either the LE or CT macrostate (see **Section 1.3.3** for the definition of the ${k_{rec}}^{(\alpha)}$ and **Section 1.3.4** for the definition of ${k_{CS}}^{(\alpha)}$). Effective rate constants can be defined for each process by dividing the rate of the process by the relevant population. For example,

$$K_{LE,CT} = \frac{R_{LE,CT}}{\sum_{\alpha_{LE}} P_{\alpha_{LE}}} = \frac{R_{LE,CT}}{\mathcal{Z}_{LE}}$$

$$K_{rec,LE} = \frac{R_{rec,LE}}{\sum_{\alpha_{LE}} P_{\alpha_{LE}}} = \frac{R_{rec,LE}}{\mathcal{Z}_{LE}}$$

etc. in which $\mathcal{Z}_i$ is the grand canonical partition function for the ith macrostate. Lastly, we can define a net flux between two macrostates as follows

$$F_{i,j} = R_{i,j} - R_{j,i}$$

The treatment above finds the populations of and transfer rates between the effective macrostates without any assumption of thermalisation within each macrostate. The rates obtained in this manner can be contrasted with those which we would obtain if we assumed that microstates within each macrostate were at quasi-thermal equilibrium, as is typically done in effective three-state models (i.e., we assume that all the microstates within a macrostate share a common chemical potential in all conditions). To do this, we first define a canonical partition function for each macrostate

$$Z_i = \sum_{\alpha_i} \exp(-E_{\alpha_i}/kT)$$

Using this, we can then calculate effective rate constants for each of the processes discussed above under the assumption that each macrostate has fully thermalised as follows

$$K_{i,X} = \frac{\sum_{\alpha_i} k_{i,X}{}^{(\alpha_i)} \hat{P}_{\alpha_i}}{\sum_{\alpha_i} \hat{P}_{\alpha_i}} = \frac{\sum_{\alpha_i} k_{i,X}{}^{(\alpha_i)} \exp(-(E_{\alpha_i} - \hat{\mu}_i)/kT)}{\sum_{\alpha_i} \exp(-(E_{\alpha_i} - \hat{\mu}_i)/kT)} = \frac{\sum_{\alpha_i} k_{i,X}{}^{(\alpha_i)} \exp(-E_{\alpha_i}/kT)}{Z_i}$$

where $\hat{P}_{\alpha_i}$ is the population of the eigenstate α_i under the assumption of quasi-thermal equilibrium, $\hat{\mu}_i$ is the chemical potential of the ith macrostate under the assumption of quasi-thermal equilibrium and the $k_{i,X}{}^{(\alpha_i)}$ refer to the microscopic rate constants associated with the process X involving macrostate i. Knowing the effective rate constants for all the processes (i.e., $K_{i,j}$, $K_{rec,i}$ and K_{out}), we can then solve the reduced, three-state system, under steady state conditions to find the populations of the three macrostates under the assumption that each macrostate is at quasi-thermal equilibrium. That is, we can solve the set of equations

$$\begin{pmatrix} G \\ 0 \\ 0 \end{pmatrix} = \begin{pmatrix} K_{rec,LE} + K_{LE,CT} + K_{LE,CS} & -K_{CT,LE} & -K_{CS,LE} \\ -K_{LE,CT} & K_{rec,CT} + K_{CT,LE} + K_{CT,CS} & -K_{CS,CT} \\ -K_{LE,CS} & -K_{CT,CS} & K_{CS} + K_{CS,LE} + K_{CS,CT} \end{pmatrix} \begin{pmatrix} \hat{P}_{LE} \\ \hat{P}_{CT} \\ \hat{P}_{CS} \end{pmatrix}$$

The populations found by solving these equations then allow us to calculate the rates at which the various processes happen, since the rate is found by multiplying the rate constant $K_{i,j}$ by the relevant population. Knowing the rates, we can then calculate the net population fluxes between different states and hence quantities such as the CGE and compare these with those which we found when using the grand canonical ensemble.

Results

We use the theory described above to determine which process limits CGE in single component devices at different applied fields and in heterojunction devices across a range of HOMO level offsets. To do this, we calculate the fraction of the total photogenerated flux which is lost to the system via exciton recombination $\left(\frac{R_{rec,LE}}{G}\right)$, CT state recombination $\left(\frac{R_{rec,CT}}{G}\right)$ and charge extraction $\left(\frac{R_{out}}{G}\right)$. The results are shown in **Supplementary Figure 9b-c** and they indicate that exciton recombination outcompetes charge extraction and thus limits CGE at all fields in our single component simulations. In our bilayer simulations, CT recombination becomes a significant loss pathway for HOMO level offsets > 0.30 eV, though we note that the precise value will depend upon the choice of parameters. Thus, our simulations indicate that it is the transition from the LE to the CT state which limits CGE in single component systems and low HOMO level offset heterojunctions, while the transition from CT to CS is limiting heterojunctions with larger HOMO level offsets.

We can relate this result to our experimental data by considering the quantity $1 - \frac{R_{rec,LE}}{G}$ which is equivalent to the flux from the LE to the CT state, $F_{\mathrm{LE,CT}}$ (see **Supplementary Figure 9a**). If we assume that the radiative efficiency of exciton recombination is independent of the applied field/HOMO level offset, this quantity is equivalent to our experimental measurement of charge generation efficiency from the field dependent PL quenching. In the single component case (**Supplementary Figure 9b**), we see that the charge extraction and $F_{\mathrm{LE,CT}}$ have the same dependence on the applied field, in agreement with our measurements (**Figure 2**, main text, and **Supplementary Figures 19-21**). This indicates that recombination losses via the CT are negligible. In the heterojunction case (**Supplementary Figure 9c**), $\frac{R_{rec,CT}}{G}$ becomes significant at large offsets and thus the charge extraction flux, which is equivalent to $F_{CT,CS}$, falls below $F_{\mathrm{LE,CT}}$ since now recombination via the CT is a significant loss pathway.

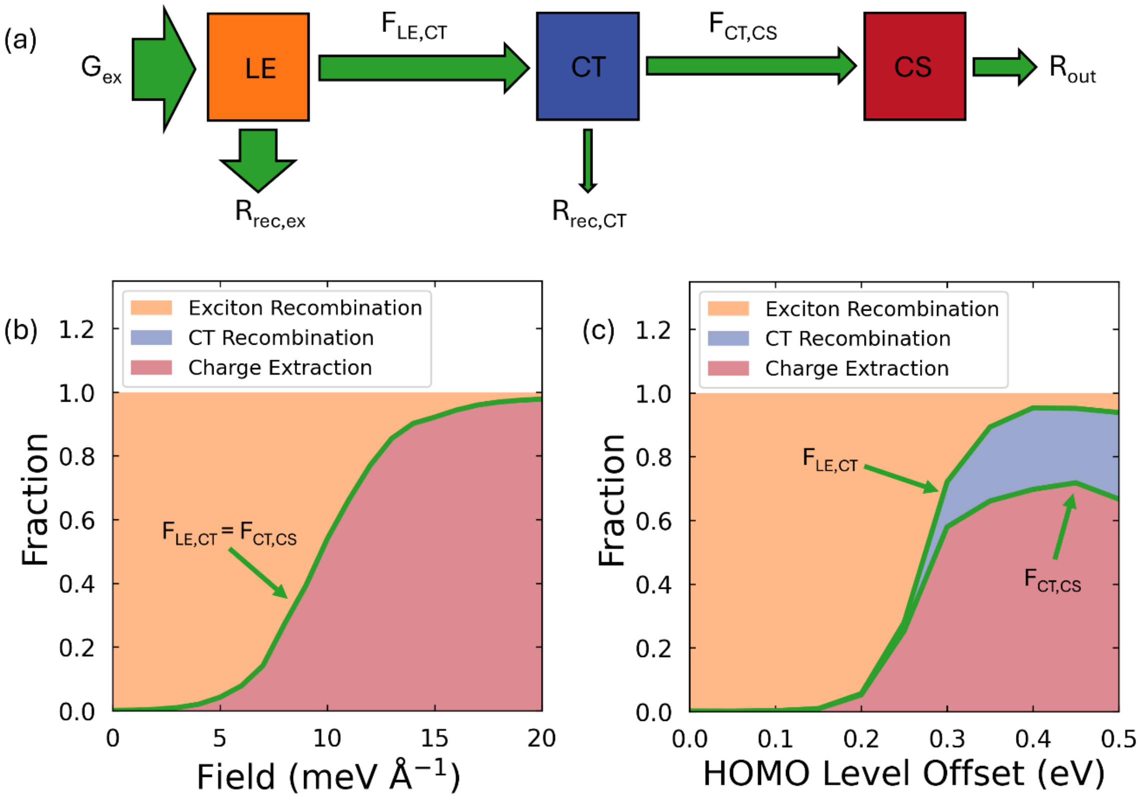

Supplementary Figure 9. (a) A flow chart illustrating the ways in which the generation flux, G_{ex}, can be transferred between local exciton (LE), charge transfer (CT) and charge separated (CS) macrostates and lost from the the system via recombination of either the LE or CT state ($R_{rec,LE}$ and $R_{rec,CT}$, respectively), or via extraction from the CS state (R_{out}). $F_{LE,CT}$ and $F_{CT,CS}$ indicate the net flux from the LE to the CT and the CT to the CS state, respectively. (b-c) The fraction of the total generation flux which is lost to the system via LE recombination, CT state recombination and charge extraction for (b) a single component simulation as a function of the applied field and (c) a bilayer simulation as a function of the HOMO level offset. In both (b) and (c), we have indicated the net fluxes $F_{LE,CT}$ and $F_{CT,CS}$ in green.

Next, we contrast the results obtained from the full model to those we would obtain if we assumed quasi-thermal equilibrium within each macrostate, as is typically done within three-state models (i.e., we assume that the states within each macrostate thermalise before they are either extracted, recombine or undergo population transfer to another macrostate). The CGE as a function of applied field and HOMO level offset are shown for both these scenarios in **Supplementary Figure 10a** and **Supplementary Figure 10b**, respectively. We see that the assumption of quasi-thermal equilibrium overestimates the CGE at low fields and at small HOMO level offsets. This overestimation of the CGE

arises as the assumption of thermal equilibrium within each macrostate leads to an increase in the population of the lowest energy states. These states have the largest energy barrier to 'backward' processes (i.e., CT to LE or CS to CT) and thus the assumption of thermal equilibrium significantly reduces the overall rate of these unwanted processes. The non-thermal populations of the macrostates in our simulation arise from the way we explicitly account for the rates between all states, without assuming that states within a macrostate are more strongly connected to each other than to states in other macrostates. In fact, if we increase the connectivity between states by increasing the electronic coupling, we see that the agreement between results obtained using the canonical and grand canonical ensembles improves significantly (dashed lines in **Supplementary Figure 10**).

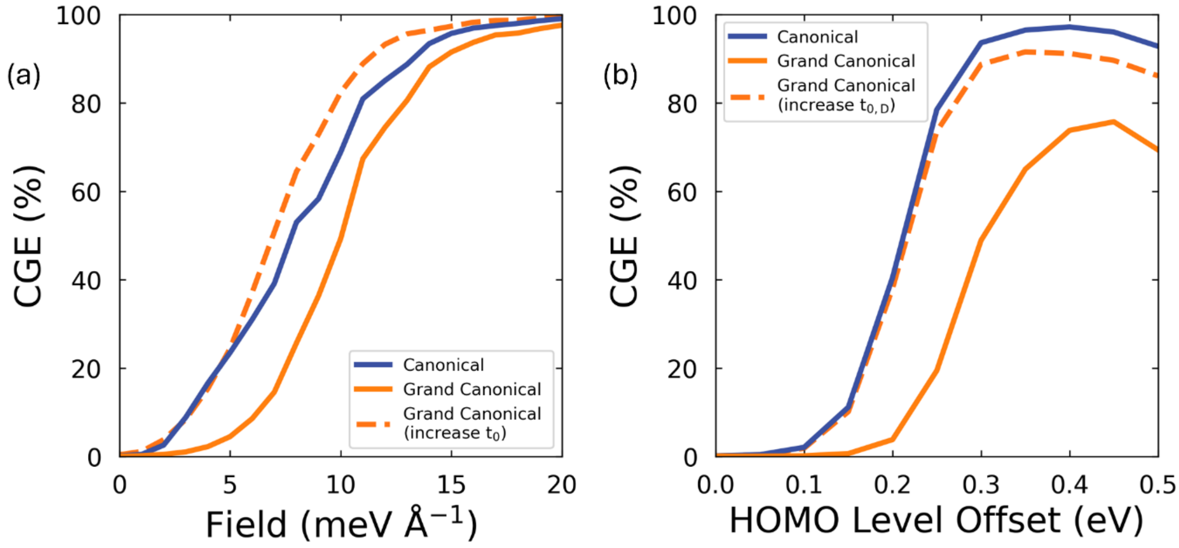

Supplementary Figure 10. The difference between the simulated CGE for the cases where quasi thermal equilibrium is assumed (canonical ensemble) and the more general case, treated in our model, where it is not (grand canonical ensemble) for (a) single component simulations as a function if the applied field and (b) bilayer simulations as a function of the HOMO level offset. In both cases, we show how the agreement between the canonical and grand canonical ensembles can be improved by reducing transport limitations. In (a) we do this by increasing the value of the electronic coupling (t_0) from 3 meV to 5 meV and, in (b), we do this by increasing the electronic coupling in the donor ($t_{0,D}$) from 0.03 meV to 3 meV, while leaving the electronic coupling in the acceptor unchanged.

4 Supplementary Note Four: Results of Model with No Delocalisation

To use the model to investigate the case without significant delocalisation of the excited states, we assumed that coupling between sites could be treated perturbatively. This has two consequences:

1. The basis states discussed in Section 1.1 become the eigenstates of the system as the Hamiltonian is diagonal in the site basis if the electronic/excitonic couplings are treated perturbatively.
2. The rate of population transfer between the states can be calculated using Marcus-Levich-Jortner theory (see **Section 1.3.3**). However, we note that the generalised version of the theory is unnecessary here as there is no delocalisation i.e., every excited state has an IPR of 1. For exciton-to-exciton state transfer, we use a total reorganisation energy of twice the exciton reorganisation energy while, for CT-to-CT and exciton-to-CT transitions, we use a total reorganisation energy of twice the polaron reorganisation energy.

Aside from these changes, the other aspects of the model (e.g., generation, extraction etc.) remain as described in **Sections 1.1-1.4**, meaning that the CGE is calculated by solving Pauli's master equation to obtain the populations of the different excited states under steady state conditions (see **Section 1.4**).

Our results are shown for the single component case in **Supplementary Figure 11** and for the bilayer case in **Supplementary Figure 12**. We show the results calculated with delocalisation ('Redfield') adjacent to those calculated without delocalisation ('MLJ') such that the two situations can be compared more easily. We observe the same trends in CGE as we vary the model's parameters with or without the inclusion of delocalisation. However, the presence of delocalisation facilitates charge generation at lower fields/HOMO level offsets.

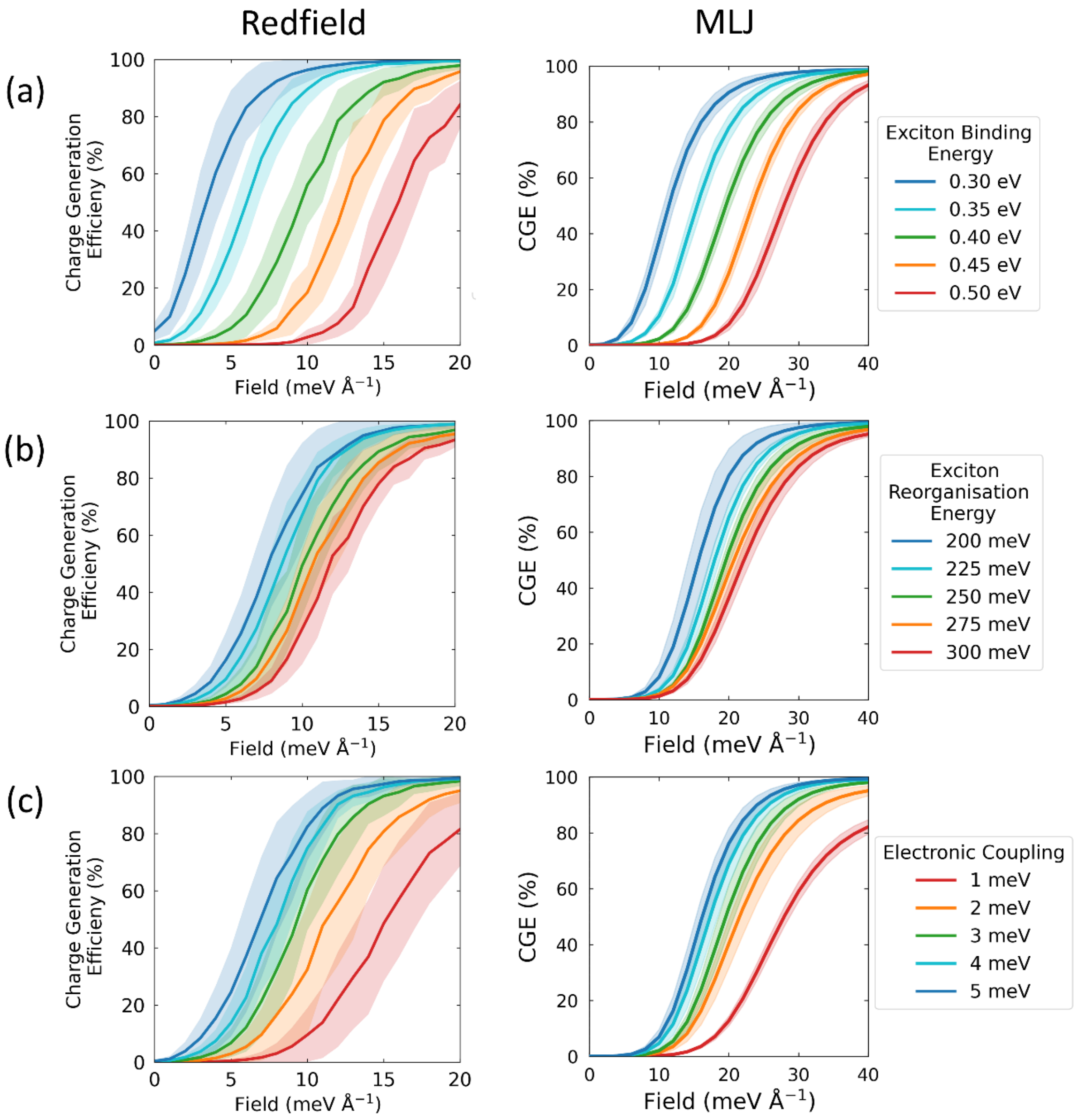

Supplementary Figure 11. Simulated charge generation efficiency versus applied field for the cases with (left-hand column, 'Redfield') and without (right-hand column, 'MLJ) delocalisation. We show results obtained by varying the input model parameters: (a) exciton binding energy, (b) exciton reorganisation energy and (c) electronic coupling. The shaded intervals indicate the standard deviation from 20 simulations with different realisations of the static disorder and the solid lines indicate the mean value.

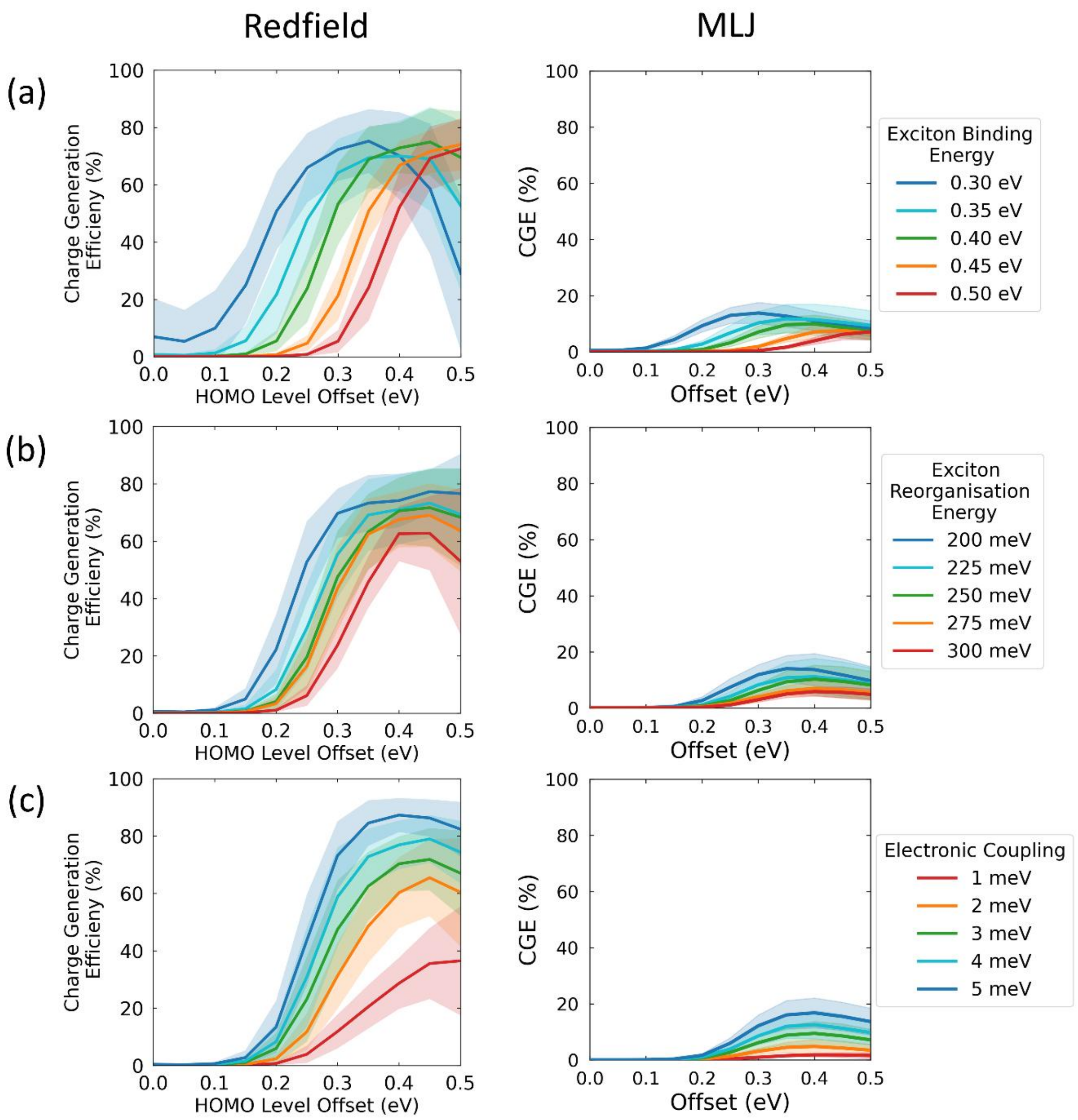

Supplementary Figure 12. Simulated charge generation efficiency versus HOMO level offset for the cases with (left-hand column, 'Redfield') and without (right-hand column, 'MLJ') delocalisation. We show results obtained by varying the input model parameters: (a) exciton binding energy, (b) exciton reorganisation energy and (c) electronic coupling. The shaded intervals indicate the standard deviation from 20 simulations with different realisations of the static disorder and the solid lines indicate the mean value.

5 Supplementary Figures

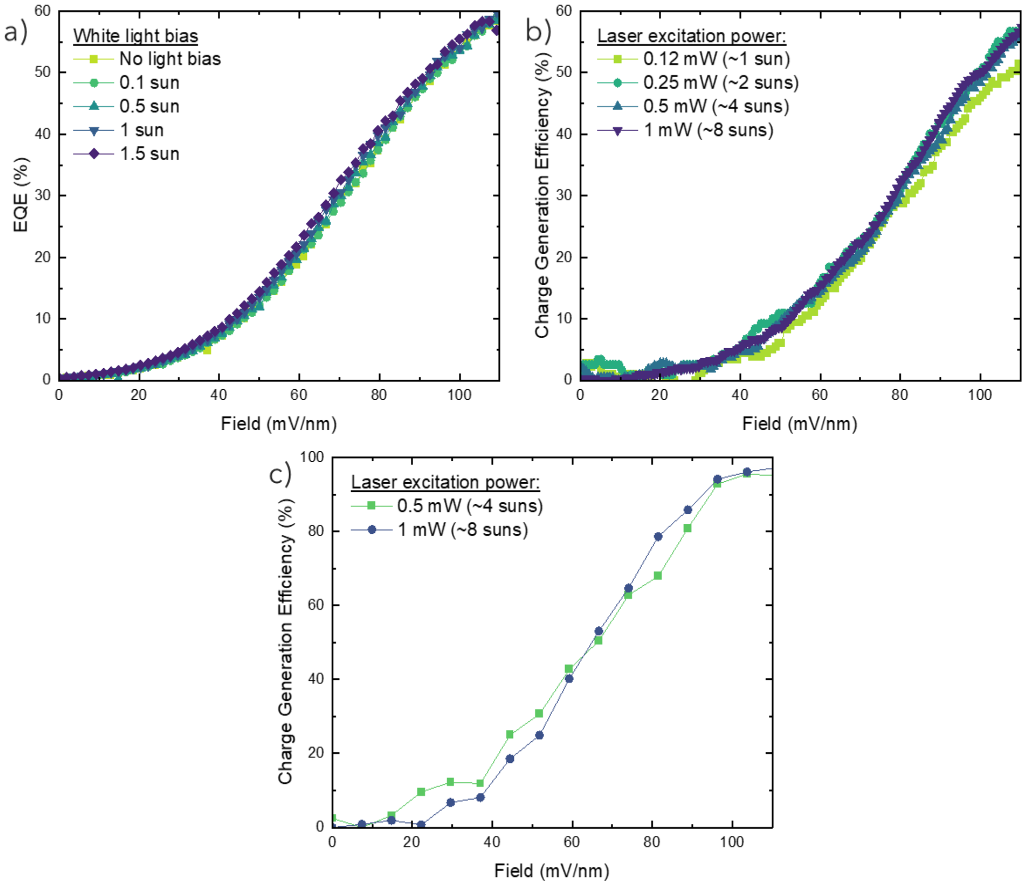

Supplementary Figure 13. (a) Background light intensity dependence of EQE as a function of an applied reverse field for a single component Y5 device. (b,c) Excitation intensity dependence of charge generation efficiency inferred from (b) steady-state photoluminescence and (c) time-resolved photoluminescence as a function of an applied reverse field for the same single component Y5 device. In (b,c), the sample was excited at 485 nm. The lack of background or excitation intensity dependence in these measurements indicates that bimolecular recombination pathways are not significant at the charge carrier densities probed in these experiments.

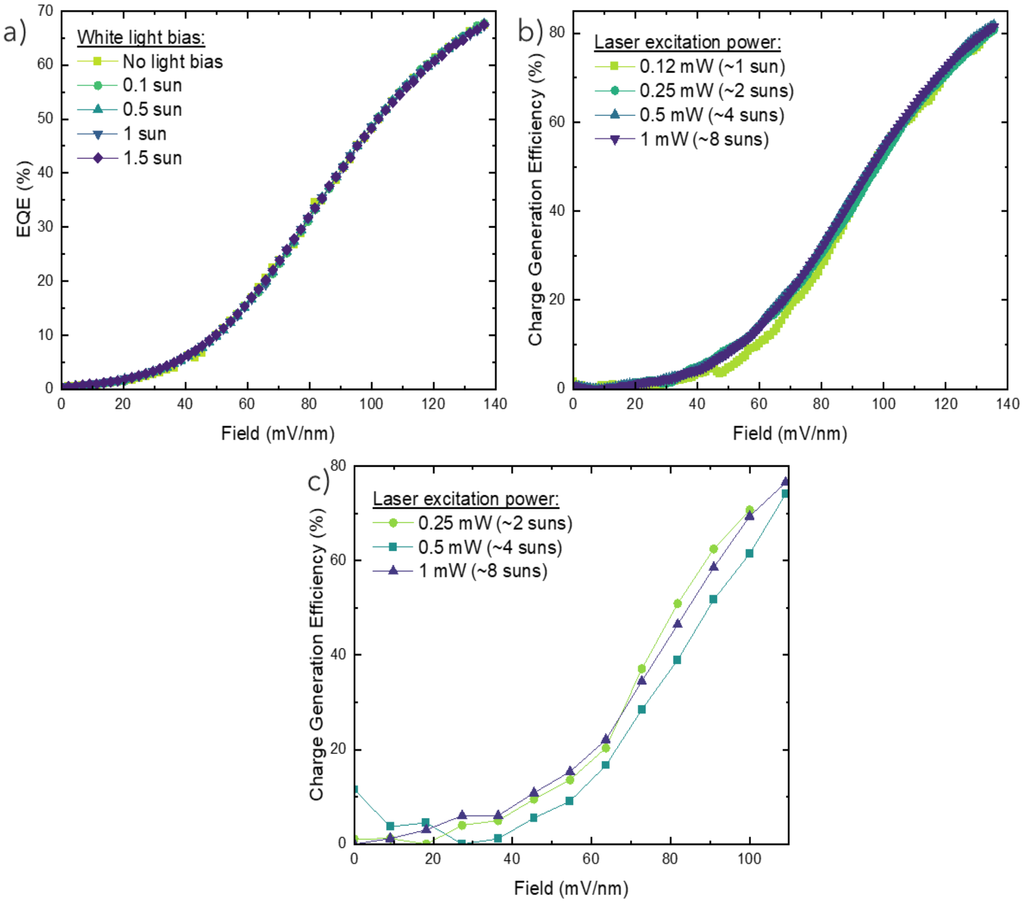

Supplementary Figure 14. (a) Background light intensity dependence of EQE as a function of an applied reverse field for a single component ITIC device. (b,c) Excitation intensity dependence of charge generation efficiency inferred from (b) steady-state photoluminescence and (c) time-resolved photoluminescence as a function of an applied reverse field for a similar single component ITIC device. In (b,c), the sample was excited at 485 nm. The lack of background or excitation intensity dependence in these measurements indicates that bimolecular recombination pathways are not significant at the charge carrier densities probed in these experiments.

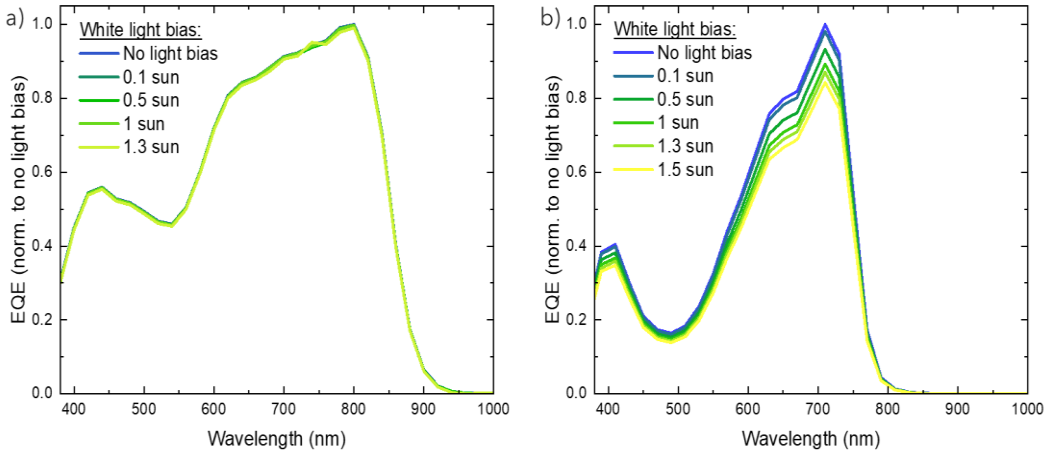

Supplementary Figure 15. Background light intensity dependent EQE spectra for a planar heterojunction device with the structure ITO/CuSCN/Br-2PACz/Acceptor/BCP/Ag where the acceptor is (a) Y5 and (b) ITIC. For the Y5 device, the lack of light intensity dependence indicates that bimolecular recombination does limit the current extracted at short circuit. In the case of the ITIC device, the EQE has a moderate dependence on the background light intensity which suggests that nongeminate recombination losses may be a factor limiting charge generation at short circuit. However, as the light intensity dependence is weak, this is unlikely to be the main cause of the lower EQEs obtained by the IT-family bilayer devices.

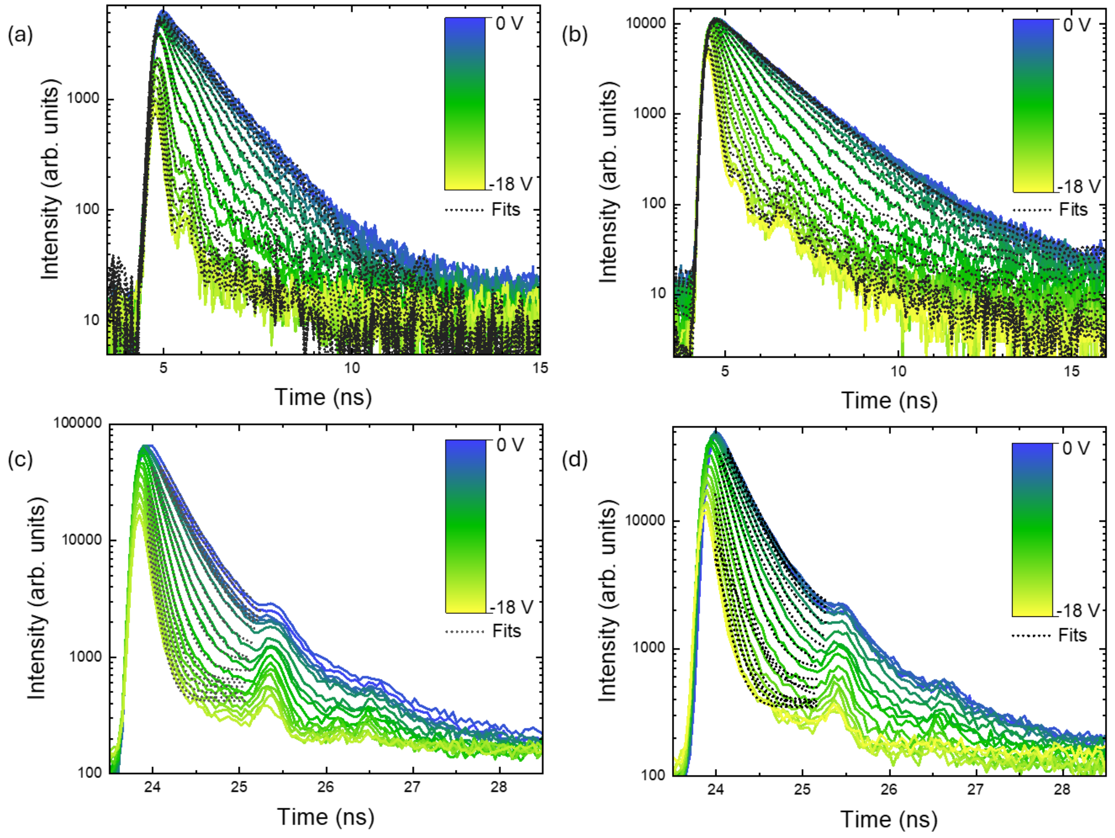

Supplementary Figure 16. Collected tr-PL transients for single component device*s* with (a) Y6, (b) Y5, (c) ITIC or (d) IT4F as the active layer, plotted with corresponding fits. The respective probed wavelengths are (a) 950 nm, (b) 955 nm, (c) 785 nm and (d) 815 nm. In general, Y5 and Y6 (panels (a) and (b)) have longer lifetimes compared to ITIC and IT4F (panels (c) and (d)), the latter having lifetimes close to the instrument response function (IRF) of our setup. Thus, fitting the ITIC and IT4F transients with IRF convoluted with a decaying exponential was challenging. Instead, these data were cut and fitted by a single decaying exponential within the region where this approximation was valid. Further, the data features the common artefact corresponding to re-excitation of the sample due to reflections in the detection system (around 1.4 and 2.5 ns after peak).

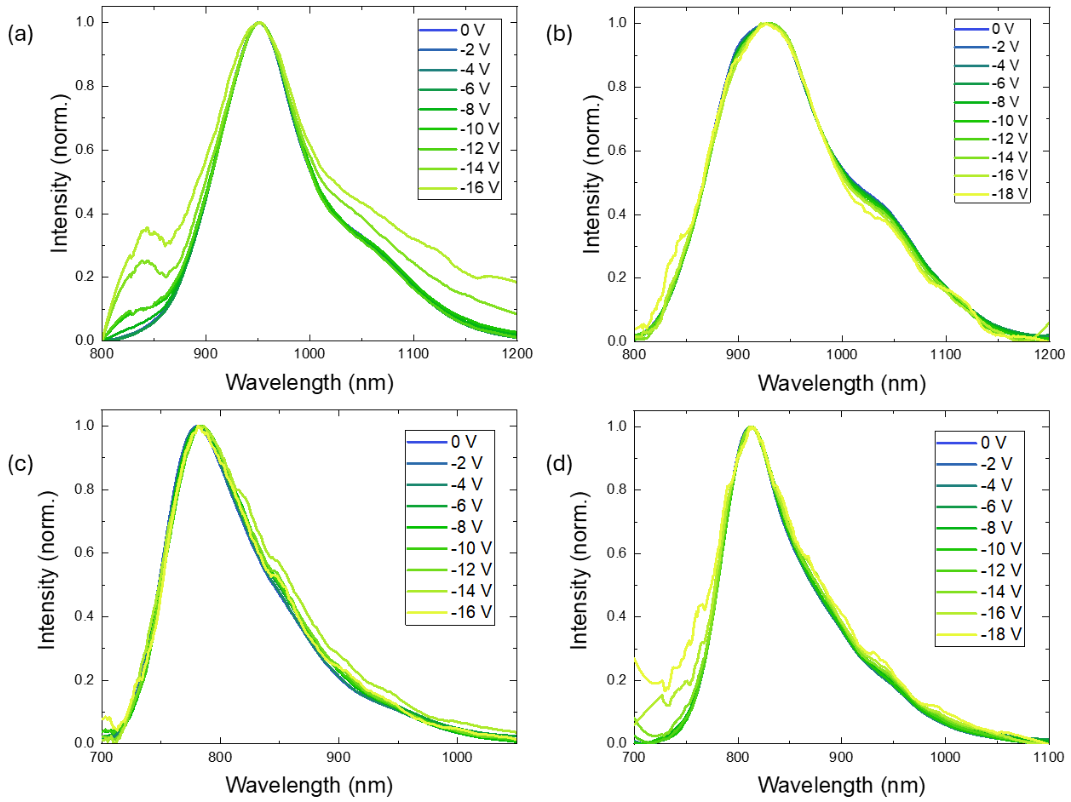

Supplementary Figure 17. Corrected and normalised PL spectra at different applied biases of single component device with (a) Y6, (b) Y5, (c) ITIC or (d) IT4F as the active layer. Within the signal to noise of the measurement, the PL spectra of each material retain the shape across the entire range of applied biases. This signifies that the relative emission from the states being probed by this measurement are independent of the applied field. As we would expect the largest contribution to the PL at zero field to come from states with excitonic character, this observation suggests that the emission from the exciton dominates throughout the measurement and that there is a negligible contribution from emissive CT states. We note that, in the case of Y6, the lowest excited states may have some CT as well as Frenkel character, due to the strong electronic coupling in this material [20]. These hybrid states still dominate the emission, and, in our analysis of Y-family PL data, we treat them as the emissive states.

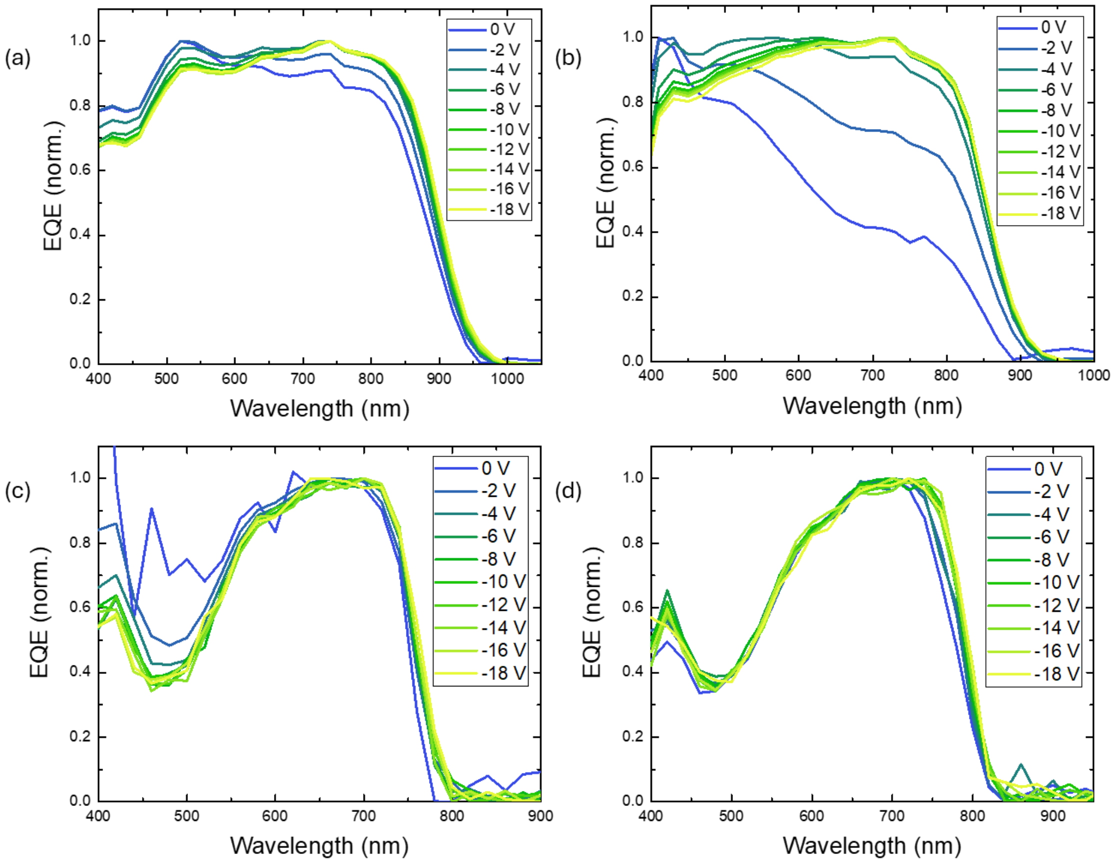

Supplementary Figure 18. Normalised EQE spectra at different applied biases of single component device with (a) Y6, (b) Y5, (c) ITIC or (d) IT4F as the active layer. Within the signal to noise of the measurement, the EQE spectra of ITIC and IT4F retain the shape across the range of applied biases. This signifies that the relative occupations of the states being probed by this measurement are independent of the applied field. For Y5 and Y6, we see that higher energy photons contribute more to the photocurrent to at applied biases < -6 V. This indicates that the charge generation efficiency has some dependence on the exciton energy. For applied biases more negative than -6 V, the EQE spectra maintain their shape.

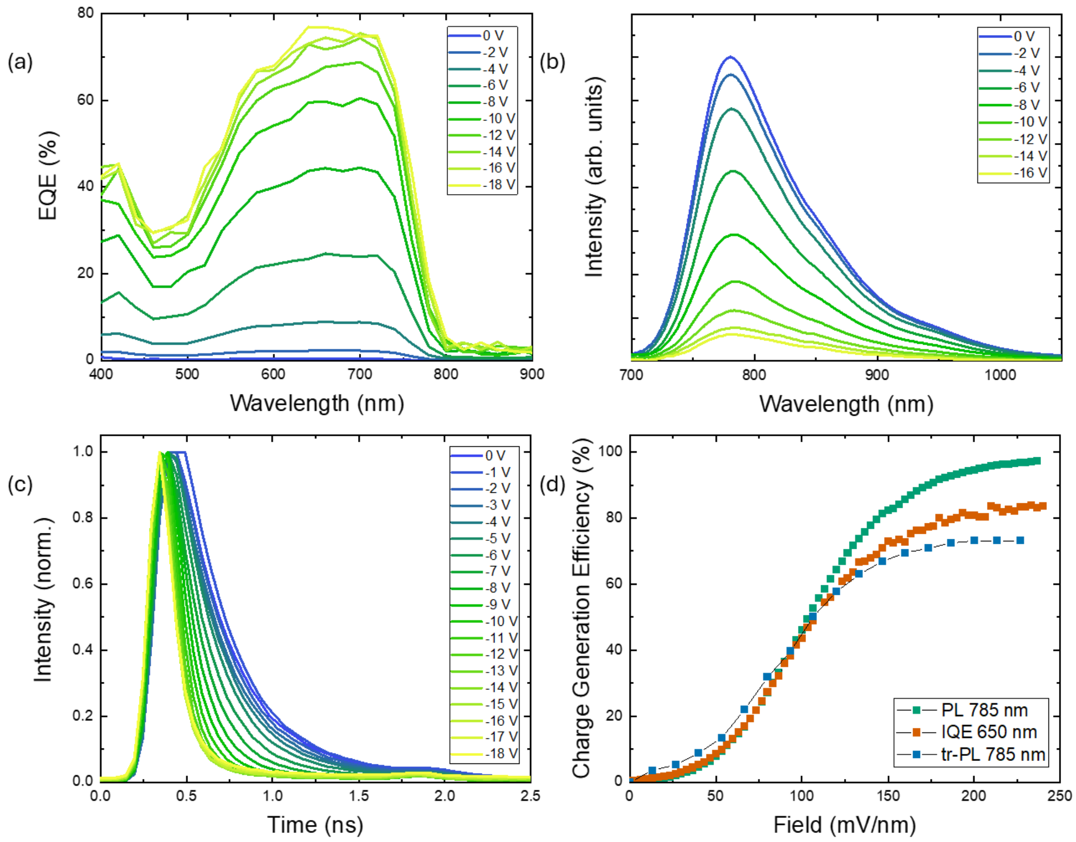

Supplementary Figure 19. Bias dependent (a) EQE, (b) PL and (c) tr-PL (at 785 nm) for a single-component ITIC device. (d) Comparison of the field dependent IQE to the charge generation efficiency estimated from PL and tr-PL, at respective maximum wavelength.

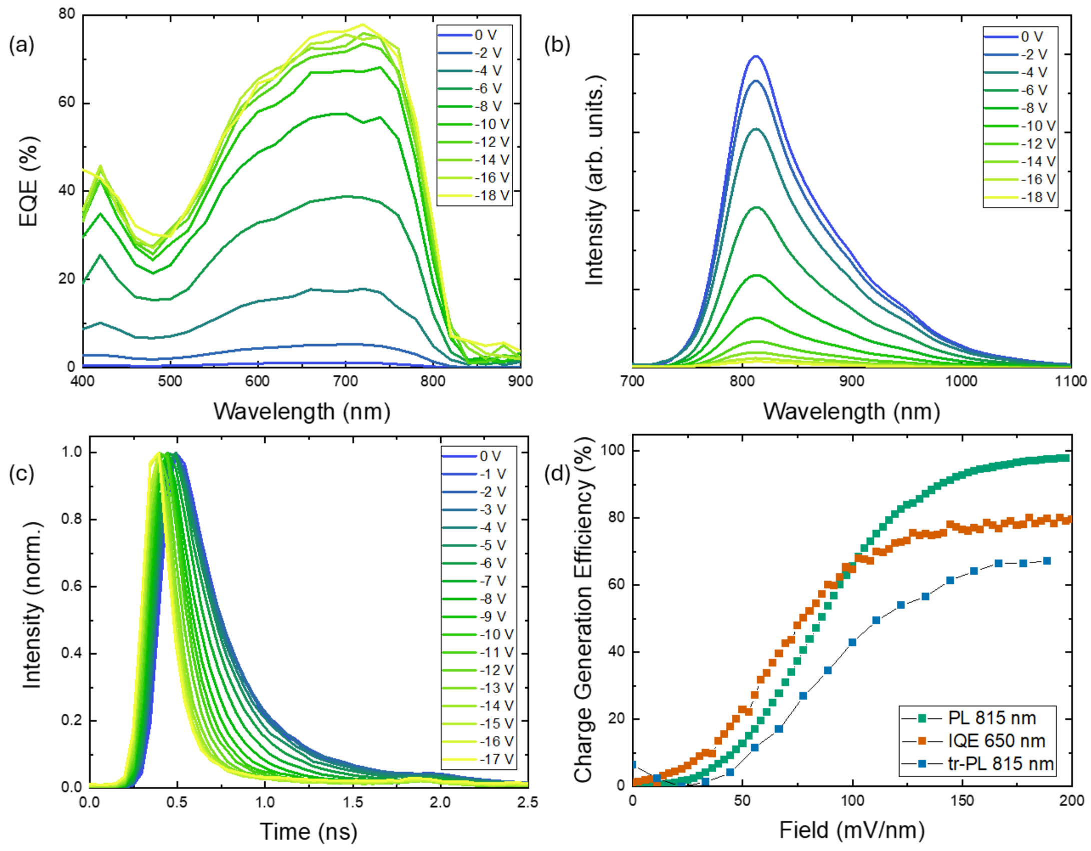

Supplementary Figure 20. Bias dependent (a) EQE, (b) PL and (c) tr-PL (at 815 nm) for a single-component IT4F device. (d) Comparison of the field dependent IQE to the charge generation efficiency estimated from PL and tr-PL, at respective maximum wavelength.

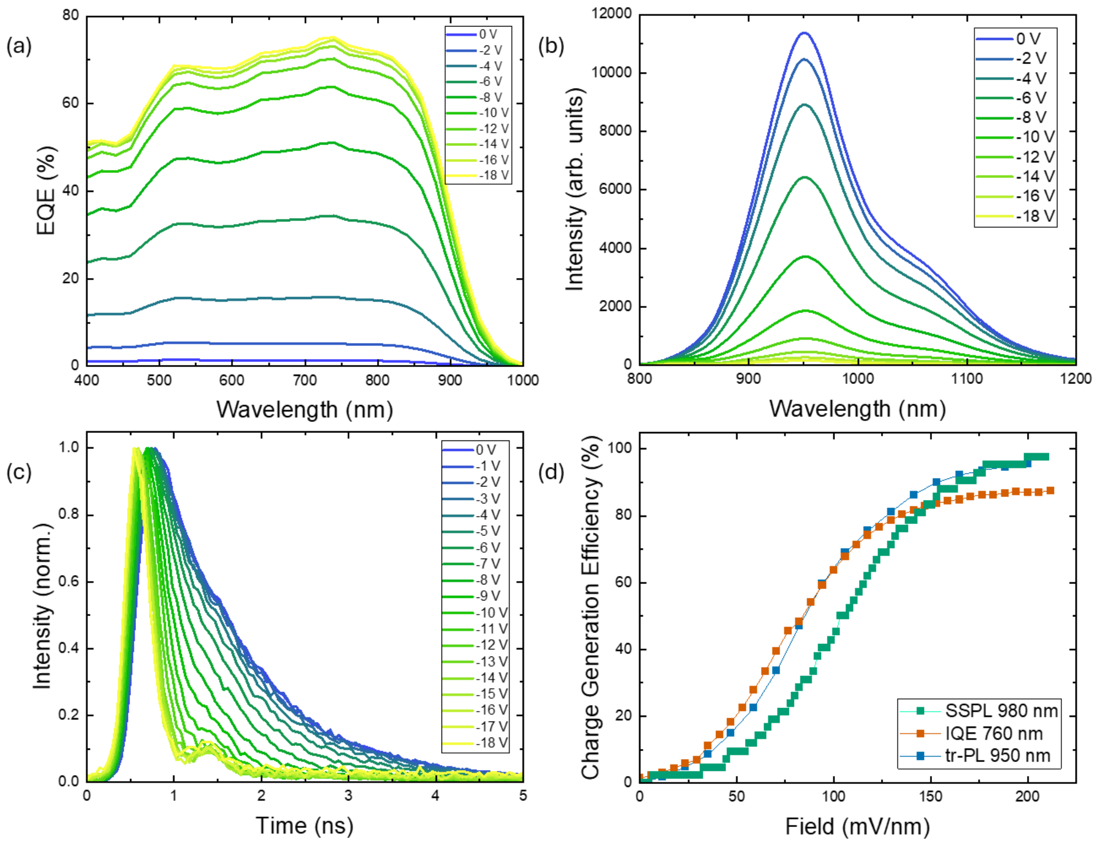

Supplementary Figure 21. Bias dependent (a) EQE, (b) PL and (c) tr-PL (at 960 nm) for a single-component Y6 device. (d) Comparison of the field dependent IQE to the charge generation efficiency estimated from PL and tr-PL, at respective maximum wavelength.

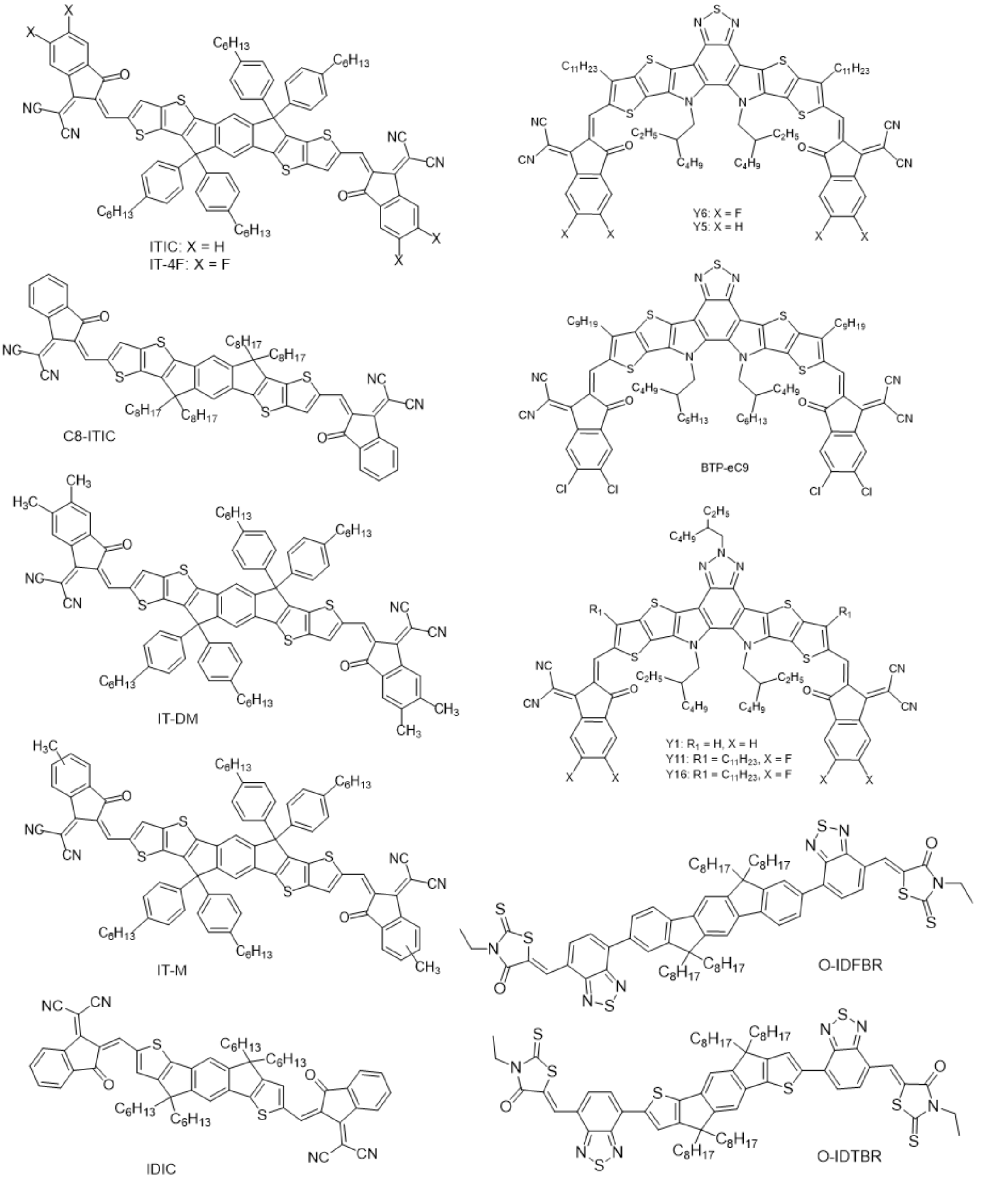

Supplementary Figure 22. Non-fullerene acceptors investigated in this study. Full names are provided in **Supplementary Table 6**.

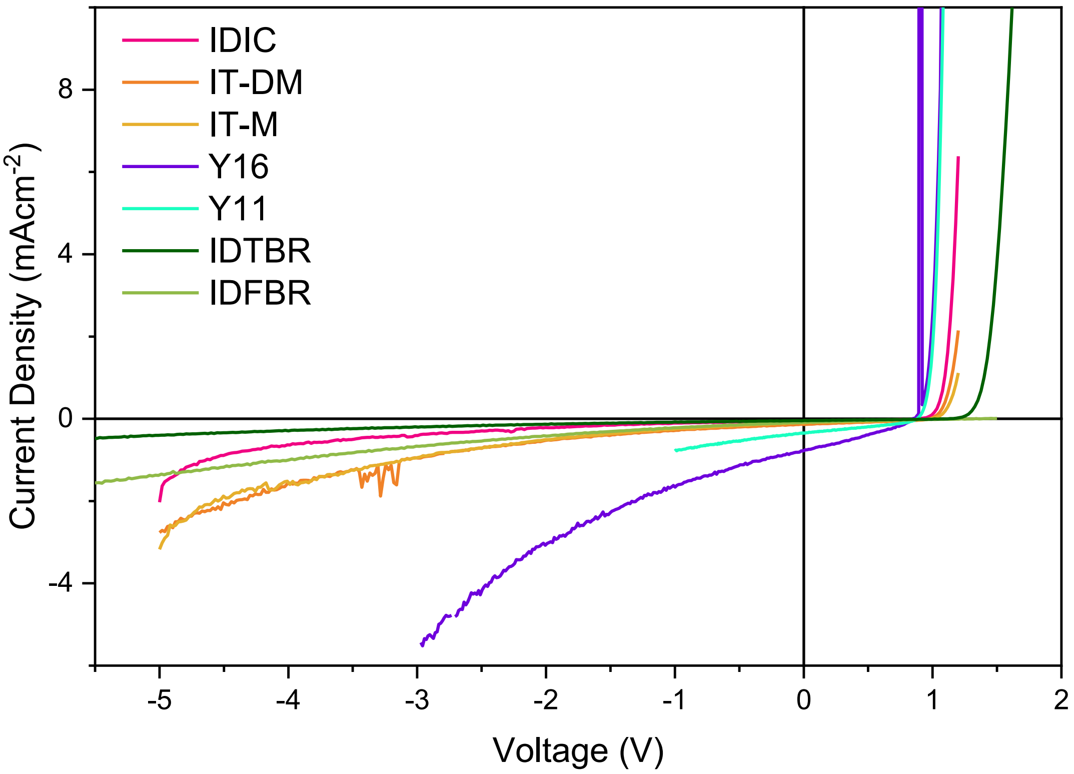

Supplementary Figure 23. Current density-voltage current curve under AM1.5 of single component ITO/Br-2PACz/NFA/BCP/Ag devices. JV parameters are provided in **Supplementary Table 1**.

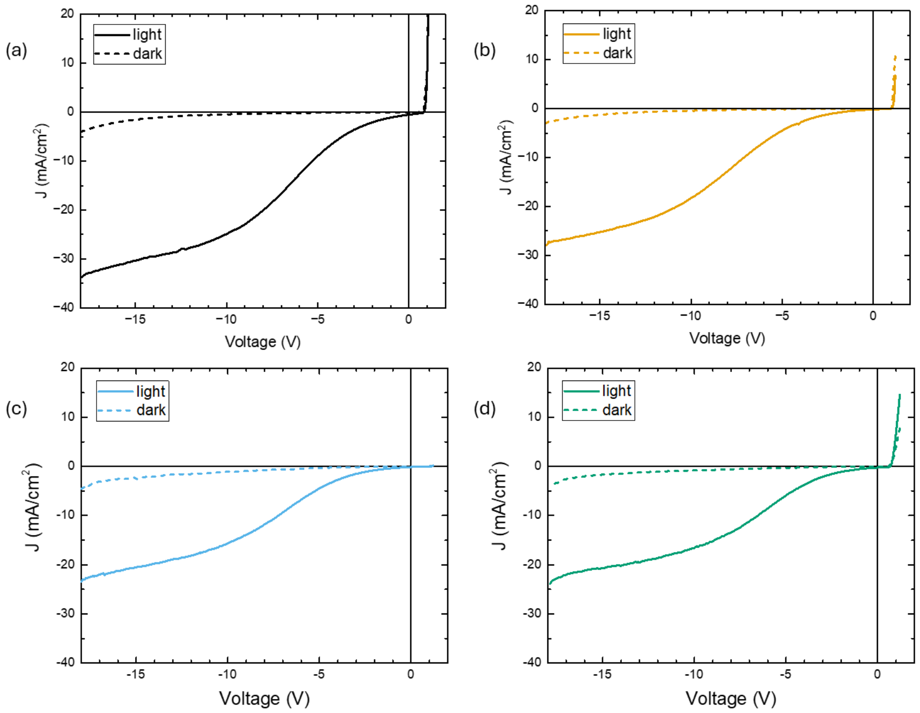

Supplementary Figure 24. Current-voltage current curve in the dark (dashed line) and under AM1.5 illumination (solid) of single component ITO/Br-2PACz/NFA/BCP/Ag devices with (a) Y6, (b) Y5, (c) ITIC and (d) IT4F in the active layer. JV parameters are provided in **Supplementary Table 1**. For these JVs, we have measured over the voltage range 1.2V to -18V to demonstrate that the extracted current at high reverse biases is always at least half an order of magnitude higher under illumination than in the dark. This indicates that the field-dependence of our EQE and PL measurements is due to a field-dependent photocurrent, as opposed to dark carrier injection.

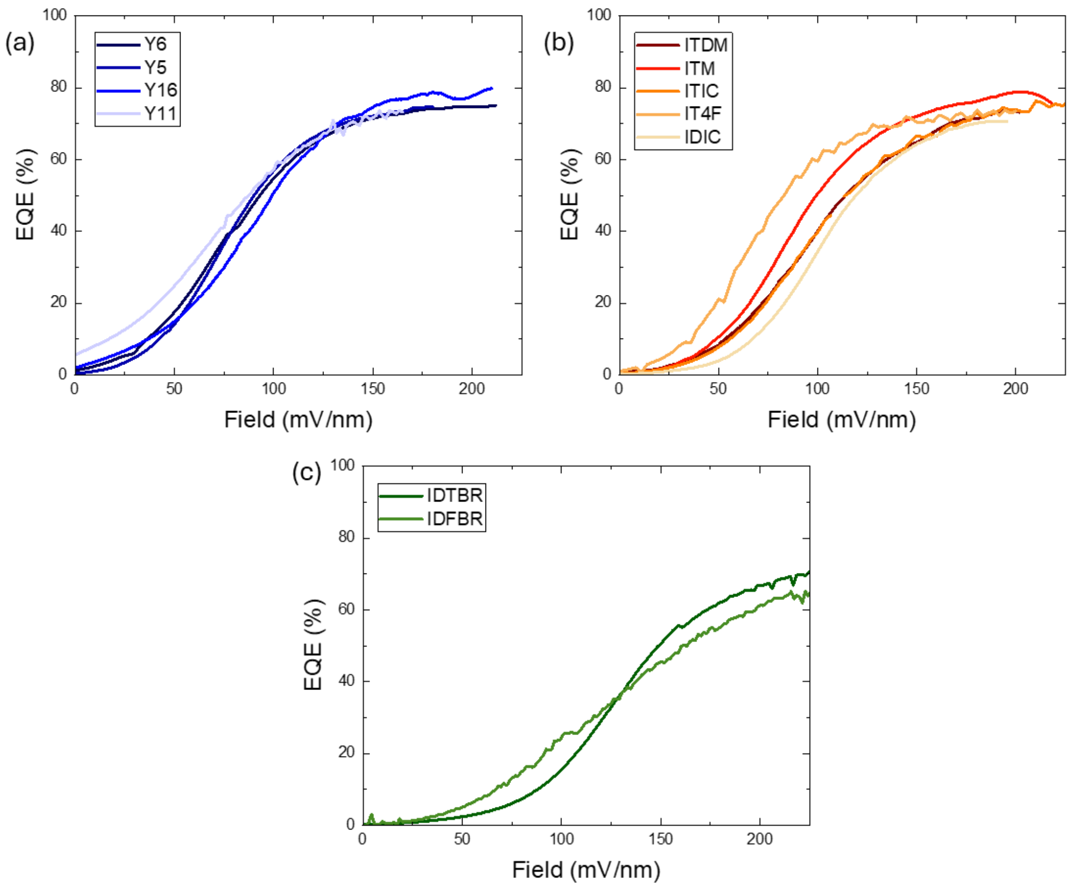

Supplementary Figure 25. EQE data from **Figure 3** in the main text for (a) the Y-Series, (b) the IT-Series and (c) the xBr-Series. EQEs were measured at 760 nm for the Y-Series, 650 nm for the IT-Series, 680 nm for IDTBR and 520 nm for IDFBR .

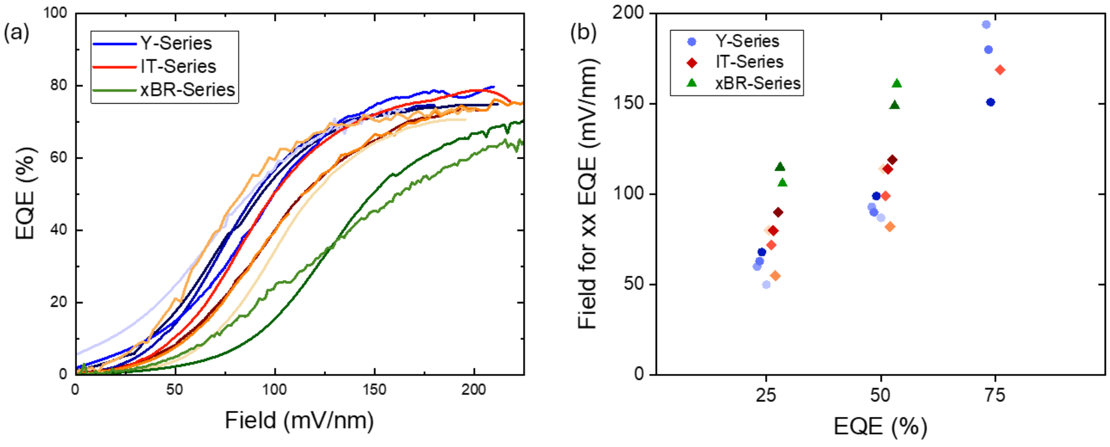

Supplementary Figure 26. (a) EQE data of all single component devices measured in this study and (b) fields required to achieve 25%, 50% and 75% EQE. These data and the materials' refractive indices (**Supplementary Figure 32**) were used to calculate the IQE data shown in **Figure 3** of the main text. EQEs were measured at 760 nm for the Y-Series, 650 nm for the IT-Series, 680 nm for IDTBR and 520 nm for IDFBR.

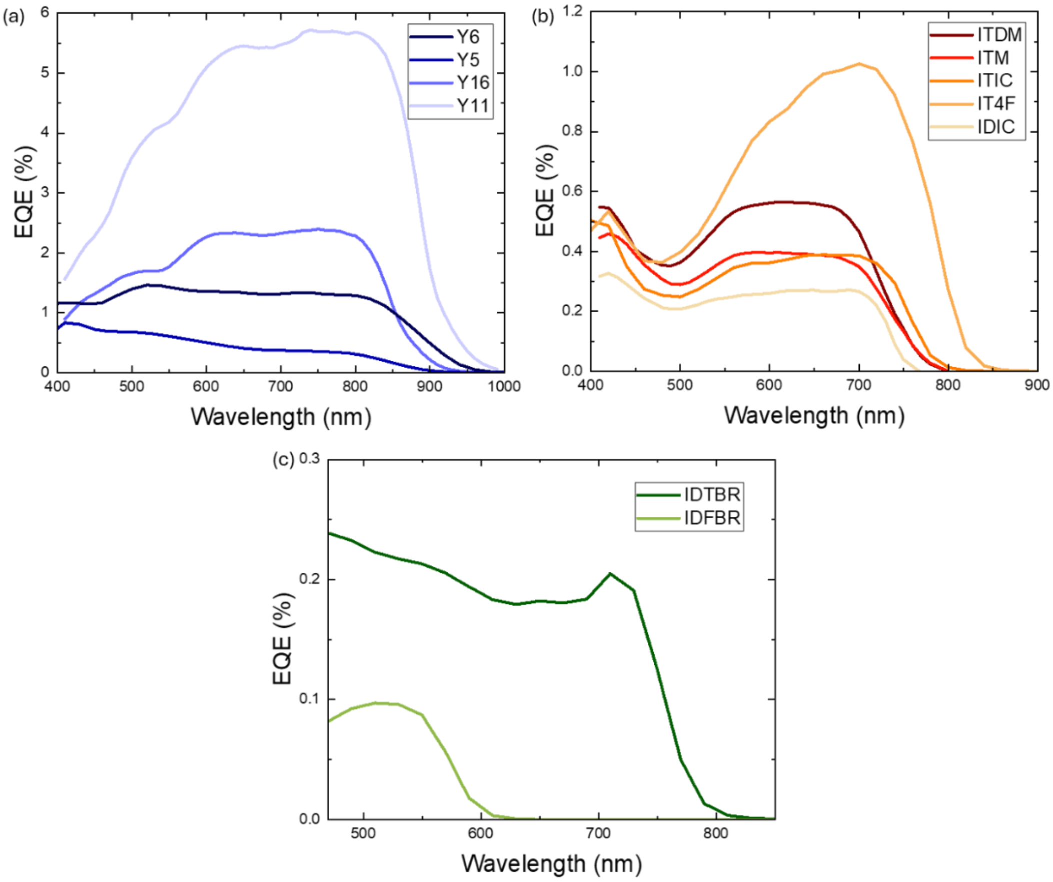

Supplementary Figure 27. EQE spectra of all single component devices (a) the Y-Series, (b) the IT-Series and (c) the xBr-Series, under no applied bias.

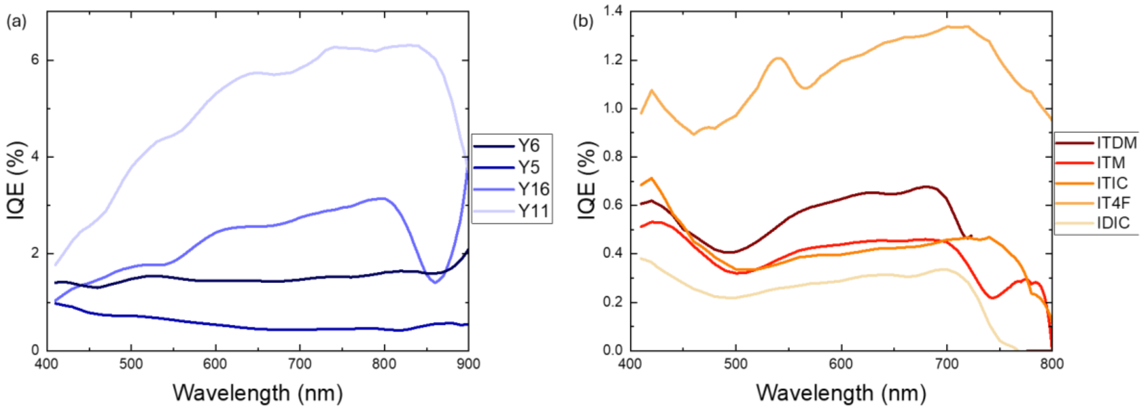

Supplementary Figure 28. IQE spectra of all single component devices under no applied bias, modelled using the measured EQE and refractive indices of each material (see **Supplementary Figure 32**).

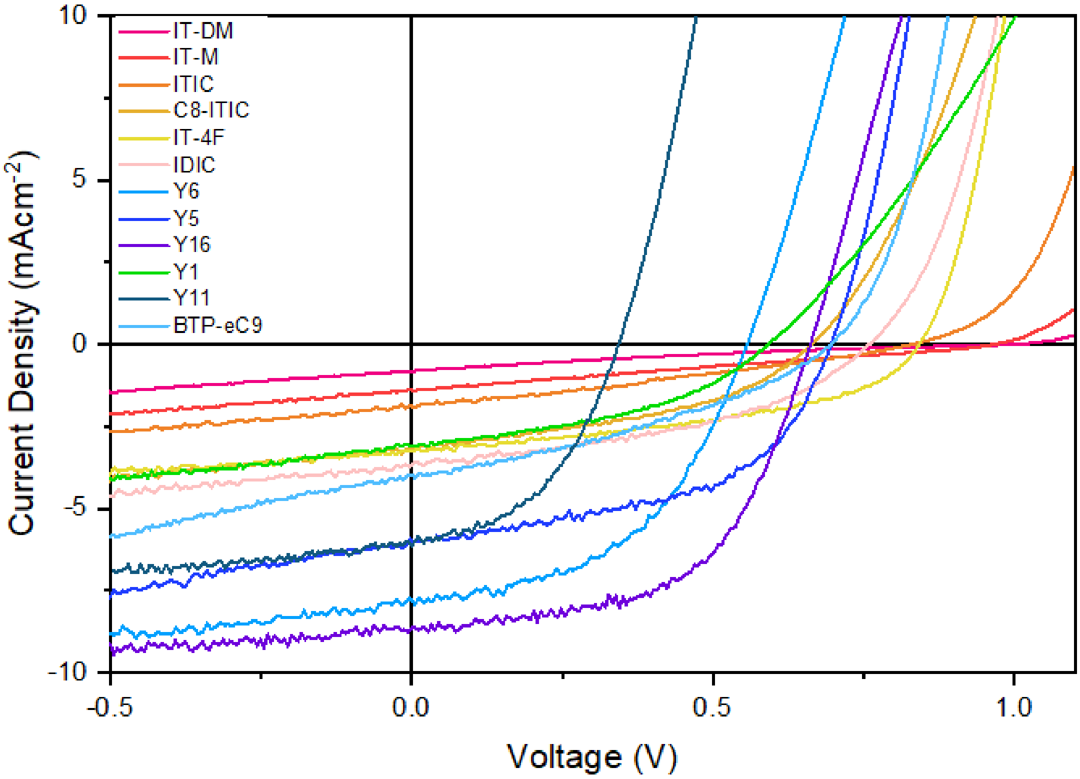

Supplementary Figure 29. Current-voltage characteristics under AM1.5 illumination of ITO/CuSCN/Br-2PACz/NFA/BCP/Ag solar cells with different NFAs.

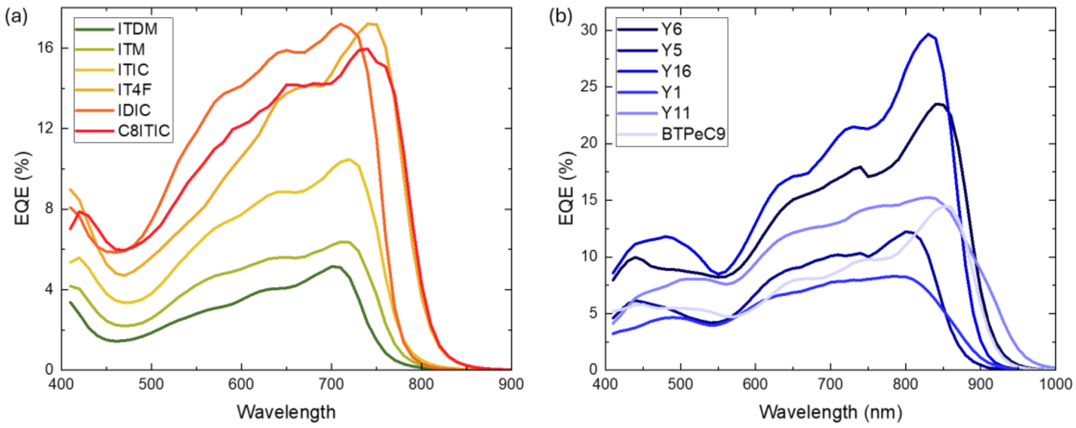

Supplementary Figure 30. Measured EQE of bilayer devices (ITO/CuSCN/Br-2PACz/NFA/BCP/Ag) in (a) IT-Series and (b) Y-Series. These data and the materials' refractive indices (**Supplementary Figure 32**) were used to calculate the IQE data shown in **Supplementary Figure 31**.

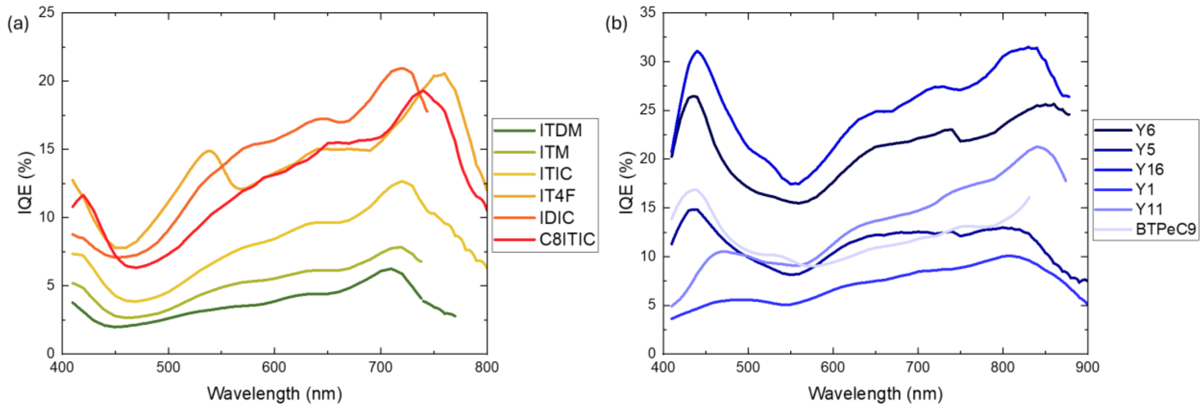

Supplementary Figure 31. Calculated IQE spectra of bilayer devices (ITO/CuSCN/Br-2PACz/NFA/BCP/Ag) in (a) IT-Series and (b) Y-Series.

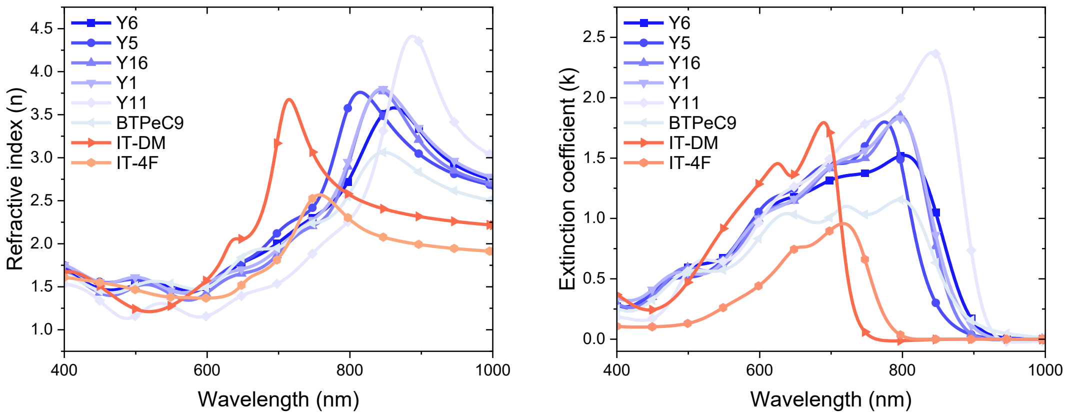

Supplementary Figure 32. Complex refractive index data for the materials studied here. Additional refractive indices were taken from [21], [22] and [23].

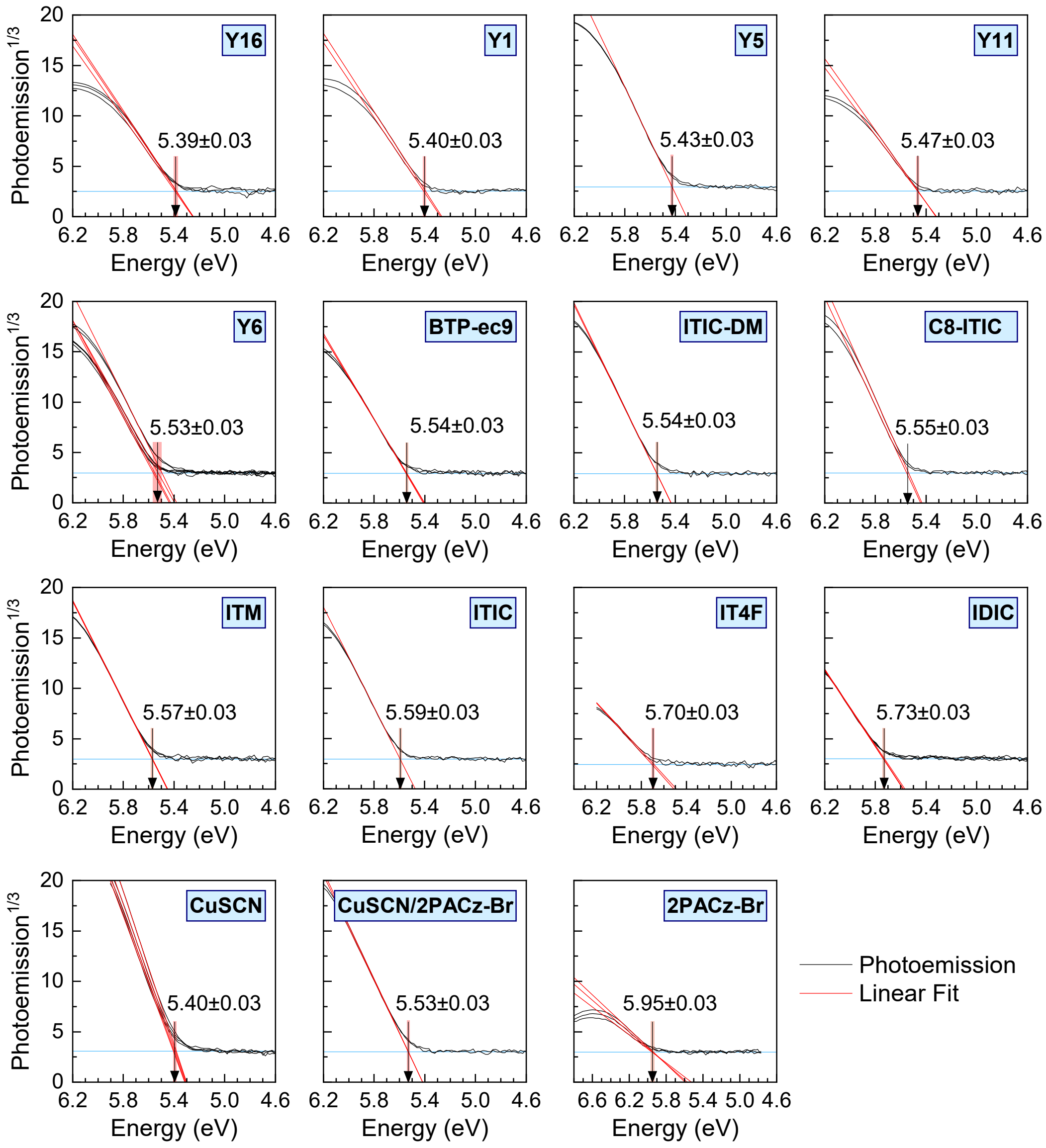

Supplementary Figure 33. Ambient Pressure Photo Emission Spectra measured on KP Technology Ltd's APS02 system. The Ionisation energy is given by the intersection between the background level and the linear fit of the cube root of the emission tail.

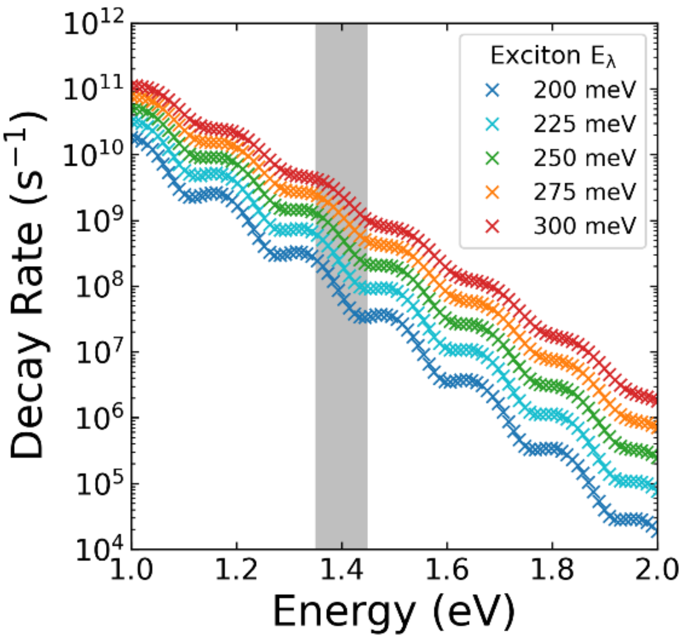

Supplementary Figure 34. Effect of changing the exciton's reorganisation energy on the exciton decay rate as calculated using generalised Marcus-Levich-Jortner. The grey band indicates the energy range in which the singlet states are found. We provide details of how the total reorganisation energy is split between the inner and outer components in **Supplementary Table 5**.

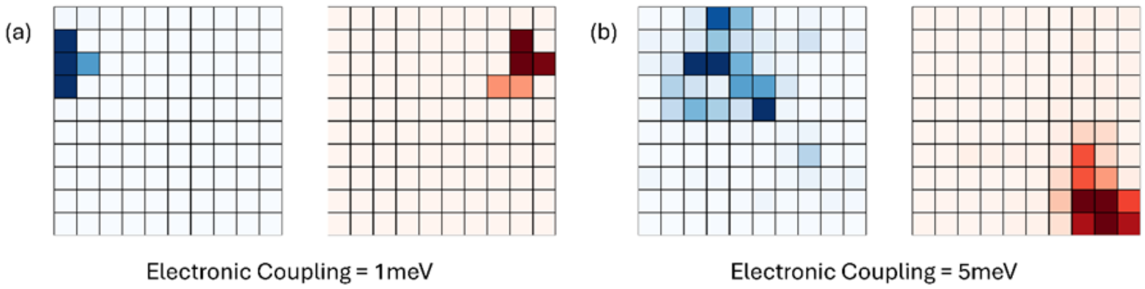

Supplementary Figure 35. Effect of (a) low and (b) high electronic coupling on the spatial distribution of the states across the lattice in single component simulations. The electron (blue) and hole (red) distributions have been shown on different lattices for clarity.

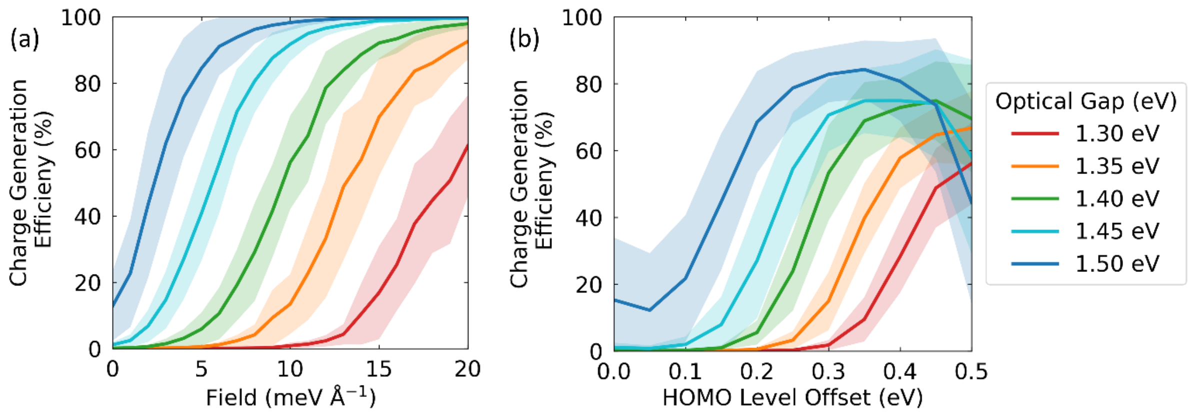

Supplementary Figure 36. Effect of the optical gap on charge generation efficiency (CGE) in (a) single component and (b) bilayer simulations. The optical gap was varied by changing the value of K_0, while J_0 and the acceptor's electronic bandgap were held constant. Thus, a larger optical gap also corresponds to a smaller exciton binding energy, which is why the CGE curves shift to higher fields/offsets as the optical gap decreases. Additionally, simulations with a larger optical gap also benefit from a longer exciton lifetime via the energy gap law (**Supplementary Figure 34**). The shaded intervals indicate the standard deviation from 20 simulations with different realisations of the static disorder and the solid lines indicate the mean value.

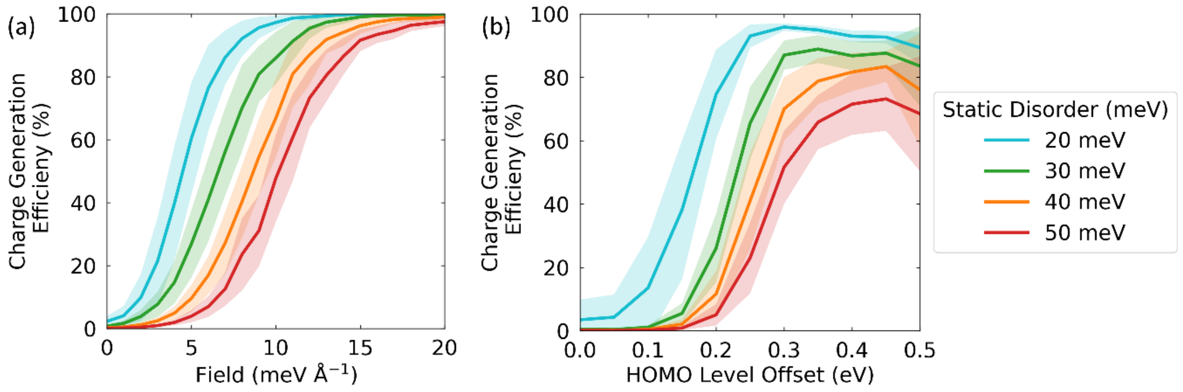

Supplementary Figure 37. Effect of the excitonic coupling on charge generation efficiency (CGE) in (a) single component and (b) bilayer simulations. A larger excitonic coupling gives a small benefit to CGE in single component simulations as the delocalisation of the exciton reduces its decay rate (see **Section 1.3.3**). In the bilayer simulations, the effect is more pronounced which may suggest that the CGE in our simulations is limited by exciton diffusion to the donor:acceptor interface. The shaded intervals indicate the standard deviation from 20 simulations with different realisations of the static disorder and the solid lines indicate the mean value.

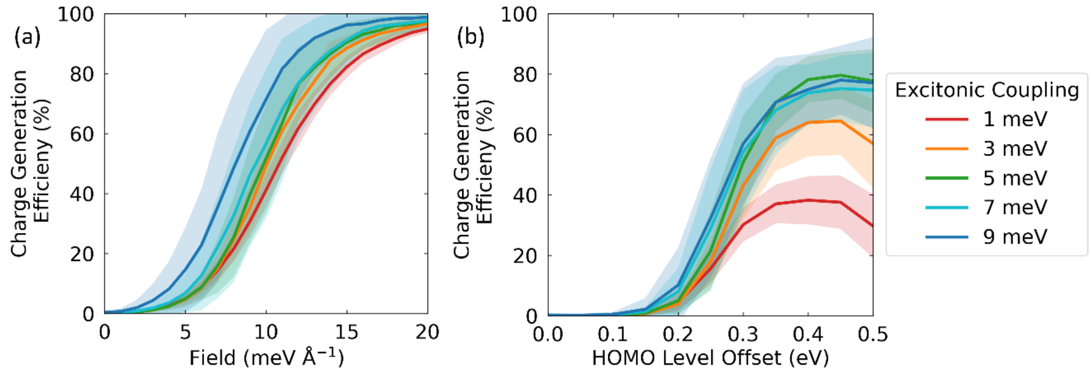

Supplementary Figure 38. Effect of the static (Gaussian) disorder on charge generation efficiency (CGE) in (a) single component and (b) bilayer simulations. A lower static disorder improves CGE as this facilitates delocalisation thereby increasing the rates of population transfer and decreasing the rate at which excited states decay to the ground. The shaded intervals indicate the standard deviation from 20 simulations with different realisations of the static disorder and the solid lines indicate the mean value.

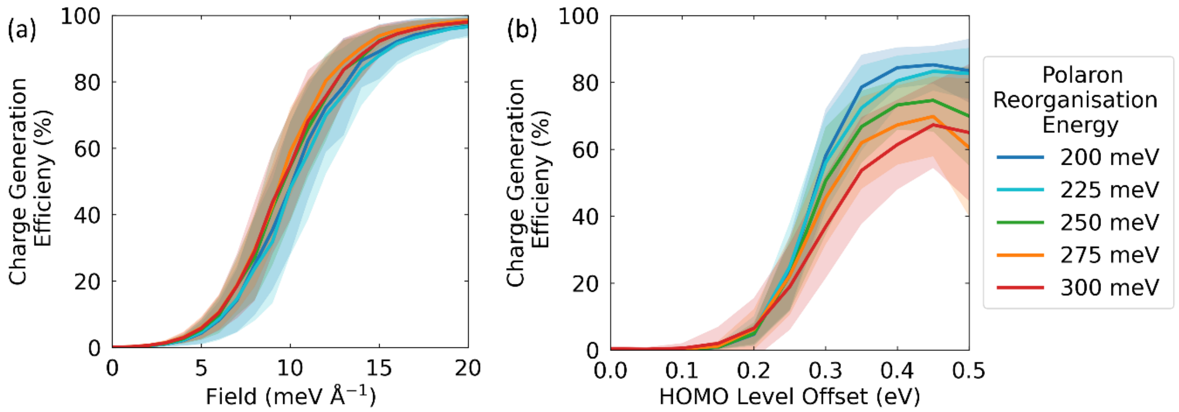

Supplementary Figure 39. Effect of the total reorganisation energy of the polaron on charge generation efficiency (CGE) in (a) single component and (b) bilayer simulations. In the single component system, changing this parameter does not affect the CGE as the CGE is limited by the rate of exciton splitting, rather than subsequent charge transfer state separation (see main text). In the bilayer case, the yield increases at smaller values of the polaron reorganisation energy due to the consequent increase in the CT state lifetime (see **Section 1.3.3**). The shaded intervals indicate the standard deviation from 20 simulations with different realisations of the static disorder and the solid lines indicate the mean value.

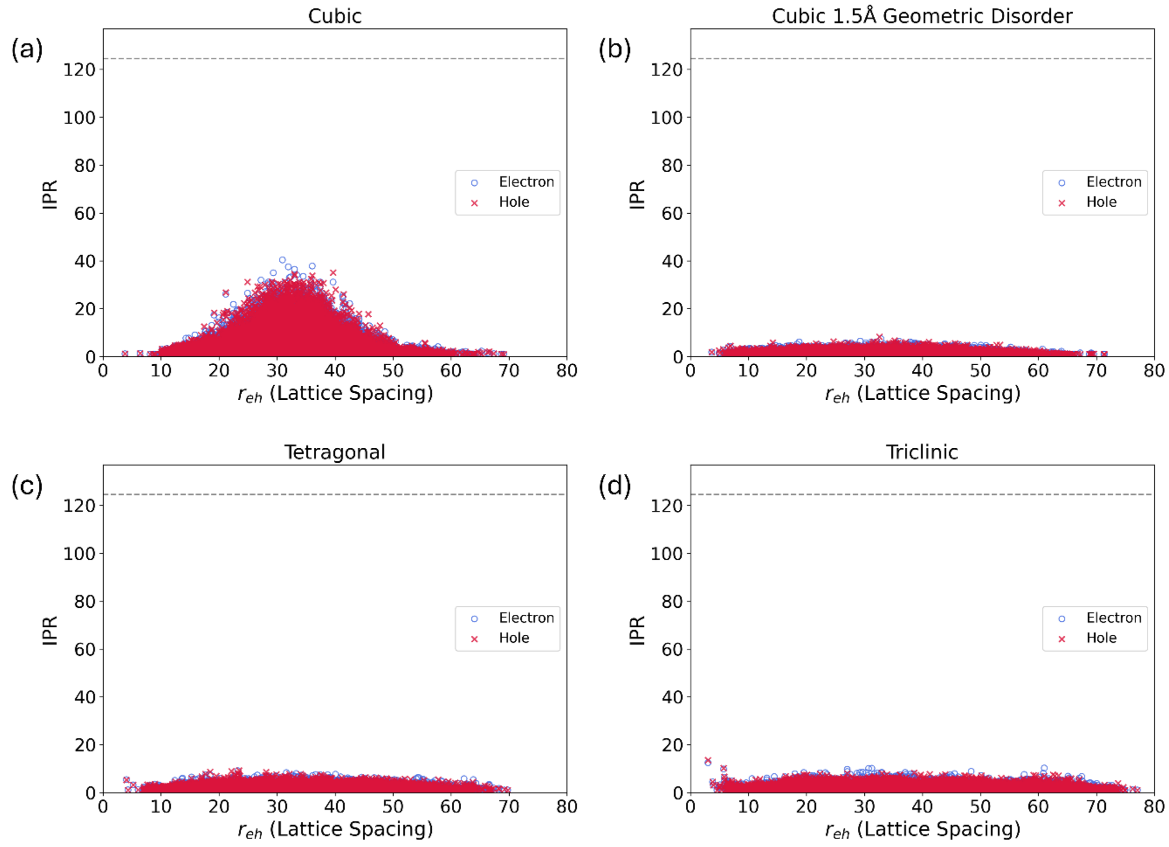

Supplementary Figure 40. Inverse participation ratio (IPR) of electrons and holes at no applied field for (a) the simple cubic lattice (b) the simple cubic lattice with 1.5 Å geometric disorder (c) the tetragonal lattice and (d) the triclinic lattice. Lattice structures are shown in **Supplementary Figure 6**. The simple cubic lattice has the most delocalised charges since its high symmetry leads to isotropic couplings.

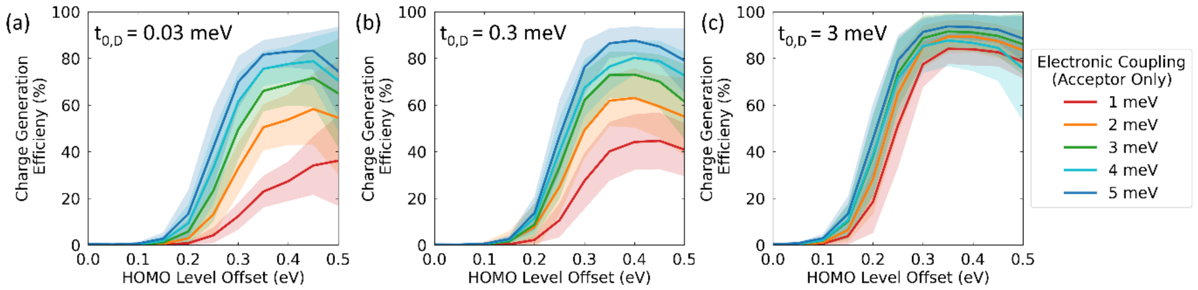

Supplementary Figure 41. Effect of the excitonic coupling in the donor domain on charge generation efficiency as the electronic coupling in the acceptor is varied. When the electronic coupling in the donor is comparable to that in the acceptor, varying the acceptor's electronic coupling has a minimal effect on the CGE as holes are efficiently transported away from the donor:acceptor interface, minimising recombination losses via the CT state. The shaded intervals indicate the standard deviation from 20 simulations with different realisations of the static disorder and the solid lines indicate the mean value.

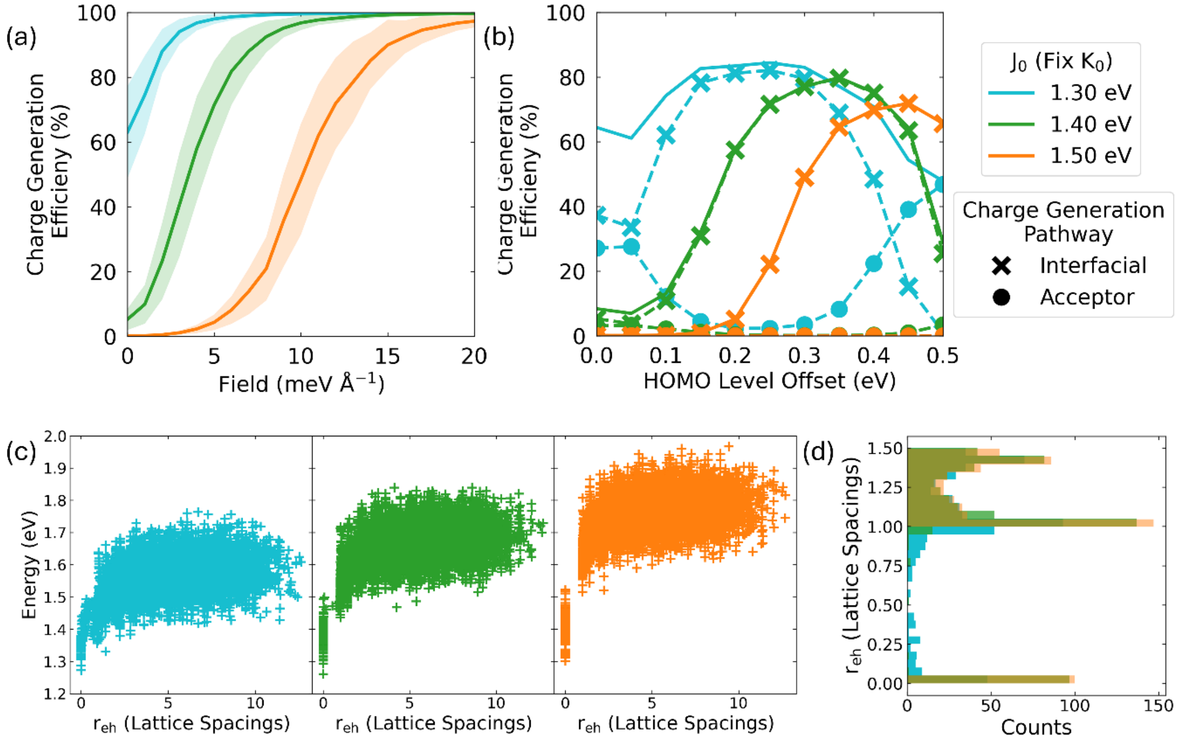

Supplementary Figure 42. (a) CGE versus field and (b) HOMO level offset for varying J_0 while K_0 is held constant. *In panel (a).* the shaded intervals indicate the standard deviation from 20 simulations with different realisations of the static disorder and the solid lines indicate the mean value. We have gone to smaller J_0 than used in the main text (J_0 = 1.30 eV corresponds to an exciton binding energy of 0.2 eV) to illustrate how, within the framework of our model, CGE from the acceptor domain only contributes to bilayer CGE at low offsets when charge generation across the interface is inefficient and that the CGE of the bilayer at low HOMO level offset is comparable to that achieved by the corresponding single component device at zero field. As we show in panels (c) and (d), decreasing J_0 in this way also lowers the energetic offset between local exciton (LE) and nearest-neighbour charge transfer (CT) basis states, increasing their hybridisation. It has been suggested that this type of hybridisation facilitates charge generation in Y6, which would be consistent with what we show here. (c) Distribution of eigenstate energies versus electron-hole separations (r_{eh}) for the values of J_0 shown in panels (a) and (b). (d) Histogram showing how the distribution of states with r_{eh} less than 1.5 lattice spacings changes as J_0 decreases. There is an increase in the number of states with r_{eh} between 0 and 1 for the smallest values of J_0, indicating an increase in the number of states with partial CT character. All simulation results shown in (c) and (d) are from single component simulations with no applied field.

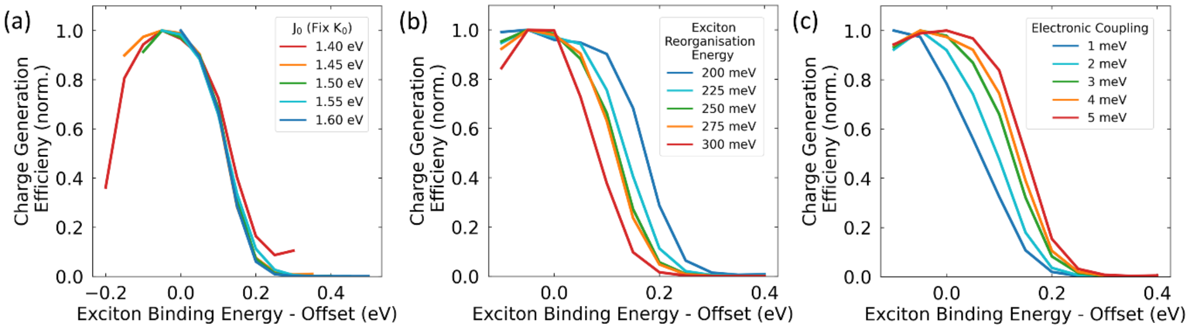

Supplementary Figure 43. CGE of the planar heterojunctions shown in **Figure 6a-c** of the main text plotted as a function of the driving force for exciton splitting (defined to be exciton binding energy – HOMO level offset) for simulations for varying (a) exciton binding energy (achieved via changing J_0, see **Section 1.1**), (b) exciton reorganisation energy and (c) electronic coupling. These figures illustrate how, for fixed values of the electronic coupling and exciton reorganisation energy, the (normalised) CGE is only a function of the driving force for exciton splitting.

6 Supplementary Tables

Material	Jsc (mA/cm2)	Voc (V)	FF	PCE (%)
ITDM	0.13	0.96	0.32	0.04
ITM	0.09	0.98	0.35	0.03
ITIC	0.13	1.02	0.27	0.04
IT4F	0.22	0.84	0.37	0.07
IDIC	0.05	0.88	0.42	0.02
Y6	0.50	0.82	0.35	0.14
Y5	0.18	0.97	0.32	0.05
Y16	0.71	0.76	0.32	0.17
Y1	2.06	0.91	0.30	0.57
Y11	0.35	0.88	0.34	0.10
IDTBR	0.041	1.1	0.36	0.02
IDFBR	0.085	1.2	0.25	0.03

Supplementary Table 1. JV parameters of single component devices

**Material**	**Ionisation Potential (eV)**
CuSCN	5.40 ± 0.03
2PACZ-Br	5.95 ± 0.03
CuSCN + 2PACZ-Br	5.53 ± 0.03
Y16	5.39 ± 0.03
Y1	5.40 ± 0.03
Y5	5.43 ± 0.03
Y11	5.47 ± 0.03
Y6	5.53 ± 0.03
BTP-ec9	5.54 ± 0.03
ITIC-DM	5.54 ± 0.03
C8-ITIC	5.55 ± 0.03
ITM	5.57 ± 0.03
ITIC	5.59 ± 0.03
IT4F	5.70 ± 0.03
IDIC	5.73 ± 0.03

Supplementary Table 2. Ionisation potentials measured with Ambient Pressure Photo Emission Spectra measured on KP Technology Ltd's APS02 system. Measurement data and fittings are shown in **Supplementary Supplementary Figure** 33. Ambient Pressure Photo Emission Spectra measured on KP Technology Ltd's APS02 system. The Ionisation energy is given by the intersection between the background level and the linear fit of the cube root of the emission tail.

Parameter	Value
Polaron Reorganisation Energy [a]	250 meV
Exciton Reorganisation Energy	250 meV
$t_{0,A}$	3 meV
$t_{0,D}$	3 meV
$t_{0,DA}$	0.03 meV
$d_{0,A}$	5 meV
$d_{0,D}$	5 meV
$d_{0,DA}$	5 meV
r_{0d}	1 Å
J_0	1.5 eV
r_{0j}	1 Å
Lattice Spacing	10 Å
Acceptor Bandgap	1.8 eV
Acceptor HOMO	0.0 eV
Acceptor Exciton Binding Energy	0.4 eV
Donor Bandgap	3.0 eV
Donor HOMO	ΔIP (variable)
Donor Exciton Binding Energy	0.5 eV
Exciton Coupling to Ground	20 meV
CT Coupling to Ground	1 meV
Extraction Rate	10^{11} s^{-1}
Gaussian (Static) Disorder	50 meV

[a] As we assume that each site has its own, independent phonon bath, the reorganisation energy associated with a charge transfer state will be twice the polaron reorganisation energy.

Supplementary Table 3: Default values of parameters used in the modelling. Shaded rows indicate parameters which are only relevant to bilayer simulations.

Parameter	Y6	ITIC	IDTBR	Reference
Exciton Binding Energy (eV)	0.26	0.23	0.43	[24]
	0.16	0.10	0.35	[25]
Exciton Reorganisation Energy (meV)	197	229	-	[26]
	86	-	-	[20]
	103	128	154/252*	[27]
	-	-	330	[21]
	242	292	396*	[28]
Electronic Coupling (meV)	21.2	18.0	-	[26]
	27.0-79.8	-	-	[20]
	-	2.8-17.1	-	[29]
	-	-	0.5-61	[30]
	49-132	32	1-103	[31]

*Calculations performed on EH-IDTBR. Ref. 27 found two distinct populations of singlet EH-IDTBR excitons and the reorganisation energies for both populations are quoted.

Supplementary Table 4: Summary of literature values for the exciton binding energy, exciton reorganisation energy and electronic couplings relevant to electron transport for Y6, ITIC and IDTBR. When a range of electronic couplings is given, this represents the spread in values for different dimer pairs within the crystal structure of the NFA.

Total reorganisation Energy (meV)	Inner reorganisation Energy (meV)	Outer Reorganisation Energy (meV)
200	161	39
225	182	42
250	202	48
275	222	53
300	242	58

Supplementary Table 5: Details of how the total reorganisation energy is split between inner and outer components in accordance with the definition of the spectral density function given in **Section 1.1**.

Abbreviation	Full Name
IDIC	2,2'-((2Z,2'Z)-((4,4,9,9-tetrahexyl-4,9-dihydro-s-indaceno[1,2-b:5,6-b']dithiophene-2,7-diyl)bis(methanylylidene))bis(3-oxo-2,3-dihydro-1H-indene-2,1-diylidene))dimalononitrile
ITIC	3,9-bis(2-methylene-(3-(1,1-dicyanomethylene)-indanone))-5,5,11,11-tetrakis(4-hexylphenyl)-dithieno[2,3-d:2’,3’-d’]-s-indaceno[1,2-b:5,6-b’]dithiophene
IT-4F	3,9-bis(2-methylene-((3-(1,1-dicyanomethylene)-6,7-difluoro)-indanone))-5,5,11,11-tetrakis(4-hexylphenyl)-dithieno[2,3-d:2’,3’-d’]-s-indaceno[1,2-b:5,6-b’]dithiophene
C8-ITIC	{(2Z)-2-[(8-{(E)-[1-(Dicyanomethylidene)-3-oxo-1,3-dihydro-2H-inden-2-ylidene]methyl}-6,6,12,12-tetraoctyl-6,12-dihydrothieno[3,2-b]thieno[2″,3″:4′,5′]thieno[2′,3′:5,6]-s-indaceno[2,1-d]thiophen-2-yl)methylidene]-3-oxo-2,3-dihydro-1H-inden-1-ylidene}propanedinitrile
IT-M	3,9-bis(2-methylene-((3-(1,1-dicyanomethylene)-6/7-methyl)-indanone))-5,5,11,11-tetrakis(4-hexylphenyl)-dithieno[2,3-d:2’,3’-d’]-s-indaceno[1,2-b:5,6-b’]dithiophene
IT-DM	3,9-bis(2-methylene-((3-(1,1-dicyanomethylene)-6,7-dimethyl)-indanone))-5,5,11,11-tetrakis(4-hexylphenyl)-dithieno[2,3-d:2’,3’-d’]-s-indaceno[1,2-b:5,6-b’]dithiophene
Y6	2,2'-((2Z,2'Z)-((12,13-bis(2-ethylhexyl)-3,9-diundecyl-12,13-dihydro-[1,2,5]thiadiazolo[3,4-e]thieno[2",3’':4’,5']thieno[2',3':4,5]pyrrolo[3,2-g]thieno[2',3':4,5]thieno[3,2-b]indole-2,10-diyl)bis(methanylylidene))bis(5,6-difluoro-3-oxo-2,3-dihydro-1H-indene-2,1-diylidene))dimalononitrile
Y5	(2,2’-((2Z,2’Z)-((12,13-bis(2-ethylhexyl)-3,9-diundecyl-12,13-dihydro[1,2,5]thiadiazolo[3,4e]thieno[2”,3”:4’,5’]thieno[2’,3’:4,5]pyrrolo[3,2-g] thieno[2’,3’:4,5]thieno[3,2-b]indole-2,10-diyl)bis(methanylylidene))bis(3-oxo-2,3-dihydro1H-indene-2,1-diylidene))dimalononitrile)
BTP-eC9	2,2'-[[12,13-Bis(2-butyloctyl)-12,13-dihydro-3,9-dinonylbisthieno[2",3":4',5']thieno[2',3':4,5]pyrrolo[3,2-e:2',3'-g][2,1,3]benzothiadiazole-2,10-diyl]bis[methylidyne(5,6-chloro-3-oxo-1H-indene-2,1(3H)-diylidene)]]bis[propanedinitrile]
Y1	-
Y11	2,2'-((2Z,2'Z)-((6,12,13-Tris(2-ethylhexyl)-3,9-diundecyl-12,13-dihydro-6H-thieno[2",3":4',5']thieno[2',3':4,5]pyrrolo[3,2-g]thieno[2',3':4,5]thieno[3,2-b][1,2,3]triazolo[4,5-e]indole-2,10-diyl)bis(methanylylidene))bis(5,6-difluoro-3-oxo-2,3-dihydro-1H-indene-2,1-diylidene))dimalononitrile
Y16	-
o-IDFBR	(5Z,5'Z)-5,5'-((7,7'-(6,6,12,12-Tetraoctyl-6,12-dihydroindeno[1,2-b]fluorene-2,8-diyl)bis(benzo[c][1,2,5]thiadiazole-7,4-diyl))bis(methanylylidene))bis(3-ethyl-2-thioxothiazolidin-4-one)
o-IDTBR	(5Z,5'Z)-5,5'-((7,7'-(4,4,9,9-tetraoctyl-4,9-dihydro-s-indaceno[1,2-b:5,6-b']dithiophene-2,7-diyl)bis(benzo[c][1,2,5]thiadiazole-7,4-diyl))bis(methanylylidene))bis(3-ethyl-2-thioxothiazolidin-4-one)

Supplementary Table 6: Full names of the small molecule acceptors used in this work. We note that names for Y1 and Y16 could not be found, but their structures are shown in **Supplementary Figure 22**.

References

1 Zhang, G. *et al.* Delocalization of exciton and electron wavefunction in non-fullerene acceptor molecules enables efficient organic solar cells. *Nature Communications* **11**, 3943, doi:10.1038/s41467-020-17867-1 (2020).

2 Kahle, F.-J. *et al.* Static and Dynamic Disorder of Charge Transfer States Probed by Optical Spectroscopy. *Advanced Energy Materials* **12**, 2103063, doi:https://doi.org/10.1002/aenm.202103063 (2022).

3 Lankevich, V. & Bittner, E. R. Relating free energy and open-circuit voltage to disorder in organic photovoltaic systems. *The Journal of Chemical Physics* **149**, 244123, doi:10.1063/1.5050506 (2018).

4 Roberts, G. & Warren, K. D. Justification of the Mmataga-Nishimoto approximation. *Theoretica chimica acta* **13**, 353-354, doi:10.1007/BF00529027 (1969).

5 Li, P. *et al.* Synergistic Effect of Dielectric Property and Energy Transfer on Charge Separation in Non-Fullerene-Based Solar Cells. *Angewandte Chemie International Edition* **60**, 15054-15062, doi:https://doi.org/10.1002/anie.202103357 (2021).

6 Kasha, M., Rawls, H. R. & El-Bayoumi, M. A. The exciton model in molecular spectroscopy. *Pure and Applied Chemistry* **11**, 371-392, doi:doi:10.1351/pac196511030371 (1965).

7 Renger, T. & Marcus, R. A. On the relation of protein dynamics and exciton relaxation in pigment–protein complexes: An estimation of the spectral density and a theory for the calculation of optical spectra. *The Journal of Chemical Physics* **116**, 9997-10019, doi:10.1063/1.1470200 (2002).

8 May, V. & Kuhn, O. in *Charge and Energy Transfer Dynamics in Molecular Systems* 59-176 (2023).

9 Cupellini, L., Corbella, M., Mennucci, B. & Curutchet, C. Electronic energy transfer in biomacromolecules. *WIREs Computational Molecular Science* **9**, e1392, doi:https://doi.org/10.1002/wcms.1392 (2019).

10 Yang, M. & Fleming, G. R. Influence of phonons on exciton transfer dynamics: comparison of the Redfield, Förster, and modified Redfield equations. *Chemical Physics* **275**, 355-372, doi:https://doi.org/10.1016/S0301-0104(01)00540-7 (2002).

11 Balzer, D. & Kassal, I. Even a little delocalization produces large kinetic enhancements of charge-separation efficiency in organic photovoltaics. *Science Advances* **8**, eabl9692, doi:10.1126/sciadv.abl9692 (2022).

12 Balzer, D. & Kassal, I. Delocalisation enables efficient charge generation in organic photovoltaics, even with little to no energetic offset. *Chemical Science* **15**, 4779-4789, doi:10.1039/D3SC05409H (2024).

13 Xu, D. & Cao, J. Non-canonical distribution and non-equilibrium transport beyond weak system-bath coupling regime: A polaron transformation approach. *Frontiers of Physics* **11**, 110308, doi:10.1007/s11467-016-0540-2 (2016).

14 Sumi, H. Theory on Rates of Excitation-Energy Transfer between Molecular Aggregates through Distributed Transition Dipoles with Application to the Antenna System in Bacterial Photosynthesis. *The Journal of Physical Chemistry B* **103**, 252-260, doi:10.1021/jp983477u (1999).

15 Taylor, N. B. & Kassal, I. Generalised Marcus theory for multi-molecular delocalised charge transfer. *Chemical Science* **9**, 2942-2951, doi:10.1039/C8SC00053K (2018).

16 Green, M. A. Solar cell fill factors: General graph and empirical expressions. *Solid-State Electronics* **24**, 788-789, doi:https://doi.org/10.1016/0038-1101(81)90062-9 (1981).

17 Baumeier, B., Kirkpatrick, J. & Andrienko, D. Density-functional based determination of intermolecular charge transfer properties for large-scale morphologies. *Physical Chemistry Chemical Physics* **12**, 11103-11113, doi:10.1039/C002337J (2010).

18 Madjet, M. E., Abdurahman, A. & Renger, T. Intermolecular Coulomb Couplings from Ab Initio Electrostatic Potentials: Application to Optical Transitions of Strongly Coupled Pigments in Photosynthetic Antennae and Reaction Centers. *The Journal of Physical Chemistry B* **110**, 17268-17281, doi:10.1021/jp0615398 (2006).

19 Lu, T. & Chen, F. Multiwfn: A multifunctional wavefunction analyzer. *Journal of Computational Chemistry* **33**, 580-592, doi:https://doi.org/10.1002/jcc.22885 (2012).

20 Giannini, S. *et al.* On the role of charge transfer excitations in non-fullerene acceptors for organic photovoltaics. *Materials Today*, doi:https://doi.org/10.1016/j.mattod.2024.09.009 (2024).

21 Rezasoltani, E. *et al.* Correlating the Phase Behavior with the Device Performance in Binary Poly-3-hexylthiophene: Nonfullerene Acceptor Blend Using Optical Probes of the Microstructure. *Chemistry of Materials* **32**, 8294-8305, doi:10.1021/acs.chemmater.0c02093 (2020).

22 Rodríguez-Martínez, X. *et al.* Predicting the photocurrent–composition dependence in organic solar cells. *Energy & Environmental Science* **14**, 986-994, doi:10.1039/D0EE02958K (2021).

23 Yan, J. *et al.* Identifying structure–absorption relationships and predicting absorption strength of non-fullerene acceptors for organic photovoltaics. *Energy & Environmental Science* **15**, 2958-2973, doi:10.1039/D2EE00887D (2022).

24 Sugie, A., Nakano, K., Tajima, K., Osaka, I. & Yoshida, H. Dependence of Exciton Binding Energy on Bandgap of Organic Semiconductors. *The Journal of Physical Chemistry Letters* **14**, 11412-11420, doi:10.1021/acs.jpclett.3c02863 (2023).

25 Bertrandie, J. *et al.* The Energy Level Conundrum of Organic Semiconductors in Solar Cells. *Advanced Materials* **34**, 2202575, doi:10.1002/adma.202202575 (2022).

26 Kupgan, G., Chen, X. K. & Brédas, J. L. Molecular packing of non-fullerene acceptors for organic solar cells: Distinctive local morphology in Y6 vs. ITIC derivatives. *Materials Today Advances* **11**, 100154, doi:https://doi.org/10.1016/j.mtadv.2021.100154 (2021).

27 Kashani, S., Wang, Z., Risko, C. & Ade, H. Relating reorganization energies, exciton diffusion length and non-radiative recombination to the room temperature UV-vis absorption spectra of NF-SMA. *Materials Horizons* **10**, 443-453, doi:10.1039/D2MH01228F (2023).

28 Caruso, D. & Troisi, A. Long-range exciton dissociation in organic solar cells. *Proceedings of the National Academy of Sciences* **109**, 13498-13502, doi:10.1073/pnas.1206172109 (2012).

29 Aldrich, T. J. *et al.* Fluorination Effects on Indacenodithienothiophene Acceptor Packing and Electronic Structure, End-Group Redistribution, and Solar Cell Photovoltaic Response. *Journal of the American Chemical Society* **141**, 3274-3287, doi:10.1021/jacs.8b13653 (2019).

30 Stojanović, L. *et al.* Disorder-Induced Transition from Transient Quantum Delocalization to Charge Carrier Hopping Conduction in a Nonfullerene Acceptor Material. *Physical Review X* **14**, 021021, doi:10.1103/PhysRevX.14.021021 (2024).

31 Liu, W. & Andrienko, D. An ab initio method on large sized molecular aggregate system: Predicting absorption spectra of crystalline organic semiconducting films. *The Journal of Chemical Physics* **158**, 094108, doi:10.1063/5.0138748 (2023).
